# A supercharged molecular motor operating by constitutional alteration and proton transfer

**DOI:** 10.1038/s41557-026-02141-6

**Published:** 2026-06-03

**Authors:** Pronay Kumar Biswas, Ani Ozcelik, Martina Hartinger, Nico Groß, Frank Hampel, Carolin Müller, Henry Dube

**Affiliations:** 1 Department of Chemistry and Pharmacy, Friedrich-Alexander-Universität Erlangen-Nürnberg, Erlangen, Germany; 2 Computer-Chemie-Centrum, Friedrich-Alexander-Universität Erlangen-Nürnberg, Erlangen, Germany

**Keywords:** Organic chemistry, Photochemistry, Molecular machines and motors

## Abstract

Light-driven molecular motors undergo directional motions upon the input of external energy and represent archetypical molecular machines. So far, such motors have functioned via light-induced bond rotations, where directionality is dictated by a fixed source of asymmetry. During the operation cycle, no further structural changes occur, other than the rotation itself. Here we disclose a highly effective mechanism for light-driven motor rotation involving constitutional alteration and reversible proton transfer. Associated with this unusual mechanism is a particularly high energy content from the incident light that the motor retains. This feature is further exploited in a low-temperature molecular solar thermal energy storage application, where solar energy can be stored and released in a controlled fashion and tracked step by step with the naked eye. With these findings, unique possibilities emerge for the design and use of molecular motors with hitherto unknown modes of action and power.

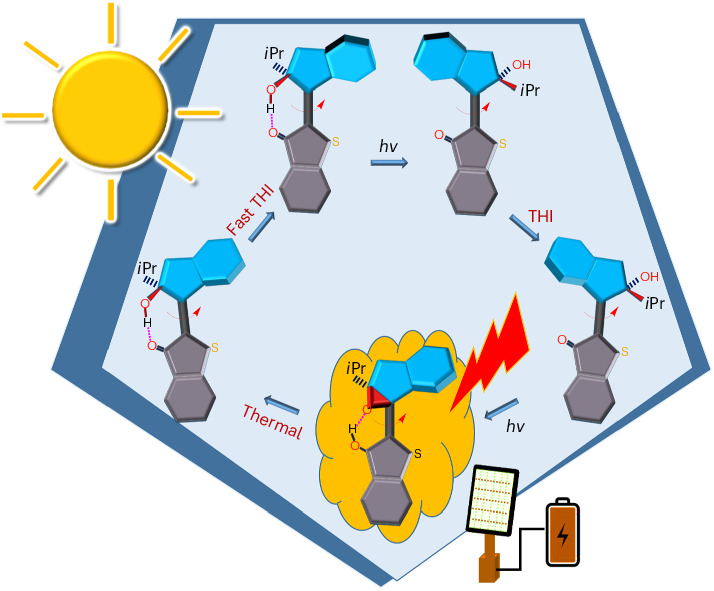

## Abstract (web summary)

Light-driven molecular motors represent archetypical molecular machines, with the existing varieties working via light-induced bond rotations. Now a different mechanism invoking constitutional alteration and reversible proton transfer is shown. This enables the formation of a high-energy intermediate and its application in low-temperature molecular solar thermal energy storage.

## Main

**Fig. 1:**
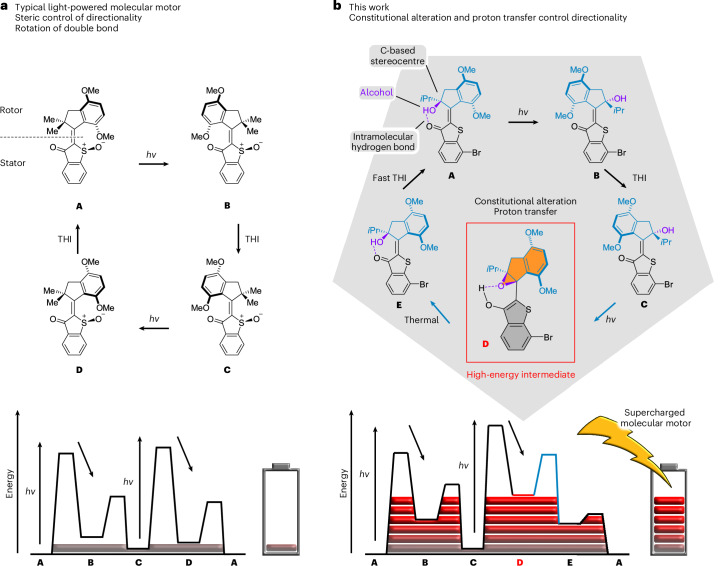
Light-powered molecular motors and their mechanism of action with associated energy profiles. **a**, Working mechanism of an HTI molecular motor akin to the second-generation Feringa motors. **b**, Molecular motor **1** invokes constitutional alteration and proton transfer during rotation. Its unidirectional motion proceeds in five steps highlighted by the shaded pentagon. The rotor fragment of motor **1** bears methoxy (OMe) groups as well as a hydroxy and isopropyl (*i*Pr) at the carbon stereocentre. The constitutional isomer (framed) represents a high-energy intermediate that stores a large amount of the light energy introduced in the preceding photoisomerization (*hv*) step.

Molecular motors have gained considerable traction as nanoscale powering units for advanced molecular machines and nanotechnology^1–5^. The central feature of molecular motors is their directional motion fueled by different sources of energy^6^. A variety of designs are now available^7–14^, with light-powered versions standing out because of their straightforward and waste-free energy supply^15–17^. After Feringa and co-workers developed the initial design of a working molecular motor in 1999^18^, second^19^ and third^20^ generations of the overcrowded alkene-based motors were introduced by the same group. The common working mechanism of these Feringa-type motors comprises two photoisomerization steps that are intersected by ratcheting thermal helix inversion (THI) steps. Several alternative designs for light-driven molecular motors have been developed since then. In 2014, Greb and Lehn presented an imine-based molecular motor system in which the thermal ratcheting steps are imine-inversion or ring-flip processes instead of THI^21,22^. In 2015, our group presented a hemithioindigo (HTI)-based molecular motor that can be powered by visible light and works by a mechanism closely related to the Feringa motors (Fig. 1a)^23–25^. In 2018, we established a photon-only molecular motor that does not require thermal steps but instead involves three consecutive irradiation steps^26^. Subsequently, we reported on yet another different type of molecular motor that performs a directional figure-of-eight-shaped motion^27^. Further designs for light-driven molecular motors have since emerged, including heterocyclic derivatives^28–30^, biomimetic artificial motors^31,32^ and a number of theoretically predicted versions^33–40^.

In principle, it is possible to imagine other sources of directionality control in light-driven molecular motors and weak interactions have been discussed for some time in this regard. For example, hydrogen bonding was identified as a very interesting candidate for controlling directionality in a theoretical study by the groups of Sampedro and Frutos as early as 2013^41^. Related endeavours have put forward intermolecular hydrogen-bonding interactions to remotely control the directionality of a motor^42,43^. More recently a molecular motor with intramolecular hydrogen bonding was disclosed by the groups of Crespi and Feringa using a chiral hydroxyindanone rotor and a barbituric acid stator^44^.

Here we report a molecular motor **1** that employs a similar intramolecular hydrogen-bonding interaction to control light-powered unidirectional rotation (Fig. 1b). However, in contrast to all earlier described molecular motor mechanisms, we found that motor **1** undergoes a five-step motion cycle involving hitherto unknown constitutional alteration steps. In this process, light-induced double bond isomerization is tied to intramolecular epoxide formation and proton transfer, producing a high-energy intermediate that stores a substantial amount of the incident light energy (Fig. 1b). We further show how motor **1** can be used for applications in molecular solar thermal (MOST) energy storage.

## Results and discussion

### Design rationale and structural modifications

The source of asymmetry in all HTI-based molecular motors to have been reported so far is a sulfoxide with point chirality instead of a carbon stereogenic centre^23–27,45,46^. For HTI motor **1**, we used as the asymmetry source a carbon-based stereocentre that resides on the indanone instead of the thioindigo fragment (Fig. 1b). This stereogenic centre places an alcohol moiety close to the central double bond—the axis of directional rotation—which can form intramolecular hydrogen bonds with the stator fragment. The sulfur atom in the thioindigo fragment is not oxidized and is expected to lead to a notable redshift of the absorption compared with established HTI-based motors. A bromide was introduced into the thioindigo fragment for the dual purpose of facilitating synthesis (halogenated benzothiophenones are more stable and less prone to form thioindigo as a side product) and introducing a convenient synthetic handle for late-stage functionalization and applications.

### Motor 1 ground-state calculations

**Fig. 2:**
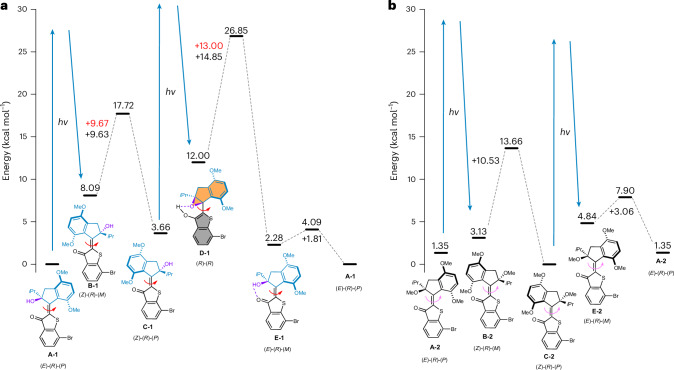
Ground-state theoretical analysis of the motor rotation mechanism of 1 and methoxy derivative 2. **a**, Analysis of the rotation mechanism of the hydrogen-bonded molecular motor **1**. Experimentally determined Gibbs energies of activation are shown in red for comparison. The profile reveals proper motor behaviour, a supercharged and constitutionally altered intermediate **D-1**, and a five-step operation cycle. **b**, Analysis of the rotation mechanism of the methoxy derivative **2**. The profile also reveals proper motor behaviour but adheres to the classical four-step cycle, providing less storage capacity for light energy. The calculations were performed at the CAM-B3LYP/6-311 G(d,p)/PCM(THF) level of theory.

A theoretical analysis using density functional theory (DFT) was first conducted to elucidate the motor rotation mechanism of **1** (Fig. 2a and Supplementary Tables 1 and 2). Direct comparison was made with ether derivative **2** lacking hydrogen bonding and proton transfer capacity (Fig. 2b and Supplementary Table 4). Motor **1** progresses through five distinct intermediates **A**–**E** during its rotation (accessible to both (*R*)- and (*S*)-configured stereoisomers; for clarity, only the (*R*)-configured structures are discussed in the following; Fig. 2a and Supplementary Fig. 1). The global minimum structure **A-1** possesses an (*E*)-configured double bond and allows intramolecular hydrogen bonding between the hydroxy group and carbonyl function. Following the (*P*) helicity of this structure, photoisomerization in a counterclockwise manner leads to the population of **B-1** with a (*Z*)-configured double bond and (*M*) helicity. During this photoconversion, the intramolecular hydrogen bond with the carbonyl is disrupted and there is no notable hydrogen-bonding interaction with the sulfur atom in **B-1**. A chalcogen-bonding interaction between the sulfur and carbonyl oxygen, as recently observed in heterocyclic HTI photoswitches^47^, is also unlikely in this case. According to theory, isomer **B-1** possesses a very high energy of 7.33/8.09 kcal mol^−1^ (B3LYP/6-311G(d,p)/PCM(THF) or CAM-B3LYP/6-311G(d,p)/PCM(THF), respectively) and is stabilized after THI (Fig. 2a and Supplementary Table 1). This THI step continues the directional rotation to populate isomer **C-1** with a (*Z*)-configured double bond and (*P*) helicity and serves as a ratcheting step in the motor mechanism. A transition state with an energy 9.6 kcal mol^−1^ higher than **B-1** is predicted for this process. The energy of isomer **C-1** is predicted to be lower than **B-1** by 4.43 kcal mol^−1^, leading to complete conversion under ambient to high temperatures. Still, the stabilized isomer **C-1** possesses a significantly higher energy than the global minimum by 3.7 kcal mol^−1^ (Fig. 2a). These energies are among the highest amounts of photoenergy stored in metastable HTI motor systems and switches reported so far and would lead to the complete thermal conversion of **C-1** to **A-1** at elevated temperatures. The sequence of isomer interconversions from **A** to **B** to **C** represents the first 180° unidirectional rotation of motor **1**. We expected a similar two-step interconversion between three isomers for the second 180° rotation instead of the population of an additional and highly unusual epoxide intermediate **D-1** (Fig. 2a). Photoirradiation of **C-1** would thus continue rotation in a counterclockwise sense, leading to the population of metastable isomer **E-1** with an (*E*)-configured double bond and (*M*) helicity. Intramolecular hydrogen bonding with the carbonyl would be re-established in this step. Isomer **E-1** was calculated to be 2.3 kcal mol^−1^ higher in energy than **A-1** and could thus be converted completely into the latter in another THI step. In this process, intramolecular hydrogen bonding is retained and the predicted transition state is only 1.8 kcal mol^−1^ higher in energy than **E-1** (Fig. 2a). With such a low transition-state energy, experimental observation of the thermal conversion of **E-1** to **A-1** would not be possible without ultrafast transient spectroscopy methods. Contradicting this initial theoretical assessment, we found a conveniently stable intermediate experimentally that decayed completely to **A-1** with an associated Gibbs energy of activation (Δ*G*^‡^) of 13.0 kcal mol^−1^. As the experimental (see below) and theoretical spectra did not match with the expected isomer **E-1**, we returned to a more comprehensive theoretical assessment. Two options were taken into account, guided by the strong hypsochromic shift experimentally observed in the UV–visible absorption spectrum. To explain this behaviour, we assumed that the conjugation via the central double bond is broken in the respective intermediate and that either an epoxide or furan-based structure is formed. The calculated epoxide structure **D-1** matches very well both energetically and spectroscopically with the experimentally observed intermediate (see below and Supplementary Sections 3.1–3.3 and 4.1–4.3). **D-1** is formed by a conjugated intramolecular epoxidation reaction while the hydroxy proton shifts to the former carbonyl—and now enolic—site of the thioindigo fragment. An effective intramolecular hydrogen bond is formed between the enol and the epoxide, stabilizing the structure in a nearly perpendicular arrangement of the indanone rotor and thioindigo stator (Supplementary Fig. 4). At the same time, the thioindigo fragment is fully aromatized. Although **D-1** possesses a single bond instead of the central HTI double bond, rotation towards another local minimum **D-1′** on the epoxide potential energy surface and also ‘backward’ motion to **C-1** are inhibited by high energy barriers (19.5 and 28.9 kcal mol^−1^, respectively; Supplementary Figs. 2–4). **D-1** thus represents a ‘trapped’ 90° rotation intermediate possessing an exceptionally high energy of 12 kcal mol^−1^ (Supplementary Table 2). This high energy, which comes directly from the incident light irradiation, is stored in the intermediate and could thus be expended to do actual work in the subsequent thermally activated steps. It thus establishes a true supercharging process in a light-driven molecular motor. The next thermally activated step is calculated to comprise concomitant epoxide opening and reverse proton transfer, tied to further rotation to reach intermediate **E-1**. The initial direction of rotation is maintained in this step and an associated Δ*G*^‡^ = 14.9 kcal mol^−1^ is predicted, which is significantly lower than the rotational barriers leading to **D-1′** or **C-1**. Given that only continued rotation to **E-1** allows for reverse proton transfer and the re-establishment of the central double bond, unidirectionality would be very effectively ratcheted in this step. The final THI step leading from **E-1** to **A-1** was found in the initial calculations as described above. With this theoretically predicted energy profile, quantitative directionality is expected for motor **1** even at higher temperatures. Experiments at low temperatures should allow four different isomers, that is, **A-1**, **B-1**, **C-1** and **D-1**, to be observed and thus evidence unidirectionality directly.

To complete the theoretical analysis, we investigated the spectroscopic properties (NMR, UV–visible and electronic circular dichroism (ECD)) of all observable isomers and also non-covalent interactions (Supplementary Section 3.5, Supplementary Figs. 5–14 and Supplementary Tables 3–6).

### Theoretical comparison with hydrogen-bond-deficient derivative 2

A similar ground-state theoretical analysis was conducted for trimethoxy-substituted derivative **2** lacking intramolecular hydrogen bonding. The theoretically calculated energy profile suggests that **2** would also classify as a motor, but it adheres to the classical four-step cycle (including states **A-2**, **B-2**, **C-2** and **E-2**) with alternating photochemical and THI steps (Fig. 2b). The sense of directionality is the same as in motor **1**, showing directly that hydrogen bonding is not responsible for the unidirectionality within the classical parts of the rotation mechanism. In addition, for derivative **2**, the **C-2** isomer is the global minimum and significantly less energy is stored in the metastable states **B-2** (3.13 kcal mol^−1^) and **E-2** (4.84 kcal mol^−1^; Supplementary Table 4). With this energy profile, full unidirectionality would still be present. As for **1**, the most stable structures **A-2** and **C-2** feature a *syn* relationship between the large isopropyl group and the aromatic methoxy group in *ortho* position with respect to the central double bond. It thus becomes clear that size differences at the stereogenic centre are primarily responsible for dictating the sense of directionality of **1** and **2**.

### Computational analysis of the light-induced C-1 → D-1 transformation

**Fig. 3:**
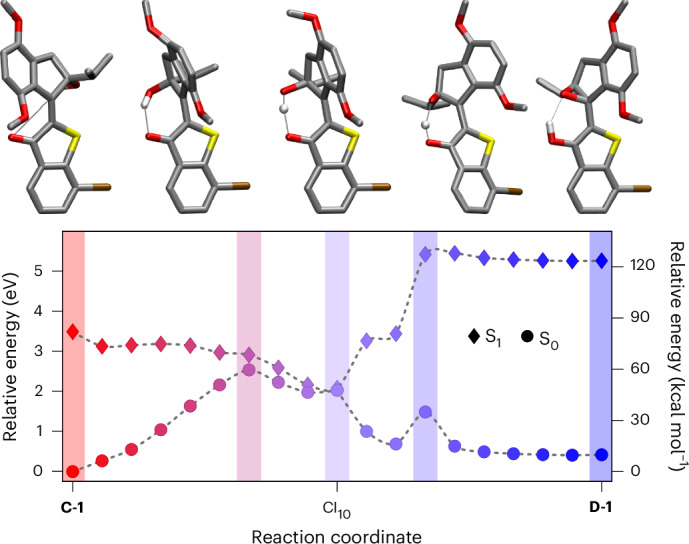
Theoretical description of the photochemical C -1 → D-1 conversion. The photochemical conversion of **C-1** to **D-1** was investigated theoretically by MRSF-TDDFT (**C-1** → CI_10_ segment and S_1_ curve of CI_10_→ **D-1**) and (TD)DFT (S_0_ curve of CI_10_ → **D-1**). Mechanistically, an excited-state double bond rotation on the S_1_ energy surface is revealed, which triggers epoxide formation and proton transfer upon return to the ground state S_0_ through the conical intersection CI_10_. The positions in the energy diagram of the structures shown above the plot are indicated by the coloured bars. Source data

To gain deeper insight into the photochemical reaction pathway from **C-1** to **D-1**, we conducted a comprehensive theoretical investigation (for related excited-state studies of molecular motors, see, for example, refs. ^25,31,32,34,36–39,45,48–52^). To this end, we optimized the S_1_/S_0_ conical intersection (CI_10_) using spin-flip and mixed-reference spin-flip time-dependent DFT (SF-TDDFT and MRSF-TDDFT, respectively). We verified the use of these methods by comparing our obtained CI structures for the HTI parent structure with complete active space self-consistent field (CASSCF) reference structures (Supplementary Fig. 15). The resulting CI_10_ geometry (Fig. 3 and Supplementary Fig. 16) exhibits a torsion angle of 99° (S–C=C–C), characteristic of double bond rotation. A hydrogen bond with a length of 1.69 Å is observed between the hydroxy proton and the carbonyl oxygen atom, while the O–H bond length remains at 0.99 Å, indicating that proton transfer has not yet occurred. Unlike in HTI systems, no other minimum-energy CI structures with different degrees of pyramidalization or tilt could be optimized (Supplementary Fig. 17)^53^.

To further analyse the relaxation pathway, we optimized the minimum-energy path connecting **C-1** and **D-1** via CI_10_. As CI_10_ is accessed upon photoexcitation, the **C-1** → CI_10_ segment was optimized on the S_1_ surface, while the CI_10_ → **D-1** relaxation was examined separately on both the S_0_ and T_1_ surfaces. The computed potential energy curves (Fig. 3) show a general decrease in S_1_ energy towards CI_10_, except for a shallow local minimum (red to purple diamonds in Fig. 3). Close to CI_10_, S_0_ and T_1_ become nearly degenerate (Supplementary Fig. 18), suggesting a potential role of intersystem crossing^25^.

Following relaxation from CI_10_ to S_0_ (purple to blue path in Fig. 3), proton transfer from the hydroxy to the carbonyl group encounters an energy barrier of 19 kcal mol^−1^ in a vacuum, which decreases to 10 kcal mol^−1^ in aqueous solvent models. These findings indicate that epoxide formation occurs after relaxation to the ground state, facilitated by proton transfer (Supplementary Figs. 18 and 19). It thus becomes rather unlikely that an excited-state proton transfer mechanism is involved in epoxide formation, in contrast to the behaviour of indigo itself^54–56^ and other indigoid photoswitches bearing acidic protons^57^.

### Synthesis and characterization of motor 1 at elevated temperatures

Motor **1** was synthesized and characterized as described in the Methods and Supplementary Information (Supplementary Scheme 1 and Supplementary Figs. 20–34). Single crystals suitable for X-ray diffraction analysis were obtained for the two stable isomers, that is, the global minimum (*E*)-isomeric **A-1** and the metastable (*Z*)-isomeric **C-1**, providing direct evidence of the molecular structures and intramolecular hydrogen bonding in **A-1** (Fig. 4a). Deuterium exchange experiments allowed us to identify directly the ^1^H NMR signals of the OH proton in the different isomers of motor **1** (Fig. 4b).

**Fig. 4:**
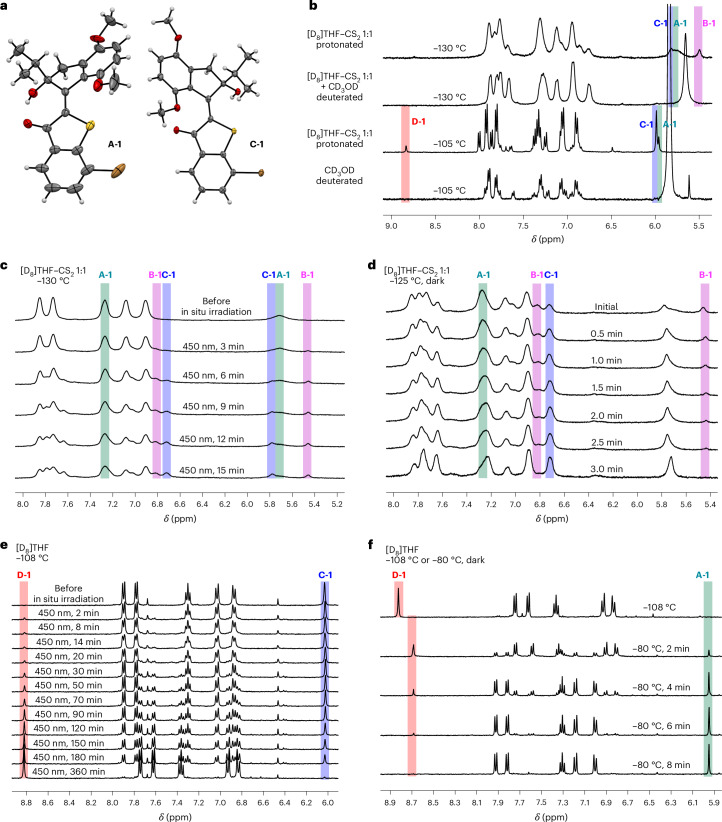
Single-crystal structures and ^1^H NMR spectroscopic analysis of motor 1. **a**, Oak Ridge thermal ellipsoid plot representations of the structures of **A-1** and **C-1**. **b**–**f**, The proper functioning of motor **1** was evidenced by establishing selective interconversions of its isomers, analysed by ^1^H NMR spectroscopy (400 MHz): ^1^H NMR spectra of motor **1** showing deuterium exchange of the OH group in different conditions (**b**); ^1^H NMR spectra of the photoconversion of **A-1** to **B-1** (**c**); ^1^H NMR spectra of the thermal conversion of **B-1** to **C-1** (**d**); ^1^H NMR spectra of the photoconversion of **C-1** to **D-1** (**e**); ^1^H NMR spectra of the thermal conversion of **D-1** to **A-1** (**f**). In **b**–**f**, the vertical coloured bands signify the individual isomers of motor **1**: **A-1** (green), **B-1** (pink), **C-1** (blue) and **D-1** (red). Source data

After successful synthesis, the thermal behaviour of motor **1** was scrutinized. At ambient temperature, only two isomers could be observed and fully characterized, that is, **A-1** and **C-1**, as predicted by theory. **C-1** was completely converted into **A-1** upon prolonged heating at 100 °C in [D_8_]toluene (Supplementary Fig. 35). When conservatively assuming that a remaining 2% of **C-1** cannot be detected by ^1^H NMR spectroscopy, the resulting equilibrium constant *K* = 98/2 at 100 °C corresponds to a Gibbs energy difference (Δ*G*) between **A-1** and **C-1** of 2.9 kcal mol^−1^. This value is the lower limit of the energy difference between the two isomers and thus the theoretically predicted value of 3.66 kcal mol^−1^ is supported by experiment. In addition, we analysed the corresponding kinetics of thermal *Z* to *E* isomerization in different solvents (Supplementary Figs. 36–40 and Supplementary Table 7). The results show that the directionality of the motor is not impeded by thermal double bond isomerization, even at 100 °C.

### Identification of motor states and isomer interconversions

Next, irradiation experiments with **A-1** and **C-1**, which had already been characterized by X-ray diffraction analysis (Supplementary Table 8), were conducted at low temperatures to elucidate the individual photoisomerization steps (Supplementary Figs. 41–50). Either tetrahydrofuran (THF) or THF–CS_2_ mixtures were used as solvent to reach the very low temperatures required to identify the direct photoproducts without sacrificing solubility. Starting from **A-1**, in situ irradiation at −130 °C in a 1:1 mixture of [D_8_]THF and CS_2_ revealed the initial formation of one new isomer as the direct photoproduct, and only subsequently the formation of isomer **C-1** (Fig. 4c and Supplementary Fig. 51). The isomer populated first did not accumulate to any extent even at this low temperature, but quickly reached a steady-state concentration while the population of **C-1** further increased upon continued irradiation. On switching off the light and raising the temperature slightly to −125 °C, the first formed isomer thermally converted exclusively to **C-1** (Fig. 4d and Supplementary Fig. 52). Kinetic analysis delivered a Gibbs energy of activation of Δ*G*^‡^ = 9.67 kcal mol^−1^ for this process (Supplementary Fig. 52). This is fully consistent with the predicted properties of isomer **B-1**, which could thus be assigned as the direct photoproduct of **A-1**. A complementary set of experiments was conducted to investigate the photoisomerization of **C-1**. Irradiation of **C-1** at −108 °C in [D_8_]THF resulted in the population of a fourth isomer, which accumulated almost quantitatively (Fig. 4e). A thorough ^1^H NMR analysis was conducted at low temperatures (−108 to −120 °C), including nuclear Overhauser effect experiments (Supplementary Figs. 53–57). Upon closer scrutiny, ^13^C distortionless enhancement by polarization transfer including the detection of quaternary nuclei (DEPTq) and two-dimensional NMR analyses (Supplementary Figs. 56 and 57) revealed the absence of a carbonyl carbon signal. Furthermore, signal shifts of the expected central double bond carbon atoms and the stereogenic carbon centre did not match their expected hybridization or substitution character. Thermal annealing of the new isomer at −80 °C in the dark led to full conversion to **A-1** (Fig. 4f). The corresponding kinetic analysis revealed Δ*G*^‡^ = 13.0 kcal mol^−1^ in [D_8_]THF and Δ*G*^‡^ = 13.2 kcal mol^−1^ in CD_3_OD for this process (Supplementary Figs. 49 and 50), which is in stark contrast to the theoretically computed barrier for the thermal conversion of **E-1** to **A-1**. As a result of these experimental findings and discrepancies with calculations, it became clear that this fourth isomer could not be **E-1**. However, remarkably good agreement between the theoretically calculated and experimental NMR spectra was found for the epoxide constitutional isomer **D-1**. Also, the calculated Δ*G*^‡^ for the thermal conversion of **D-1** to **E-1** matches very well with the value established experimentally for the decay of the intermediate.

**Fig. 5:**
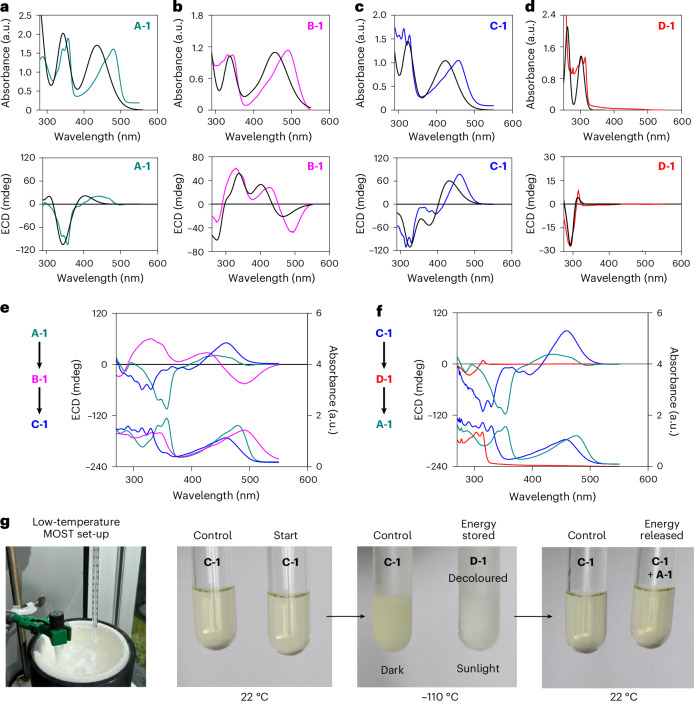
UV–visible and ECD spectroscopic analyses of molecular motor 1 and application in MOST. **a**–**d**, Experimental (coloured lines) and calculated (CAM-B3LYP/6-311G(d,p)/PCM(Et_2_O), black lines) UV–visible (top) and ECD (bottom) spectra are shown for the isomers of the (*R*)-configured motor **1**: **A-1** (**a**), **B-1** (**b**), **C-1** (**c**) and **D-1** (**d**). The experimental spectra were measured at low temperature in diethyl ether–isopentane–ethanol (5:5:2). **e**,**f**, Experimental ECD (top) and UV–visible (bottom) spectra for sequential **A-1** → **B-1** → **C-1** (**e**) and **C-1** → **D-1** → **A-1** (**f**) conversions. Proper motor behaviour is evidenced by the selective sequential isomer interconversions. **g**, Low-temperature MOST application for **C-1**, showing the utility of the motor for solar energy capture. Source data

To support the tentative isomer assignment of the intermediate as **D-1**, as well as the assignments of all the other isomers, the DFT-calculated NMR, UV–visible and ECD spectra (Supplementary Sections 4.1–4.3) were compared with the experimental spectra (Fig. 5 and Supplementary Figs. 58–67). We found that the theoretically predicted ^1^H NMR chemical shifts are in very good agreement with the experimental values, based on the assumption that the photoproduct of **C-1** is indeed **D-1** (Supplementary Figs. 5 and 6).

The UV–visible and ECD spectra of **A-1**, **B-1**, **C-1** and **D-1** recorded at low temperature were compared with calculated spectra and again very good agreement was found (Fig. 5a–d). To this end, Et_2_O–isopentane–EtOH (5:5:2) was used as solvent to access very low temperatures, below −130 °C, while still matching the intermediate polarity of the initially used THF-based solutions. This allowed us to obtain UV–visible and ECD spectra of the pure **D-1** isomer at −120 °C and even spectra of the pure fleeting **B-1** isomer at −160 °C.

The UV–visible spectrum recorded after irradiation of enantiomerically pure **A-1** at −160 °C displayed a bathochromic shift, while the ECD spectrum showed an inverted Cotton effect (Fig. 5e). This behaviour reveals that in the direct photoproduct of **A-1** the conjugation of the central double bond is intact and that this intermediate possesses opposite helicity. Furthermore, the bathochromic shift indicates a change from (*E*) to (*Z*) configuration, similar to typical HTI photoswitches. This is supported by theory, which predicts the S_1_ state (ππ* excitation; Supplementary Fig. 10) of **A-1** (3.11 eV) to be 0.14 eV higher in energy than that of **B-1** (2.97 eV).

Very good matches with the calculated UV–visible and ECD spectra of **B-1** can be observed, and thus the photoproduct of **A-1** can confidently be assigned to this isomer (Fig. 5b). Thermal annealing at −108 °C led to the known spectra of **C-1** (Fig. 5e), directly reporting the full unidirectionality of the **A-1** → **B-1** → **C-1** conversion sequence. The observed blueshift in the ECD and absorption spectra, along with the decrease in absorption intensity when comparing **A-1** and **C-1**, is further corroborated by TDDFT calculations (S_1_ for **C-1** at 3.21 eV (oscillator strength *f* = 0.272) and for **A-1** at 3.11 eV (*f* = 0.297)).

The irradiation of enantiomerically pure **C-1** at −120 °C revealed very distinct behaviour (Fig. 5f). A new intermediate state formed with a strongly hypsochromically shifted absorption. No absorption in the visible range remained after full conversion in the photostationary state, directly evidencing the rupture of the central double bond and loss of conjugation in the intermediate. In addition to the UV–visible absorption, the corresponding ECD spectrum also matched very well the theoretically predicted spectrum of intermediate **D-1** (Fig. 5d), which allowed us to unequivocally assign this isomer. Thus, a highly unusual constitutional alteration takes place in the photochemical transition of **C-1** to **D-1**, following a pathway that prevents rapid energy loss, aligning well with theoretical predictions (Fig. 3). Thermal annealing at −80 °C led to full conversion to **A-1**, as evidenced by the changes in the UV–visible and ECD spectra without the observable formation of **E-1** (Fig. 5f). This behaviour is expected from the very small calculated Δ*G*^‡^ for the THI leading from **E-1** to **A-1**. Therefore, **D-1** seems to convert directly to **A-1** at the temperature of the experiment, but in fact first undergoes epoxide ring opening and reverse proton transfer to **E-1** and then a subsequent (indiscernible) quick THI to **A-1**.

Overall, these combined NMR, UV–visible and ECD experiments allowed for unambiguous isomer assignment, confirming constitutional alteration with proton transfer as key steps. Complete unidirectionality is present even in protic solvents such as CD_3_OD under deuterium exchange (Fig. 4b and Supplementary Fig. 50). When comparing the kinetics for the transition from **D-1** to **A-1** (both intramolecularly hydrogen bonded) in [D_8_]THF or CD_3_OD, roughly the same Δ*G*^‡^ = 13 kcal mol^−1^ was found (Supplementary Figs. 50 and 51). For this process, full disruption of the hydrogen bond is not needed, rather proton hopping during epoxide opening. Therefore, solvent effects possibly cancel out rather than prefer one isomeric state and significantly alter the energy landscape.

### MOST application

For possible applications of this molecular motor, it needs to be emphasized that a significant redshift of about 50 nm is observed in the absorptions of the stable **A-1** and **C-1** isomers compared with the original HTI motor with a sulfoxide stereogenic centre^23^. Such a redshift is desirable for many applications of molecular motors, for example, in the context of biology, catalysis or materials, and thus makes motor **1** a highly interesting candidate in this regard. It also improves absorption overlap with the solar spectrum, enabling sunlight to power this motor. Furthermore, the favourable combination of isomer **D-1**’s very high energy content with its colourless UV absorption and almost quantitative enrichment under irradiation makes motor **1** a very attractive candidate for low-temperature applications^58^ in MOST systems^59,60^. For MOST, discolouration of the material on sunlight irradiation is desired to guarantee full light penetration and thus full conversion. Consequently, we tested whether a cooled solution of motor **1** could be converted to the colourless state upon sunlight irradiation (Fig. 5g and Supplementary Figs. 68 and 69). Starting from isomer **C-1**, direct sunlight irradiation at −110 °C led to full conversion of the initially yellow–orange isomer to a colourless solution. After warming to ambient temperature, recolouring took place and UV–visible analysis showed high conversion to **A-1**. Thus, a significant amount of the incident sunlight was stored in the colourless high-energy state **D-1** (Fig. 5g and Supplementary Fig. 70), which can be easily tracked by visible changes in colour.

## Conclusion

We have described here a light-driven molecular motor **1** possessing an intramolecular hydrogen bond. In contrast to all earlier HTI motor set-ups, motor **1** receives its asymmetry from a carbon-based stereogenic centre. We have demonstrated that this asymmetry is effectively translated into complete unidirectionality of the motor rotation. A unique and distinct operation mechanism has been established in which constitutional alteration and proton transfer processes allow a very large amount of the provided light energy to be stored within the motor rotation cycle. With this molecular set-up, a unique class of molecular motor has become available that can be supercharged by direct sunlight irradiation. The utility of this feature is evidenced by employing the motor in low-temperature MOST, opening unexplored avenues for molecular motor applications in solar energy conversion and batteries. Furthermore, this development will be of particular benefit for applications where a significant energy budget or workload is expended, for example, in active mechanically driven processes^61–66^ or bulk material changes^67–71^, or where supramolecular integration via, for example, hydrogen bonding^42,72^ leads to more complex molecular machine capacities.

## Methods

Reagents and solvents were obtained from abcr, Acros Organics, Merck, Sigma-Aldrich or TCI in the qualities puriss., p.a. or purum and used as received. Technical solvents were further distilled on a rotary evaporator (Heidolph Hei-VAP) before use in column chromatography and extraction. Anhydrous solvents purchased from Merck, Sigma-Aldrich and Acros were used without further purification. Reaction progress was monitored by thin-layer chromatography using SiO_2_-coated aluminium plates (Merck 60, F-254) with detection by UV light irradiation (254 or 366 nm) to determine retardation factors.

Flash column chromatography was performed using silica gel 60 *(*Merck, particle size 0.063–0.200 mm, or Macherey-Nagel, particle size 0.04–0.063 mm).

High-performance liquid chromatography (HPLC) was performed using a Shimadzu HPLC system comprising an LC-20AP solvent delivery module, a CTO-20A column oven, an SPD-M20A photodiode array UV–visible detector and a CBM-20A system controller with a semi-preparative CHIRALPAK IC (functionalized cellulose) or ID (functionalized amylose) column (particle size 5 µm) from Daicel and HPLC-grade solvents from Sigma-Aldrich, Honeywell, VWR and ROTH.

^1^H and ^13^C NMR spectra were recorded on a Bruker Avance III HD 400 (400 MHz), Bruker Avance Neo HD 400 MHz, Bruker Avance Neo HDX (500 MHz) or Bruker Avance Neo HDX (600 MHz) spectrometer with a DCH-Z^13^C/^1^H cryoprobe. Deuterated solvents were obtained from Cambridge Isotope Laboratories, Eurisotop, Deutero or Sigma-Aldrich. ^1^H and ^13^C NMR chemical shifts (*δ*) are reported in parts per million (ppm) relative to residual solvent signals, which were used as internal reference. For ^1^H NMR: *δ*(CDCl_3_) = 7.26 ppm, *δ*(CD_2_Cl_2_) = 5.32 ppm, *δ*([D_6_]DMSO) = 2.50 ppm and *δ*([D_8_]THF) = 3.58 ppm (DMSO represents dimethylsulfoxide). For ^13^C NMR: *δ*(CDCl_3_) = 77.16 ppm, *δ*(CD_2_Cl_2_) = 54.00 ppm, *δ*([D_6_]DMSO) = 39.52 ppm and *δ*([D_8_]THF) = 67.6 ppm. Resonance multiplicity is indicated as s (singlet), d (doublet), t (triplet), q (quartet), sept (septet) and m (multiplet); coupling constants (*J*) are given in hertz (Hz).

Electron Impact mass spectra were recorded on a Thermo Q Exactive GC Orbitrap or Finnigan MAT 95 mass spectrometer.

Infrared spectra were recorded on a Perkin Elmer Spectrum BX-FT-IR spectrometer equipped with a Smiths Detection DuraSamplIR II ATR (attenuated total reflectance) device. Transmittance is qualitatively indicated in wavenumbers (cm^−1^) and intensities as strong (s), medium (m) and weak (w).

UV–visible spectra were recorded on Varian Cary 5000 and Chirascan V100 spectrophotometers in a quartz cuvettes (1-cm path length). Spectral-grade solvents were obtained from VWR. Absorption wavelengths (*λ*, in nm) and molar extinction coefficients (*ε*, in l mol^−1^ cm^−1^) are reported.

ECD spectra were recorded on a Chirascan V100 spectrometer. For low-temperature measurements, the samples were analysed in quartz cuvettes placed inside an Oxford Optistat DN 1704 cryostat with an Oxford ITC-4 temperature controller. Liquid nitrogen was used as the cryogen. A steady flow of nitrogen gas was established to minimize the condensation of water inside the sample chamber.

Photoisomerization experiments were conducted either in NMR tubes in different deuterated solvents ([D_8_]THF, CD_2_Cl_2_–CS_2_ and [D_8_]THF–CS_2_ mixtures) or in quartz cuvettes (1 cm) in Et_2_O–isopentane–EtOH (5:5:2, v/v/v). Irradiations were performed using a Prizmatix UHP light-emitting diode (450 nm) for ECD and coupled to a 1,500 µm quartz fibre for in situ NMR experiments.

Melting points were measured on a Büchi B-540 melting point apparatus in open capillaries.

X-ray diffraction analysis was performed on a SuperNova Atlas diffractometer using Cu Kα radiation.

### Synthesis of motor 1

Details of the synthetic procedures and characterization of the obtained compounds are provided in Supplementary Sections 5 and 6.

The synthesis started from commercially available 3-(2,5-dimethoxyphenyl)propanoic acid (**3**). Following a literature protocol, 4,7-dimethoxyindanone (**4**) was obtained after intramolecular Friedel–Crafts acylation^23^. The isopropyl group was introduced via α-deprotonation with NaH at 0 °C and subsequent substitution with isopropyl iodide in THF. After stirring at 23 °C for 20 h, the resulting indanone **5** was obtained in 31% yield. To our surprise, prolonged treatment of **5** with base led to the formation of the α-hydroxyindanone **6**. A similar reaction was reported during the writing of this manuscript by Crespi, Feringa and co-workers under Lewis acid conditions^44^, which we were not aware of at the time we synthesized our motor. After optimization, a satisfactory yield of 60% was achieved when bubbling air through the solution to increase the level of dioxygen reactant. A closer examination of this reaction in the future will reveal details about the mechanism and probe its scope; however, it is already evident that the hydroxy group stems from the dioxygen reactant. A related synthetic transformation and mechanism have previously been described using an amine as base to introduce α-hydroxy groups into ketones^73^. When applying these reaction conditions using 1,5,7-triazabicyclo[4.4.0]dec-5-ene as base in DMSO and maximizing the dioxygen content of the reaction vessel by bubbling oxygen through the reaction mixture, a further increase in the hydroxylation yield to 90% was possible.

Benzothiophenone **7** was prepared from commercially available 2-bromothiophenol and bromoacetic acid in two steps according to a literature procedure^74^. The condensation of indanone **6** and benzothiophenone **7** in CH_2_Cl_2_ was promoted by the addition of BCl_3_ as Lewis acid at −78 °C to give the final motor **1** in 23% yield.

The metastable isomer **C-1** was isolated after irradiation of **A-1** with 450 nm light and purification by flash column chromatography.

### Theoretical analysis

NMR spectra were calculated at the CAM-B3LYP/6-311G(d,p)(PCM) level of theory (Supplementary Figs. 5 and 6 and Supplementary Table 3). Non-covalent interactions were probed using molecular electrostatic potential maps and reduced density gradients based on DFT calculations (Supplementary Figs. 7–9 and Supplementary Section 3.5). UV–visible spectra report significant electronic changes, such as the (*E*) or (*Z*) configuration of the central double bond and especially its rupture during the constitutional alteration step, while ECD spectra are highly sensitive to the particular helicity of the isomers, which would complete an unambiguous assignment of spectral species experimentally. Therefore, we calculated the absorption and ECD spectra by TDDFT (Supplementary Table 5 and Supplementary Figs. 10–12) as well as at the CASSCF level of theory (see Supplementary Table 6 and Supplementary Figs. 13 and 14 for absorption properties and active spaces). Computational results, for example, for the vertical excitation energies, oscillator strengths and charge density differences of the respective electronic transitions are summarized in Supplementary Sections 4.1 and 4.3.

### Low-temperature UV–visible and ECD spectra

Experimental spectra were recorded in Et_2_O–isopentane–EtOH (5:5:2) at low temperatures and theoretical spectra were calculated at the CAM-B3LYP/6-311G(d,p)/PCM(Et_2_O) level of theory. The ECD spectra shown in Fig. 5 are of the (*R*)-configured isomers of motor **1**. The theoretical spectra are redshifted by 30–40 nm and Gaussian broadenings of *σ* = 0.2 or 0.3 eV were applied. The intensities of the simulated spectra are scaled to the experimental spectra. Regarding the ECD experiments, only spectra of (*R*)-configured isomers are shown for reasons of clarity and consistency. The recorded ECD spectra of (*S*)-configured isomers mirror the spectra of the (*R*)-configured isomers (Supplementary Sections 10.2 and 11).

## Online content

Any methods, additional references, Nature Portfolio reporting summaries, source data, extended data, supplementary information, acknowledgements, peer review information; details of author contributions and competing interests; and statements of data and code availability are available at 10.1038/s41557-026-02141-6.

## Supplementary information

Supplementary Information Supplementary Sections 1–14, Figs. 1–70 and Tables 1–8.

Supplementary Data 1 Crystallographic data for compound **A-1**, CCDC 2307157.

Supplementary Data 2 Crystallographic data for compound **C-1**, CCDC 2307158.

## Source data

Source Data Fig. 3 Statistical source data and coordinates of the calculated geometries in *xyz* format.

Source Data Fig. 4 Source data.

Source Data Fig. 5 Theoretical and experimentally obtained absorption and ECD spectra.

## Data Availability

Further relevant data supporting the findings of this study are available in the Article, its Supplementary Information or upon request from the authors. *xyz* coordinate files of the ground-state minima, transition states and conical intersection CI_10_ of motor **1**, as well as the ground-state minima and transition states of motor **2**, are available via Zenodo at 10.5281/zenodo.19086195 (ref. ^75^). The X-ray crystallographic coordinates for the structures reported in this study have been deposited at the Cambridge Crystallographic Data Centre (CCDC), under deposition numbers 2307157 (**A****-1**) and 2307158 (**C****-1**). These data can be obtained free of charge from The Cambridge Crystallographic Data Centre via www.ccdc.cam.ac.uk/data_request/cif. Source data are provided with this paper.
