# Supplementary material for: A supercharged molecular motor operating by constitutional alteration and proton transfer
Source: Nat Chem. 2026 Jun 3;18(7):1186–94. doi: 10.1038/s41557-026-02141-6 (PMC13322973; doi:10.1038/s41557-026-02141-6)
Supplement: Supplementary file 1 — Supplementary Sections 1–14, Figs. 1–70 and Tables 1–8. [file 41557_2026_2141_MOESM1_ESM.pdf]

nature chemistry

Supplementary information https://doi.org/10.1038/s41557-026-02141-6

A supercharged molecular motor operating by constitutional alteration and proton transfer

In the format provided by the authors and unedited

Table of Contents

1. Materials and Methods 3

2. Conformational Analysis of Motor States 5

3. Theoretical Description of the Ground State of Motor 1 6

   3.1 Screening of Ground State Energy Profiles for Compound 1 7

   3.2 Calculations on Rotamers of Epoxide D-1 8

   3.3 NMR Spectra Simulations of Motor 1 11

   3.4 Methoxy-Substituted Motor 2 15

   3.5 Analysis of Non-Covalent Interactions in Motor 1 16

   Molecular Electrostatic Potential Maps 16

   Non-Covalent Interactions and Reduced Density Gradient Analysis 17

   Binding Energy Calculations 20

4. Theoretical Description of the Excited State of Motor 1 21

   4.1 Excitation analysis based on time-dependent density functional theory 23

   4.2 Comparison of experimental and theoretical UV/vis and ECD Spectra 24

   4.3 Multiconfigurational excitation analysis and active spaces 26

   4.4 Conical intersections optimizations 29

   4.5 Minimum energy path calculations 31

5. Syntheses of Compounds 32

   Synthesis of HTI 1 32

   4,7-dimethoxy-2,3-dihydro-1*H*-inden-1-one (4)[35] 33

   2-*iso*propyl-4,7-dimethoxy-2,3-dihydro-1*H*-inden-1-one (5) 33

   2-hydroxy-2-*iso*propyl-4,7-dimethoxy-2,3-dihydro-1*H*-inden-1-one (6) 34

   2-((2-bromophenyl)thio)acetic acid (8)[34] 36

   7-bromobenzo[b]thiophen-3(2H)-one (7)[34] 36

   (*E*/*Z*)-7-bromo-2-(2-hydroxy-2-*iso*propyl-4,7-dimethoxy-2,3-dihydro-1*H*-inden-1-ylidene)benzo[*b*]thiophen-3(2*H*)-one ((*E*/*Z*)-1) 37

6. NMR Spectra of Synthesized Compounds 40

7. Enantiomeric Resolution 51

8. Temperature and Irradiation-Dependent Behavior of Motor 52

8.1 Elevated Temperature Behavior ... 52

9. *In situ* NMR Irradiation Experiments at Low Temperature ... 59

9.1 Motor Function and Thermal Behavior of HTI-1 ... 59

10. Photophysical Properties of Motor 1 ... 73

10.1 UV/vis Spectra ... 73

10.2 ECD and UV/Vis Measurements ... 74

11. Low-Temperature ECD Irradiations and Thermal Annealing ... 76

12. Experimental Data on Sunlight-Driven Molecular Solar Thermal Energy Storage by Supercharged D-1 ... 80

13. Crystal Structure Data ... 83

14. References ... 84

1. Materials and Methods

Reagents and solvents were obtained from abcr, Acros Organics, Merck, Sigma-Aldrich or TCI in the qualities puriss., *p.a.*, or *purum* and used as received. Technical solvents were further distilled on a rotary evaporator (Heidolph Hei-VAP) before use for column chromatography and extraction. Anhydrous solvents purchased from Merck, Sigma-Aldrich and Acros were used without further purification. Reaction progress was monitored by thin-layer chromatography (TLC) using aluminum plates coated with SiO_2 (Merck 60, F-254). Detection was done by irradiation with UV light (254 nm or 366 nm) in order to determine retardation factors (R_f).

Flash column chromatography was performed with silica gel 60 (Merck, particle size 0.063-0.200 mm; or Macherey-Nagel, particle size 0.04-0.063 mm).

High Performance Liquid Chromatography (HPLC) was performed on a Shimadzu HPLC system consisting of a LC-20AP solvent delivery module, a CTO-20A column oven, a SPD-M20A photodiode array UV/Vis detector, and a CBM-20A system controller using a semi-preparative CHIRALPAK® IC or ID column (particle size 5 µm) from Daicel and HPLC grade solvents from Sigma-Aldrich, Honeywell, VWR, and ROTH.

^{1}H and ^{13}C NMR spectra were measured on a Bruker Avance III HD 400 (400 MHz), Bruker Avance Neo HD 400 MHz, Bruker Avance Neo HDX 500 MHz, or Bruker Avance Neo HDX 600 MHz spectrometer with cryo probe DCH-Z^{13}C/^{1}H. Chemical shifts (δ) are reported relative to residual solvent signals in the ^{1}H and ^{13}C NMR spectra, which were used as internal reference. Deuterated solvents were obtained from Cambridge Isotope Laboratories or Eurisotop, Deutero GmbH, and Sigma-Aldrich. For ^{1}H NMR: $CDCl_3$ = 7.26 ppm, CD_2Cl_2 = 5.32 ppm, DMSO-d_6 = 2.50 ppm, and THF d_8 = 3.58 ppm. For ^{13}C NMR: $CDCl_3$ = 77.16 ppm, CD_2Cl_2 = 54.00 ppm, DMSO-d_6 = 39.52 ppm, and THF d_8 = 67.6 ppm. Resonance multiplicity is indicated as s (singlet), d (doublet), t (triplet), q (quartet), sept (septet) and m (multiplet). Chemical shifts are given in parts per million (ppm). Coupling constant values (J) are given in Hertz (Hz).

Electron Impact (EI) mass spectra were measured on a Thermo Q Exactive GC Orbitrap or Finnigan MAT 95 mass spectrometer.

Infrared spectra were recorded on a Perkin Elmer Spectrum BX-FT-IR spectrometer equipped with a Smiths Detection DuraSamplIR II ATR-device. Transmittance values are qualitatively described by wavenumber (cm^{-1}) as strong (s), medium (m), and weak (w).

UV/Vis spectra were measured on a Varian Cary 5000 and a Chirascan V100 spectrophotometer. The spectra were recorded in a quartz cuvette (1 cm path length). Spectral grade solvents were obtained from VWR. Absorption wavelengths (λ) are reported in nm and the molar extinction coefficients (ε) in L mol^{-1} cm^{-1}.

Electronic circular dichroism (ECD) spectra were measured on a Chirascan V100. For low temperature measurements, the samples were measured in quartz cuvettes and were placed inside an Oxford Optistat DN 1704 cryostat with Oxford ITC-4 temperature controller. Liquid nitrogen was used as cryogen. A steady flow of nitrogen gas was established in order to minimize condensation of water inside the sample chamber.

Photoisomerization experiments were conducted either in NMR tubes in different deuterated solvents (THF-d_8, CD_2Cl_2:CS_2 and THF-d_8:CS_2 mixtures) or in quartz cuvettes (1 cm) in EPA (Et_2O:*i*-pentane:EtOH mixture, 5:5:2 v/v). Irradiations were performed using a Prizmatix UHP LED (450 nm) for ECD and coupled to a 1500 µm quartz fiber for *in situ* NMR experiments.

Melting points (mp) were measured on a Büchi B-540 melting point apparatus in open capillaries.

Quantum chemical calculations were performed with the software package Gaussian 16 Rev. C.01[1] Gaussian 09 Rev. A.02,[2] ORCA 5.0.3,[3,4] **GAMESS 2023 R1,**[5] and OpenQP.[6,7,8,9] Non-covalent interaction analysis was performed using the Multiwfn-package, version 3.8.[10] Structures and non-covalent interactions were visualized using VMD 1.9.3.[11]

X-ray diffraction crystallographic analysis was performed on a SuperNova Atlas diffractometer using Cu-Kα-radiation.

2. Conformational Analysis of Motor States

Motor **1** adapts five different intermediates **A**, **B**, **C**, **D**, and **E** during the rotation, each for a given (*R*) or (*S*) configuration of the carbon stereocenter.

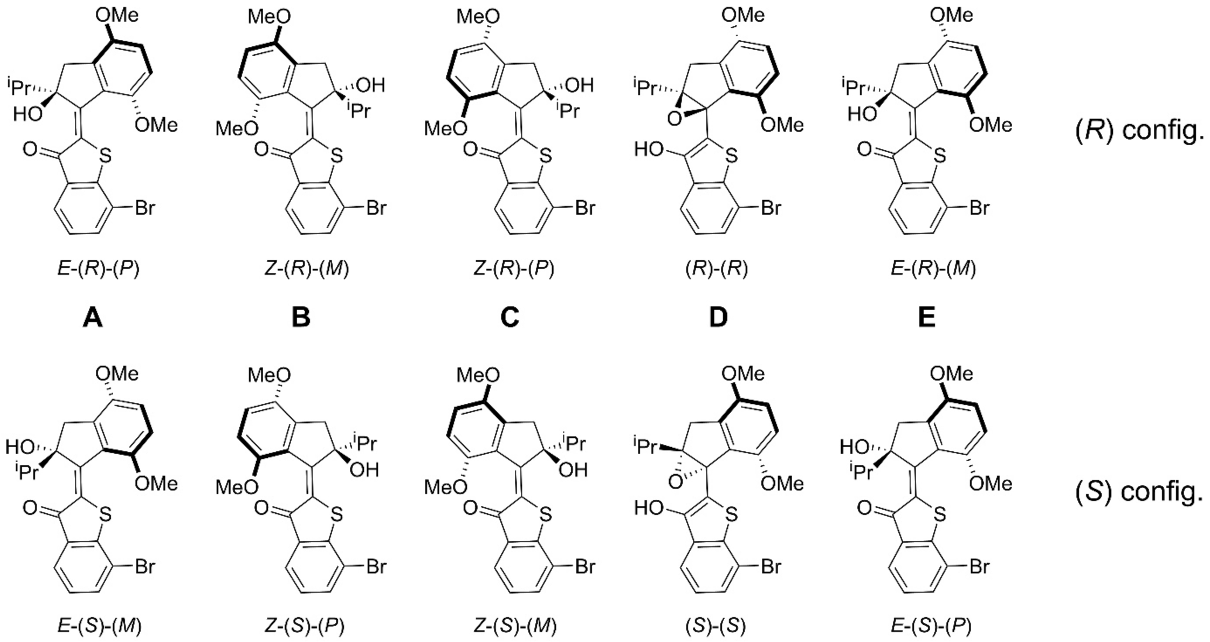

Supplementary Figure 1. Absolute configuration of all possible stereoisomers **A**, **B**, **C**, **D**, and **E** for motor **1**. Stable and metastable designations for isomers are given for ambient temperatures.

3. Theoretical Description of the Ground State of Motor 1

Quantum chemical calculations were performed using **Gaussian 16**[1] and **ORCA 5.0.3**[3,4] to characterize the ground state properties of the studied systems.

The equilibrium geometries of **A-1, B-1, C-1, D-1, and E-1** were optimized in their singlet ground state using **density functional theory (DFT)**. Calculations were carried out with the hybrid functionals **B3LYP**,[12] **CAM-B3LYP**,[13] **wB97xD**,[14] and **wB97X-3c**,[15] employing the **6-311G(d,p)**[16,17] basis set for all elements. Solvent effects were included using the **electron density variant of IEFPCM**[18,19,20] **(THF, dielectric constant = 4.24)**. A vibrational frequency analysis confirmed that the optimized geometries correspond to true minima on the potential energy surface.

NMR shielding tensors were calculated at the **CAM-B3LYP/6-311G(d,p)** level, with solvent effects modelled via **IEFPCM (THF)**. Chemical shifts (δ) were referenced to **TMS**, averaging all magnetically equivalent hydrogens.

Based on the most stable isomers of **A-1, B-1, C-1, D-1,** and **E-1**, transition state geometries were optimized for the following key conversions: **TS_{BC}-1** (**B-1** → **C-1**), **TS_{DE}-1** (**D-1** → **E-1**), and **TS_{EA}-1** (**E-1** → **A-1**). Moreover, a transition state for the rotation of **E-1** around its former double bond was optimized as well as a transition state for the thermal backreaction of **D-1** to **C-1** to access the barriers for these processes. Vibrational frequency calculations confirming their nature as first-order saddle points (**one imaginary frequency**).

3.1 Screening of Ground State Energy Profiles for Compound 1

In order to reveal the influence of the level of theory on the energy landscape, different methods were first screened for the simple **A**-**B**-**C**-**E** rotation cycle for motor **1** and the results are summarized in Supplementary Table 1. While qualitative agreement was found among all employed levels of theory, CAM-B3LYP/6-311G(d,p)/PCM(THF) provided a barrier of +9.63 kcal/mol for the thermal helix inversion from **B-1** to **C-1**. This concurred well with the experimentally obtained barrier (+9.67 kcal/mol).

Supplementary Table 1. Ground-state energy profiles for all isomers of the motor **1** at different levels of theory. All calculations include implicit solvation with THF and relative Gibbs free energies are given with respect to the global minimum **A-1** in kcal/mol. *Single-point calculation based on the CAM-B3LYP optimizations and with use of extrapolation to the complete basis set limit.

Level of Theory	A-1	B-1	$\mathbf{TS_{BC}}$-1 ($\Delta G^{\ddagger}$)	C-1	E-1	$\mathbf{TS_{EA}}$-1 ($\Delta G^{\ddagger}$)
B3LYP/6-311G(d,p)	0.00	7.33	17.36 (+10.03)	3.60	1.95	3.95 (+2.00)
ωB97XD/6-311G(d,p)	0.00	7.22	17.94 (+10.72)	2.56	1.72	4.19 (+2.47)
CAM-B3LYP/6-311G(d,p)	0.00	8.09	17.72 (+9.63)	3.66	2.28	4.09 (+1.81)
ωB97XD/def2tzvp	0.00	7.00	18.45 (+11.45)	2.28	1.39	4.24 (+2.85)
ωB97X-3c	0.00	6.46	18.63 (+12.17)	1.86	2.47	4.29 (+1.82)
CCSD(T)/cc-pVQZ cbs*	0.00	6.52	17.07 (+10.55)	1.93	2.38	4.72 (+2.34)

Next, CAM-B3LYP and ωB97XD functionals were combined with 6-311G(d,p) to calculate the ground-state energy profile for the rotational cycle of motor **1** including the epoxide intermediate **D-1**. As can be seen from Supplementary Table 2, both levels of theory provide similar energy profiles and they are in good agreement with the experimental results especially for the barrier for **C-1** to **D-1** isomerization.

Supplementary Table 2. Gibbs free energies of different isomers and transition states computed at different levels of theory. Calculations include implicit solvation with THF. Relative values ΔG_0 are given with respect to the global minimum **A**.

Level of Theory	A-1	B-1	$\mathbf{TS_{BC}}$-1 ($\Delta G^{\ddagger}$)	C-1	$\mathbf{TS_{DC}}$-1 ($\Delta G^{\ddagger}$)	D-1	$\mathbf{TS_{DE}}$-1 ($\Delta G^{\ddagger}$)	E-1	$\mathbf{TS_{EA}}$-1 ($\Delta G^{\ddagger}$)
CAM-B3LYP/6-311G(d,p)	0.00	8.09	17.72 (+9.63)	3.66	40.90 (+28.90)	12.00	26.85 (+14.85)	2.28	4.09 (+1.81)
ωB97XD/6-311G(d,p)	0.00	7.22	17.94 (+10.72)	2.56		11.23	27.16 (+15.93)	1.72	4.19 (+2.47)

3.2 Calculations on Rotamers of Epoxide D-1

Scans around the former double bond in the epoxide structure revealed that the global minimum of the epoxide intermediate is in fact the hydrogen-bonded structure (rotamer **D-1**) which is formed in the photochemical step. Another local minimum exists (rotamer **D´-1**) which is not hydrogen bonded and 2.55 kcal/mol less favorable in energy (Supplementary Figure 2). Transition state energies for thermal rotation from **D-1** to **D-1'** rotation (19.5 kcal/mol) and for **D-1** to **C-1** back rotation (28.9 kcal/mol) were also obtained theoretically. In comparison, the **D-1** to **E-1** forward rotation encounters the lowest calculated barrier with 14.84 kcal/mol.

In order to investigate the effect of hydrogen bonding in the epoxide structures, both rotamers were optimized for a hypothetical derivative where the hydroxyl group is replaced by a methoxy group in the epoxide structure (rotamers **D-2** and **D´-2**). As depicted in Supplementary Figure 3, the results show that relative stabilities are now reversed. Without the possibility to hydrogen bond, rotamer **D´-2** is more favorable. It can therefore be concluded that there is a stabilizing effect of the hydrogen bond in the original epoxide **D-1** structure, from which thermal opening towards isomer **E-1** is possible.

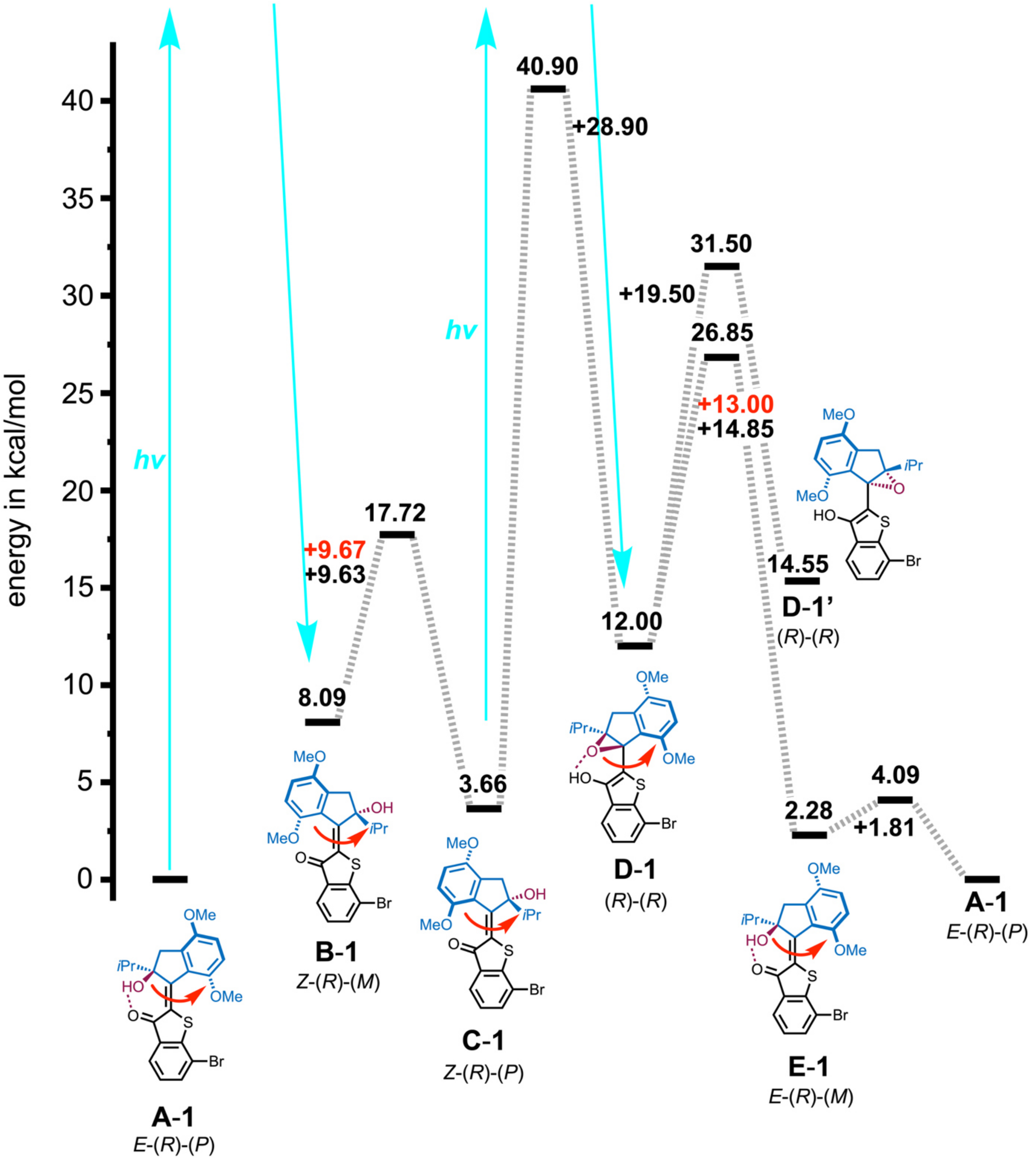

Supplementary Figure 2. Full ground state energy profile of Motor **1** including hydrogen-bonded **D** and non-hydrogen-bonded **D′** rotamers as well as transition state energies for thermal **D-1** to **D-1’** rotation (19.5 kcal/mol) and for **D-1** to **C-1** back rotation (28.9 kcal/mol).

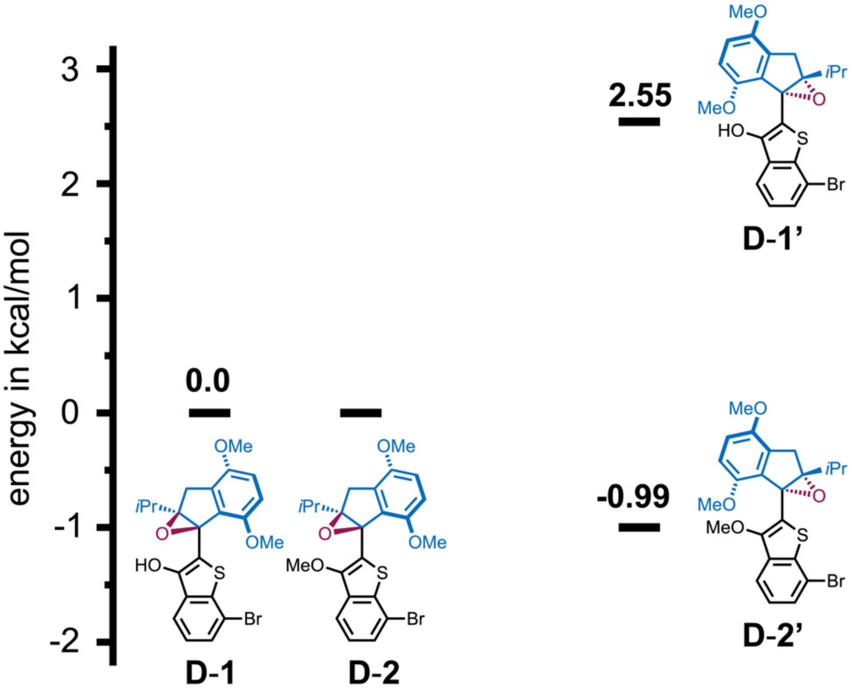

Supplementary Figure 3. Relative stabilities of rotamers of motors **1** and **2**. Hydrogen bonding leads to stabilization of rotamer **D**. Rotamer **D** is set to zero for both sets of structures and the energy of rotamer **D´** is given with respect to the corresponding rotamer **D**.

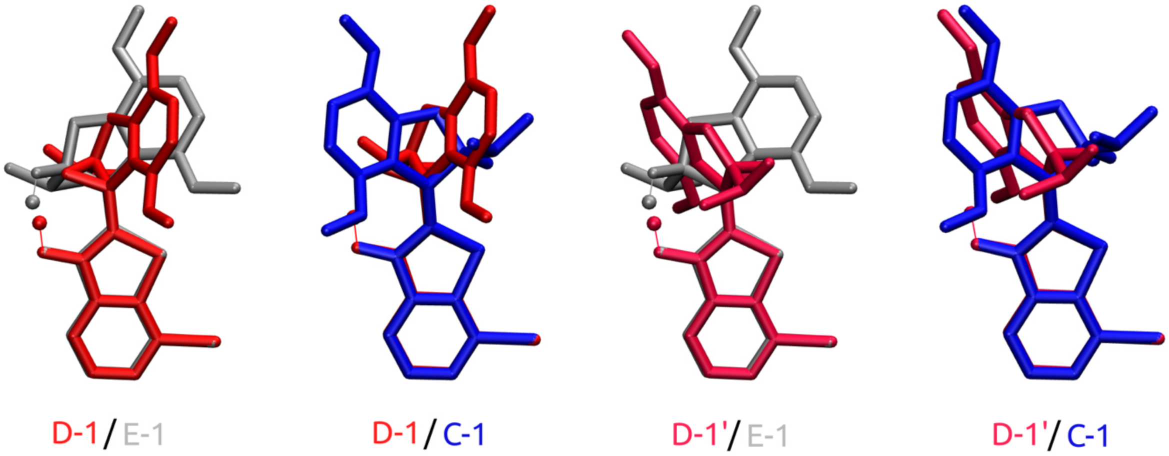

Supplementary Figure 4. Structural overlays of **D-1** with **E-1** and **C-1** as well as of **D'-1** with **E-1** and **C-1**. The **D-1** structure is rotated significantly towards **E-1**, while the **D'-1** structure resembles much more closely **C-1**.

3.3 NMR Spectra Simulations of Motor 1

For the simulation of ^{1}H and ^{13}C NMR spectra of **D** isomer of motor **1**, shielding calculations were performed at the same CAM-B3LYP/6-311G(d,p)/PCM(THF) level of theory as for the previous optimizations and subsequent frequency analyses. After optimizing and calculating the frequencies of TMS as reference using the same DFT method, chemical shifts (δ) were computed by averaging all magnetically equivalent hydrogens. The corresponding results are depicted in Supplementary Table 3, as well as Supplementary Figures 5 and 6.

Supplementary Table 3. Comparison of experimental and theoretical ^{1}H and ^{13}C NMR chemical shifts (δ) of epoxide **D-1** given in ppm. Experimental spectra are recorded in THF-d_8 at −120 °C and theoretical calculations were performed at the CAM-B3LYP/6-311G(d,p)/PCM(THF) level of theory. For ^{13}C chemical shifts, only characteristic carbons are depicted.

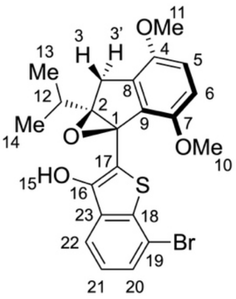

Proton	Experimental	Theoretical	Carbon	Experimental	Theoretical
3<br>3′	2.90 and/or 3.13	2.98<br>3.11	1	79.7 or 71.3	77.2
5	6.95 or 6.86	6.96	2	79.7 or 71.3	84.2
6	6.95 or 6.86	6.83	3	29.3	32.8
10	3.80 or 3.60	3.88	12	30.7	34.5
11	3.80 or 3.60	3.65	13<br>14	19.3 and/or 18.9	19.9<br>19.7
12	1.55-1.48	1.51	16	147.0	156.7
13 (same side to epoxide)<br>14 (opposite to epoxide)	1.18 and/or 0.95	1.24<br>0.98	17	109.9	118.2
15	8.84	8.31	4<br>7	152.4 or 151.0	159.4<br>161.3
20	7.77	7.74	8<br>9	133.2 or 129.8	143.2<br>137.3
21	7.40	7.69	18	138.1	151.4
22	7.76	8.12	19	116.6	138.2
			23	135.5	142.4

	10	55.4 or 55.2	56.2
	11		57.0
	5	111.9 or 110.1	118.2
	6		115.4
	22	121.1	129.6
	21	126.5	133.5
	20	128.6	137.9

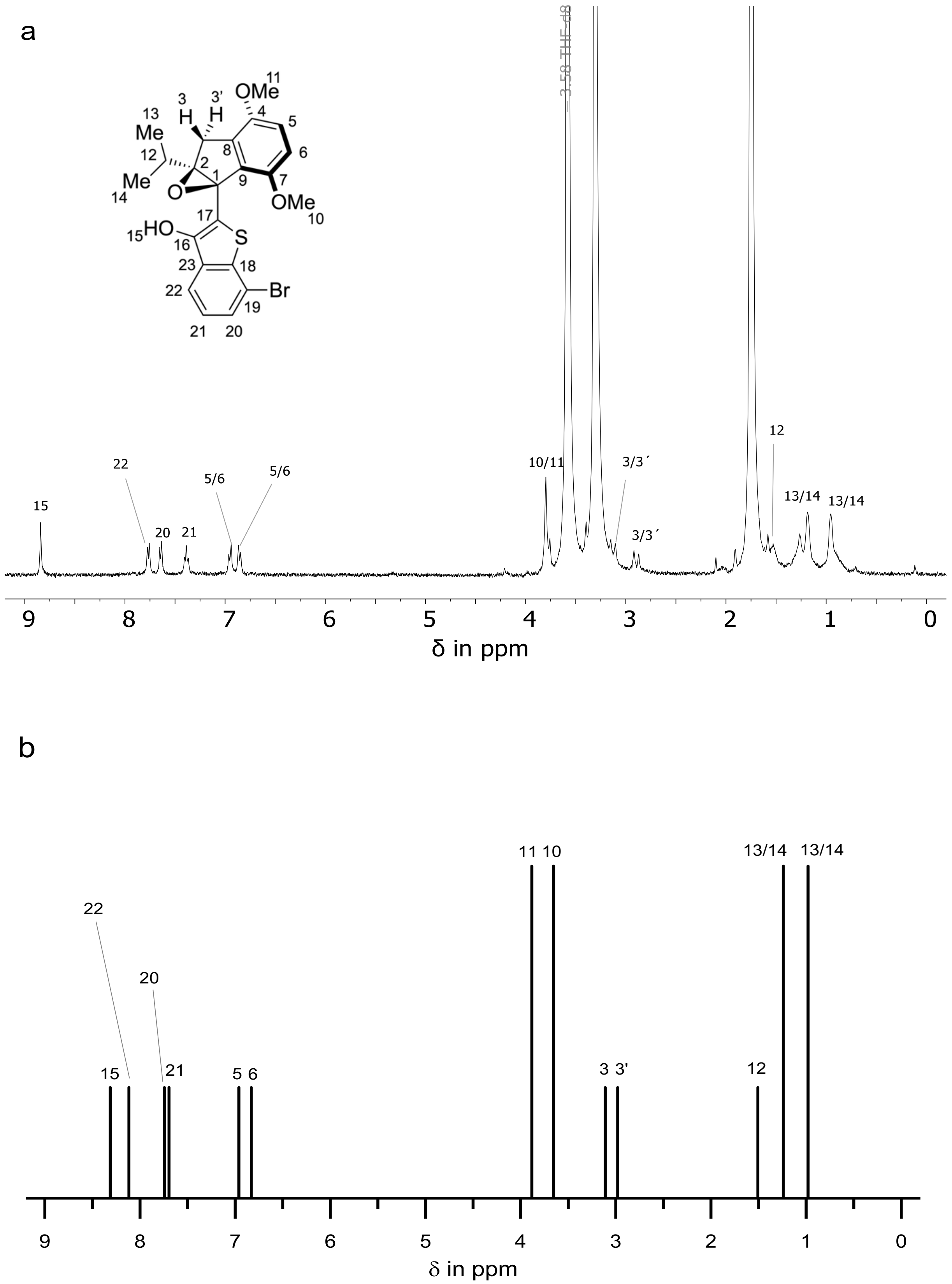

Supplementary Figure 5. Comparison of experimental and simulated ^{1}H NMR spectra of metastable isomer **D** of motor **1**. a) Experimental ^{1}H NMR spectrum of pure **D-1** (THF-d_8, 400 MHz, −120 °C). b) Theoretical ^{1}H NMR spectrum of **D-1** simulated at the CAM-B3LYP/6-311G(d,p)/PCM(THF) level of theory. Note that in the experimental ^{1}H NMR spectrum the remaining OCH_3 signal corresponding to H-C10 or H-C11 overlaps with the THF-d_8 reference solvent peak and it appears at 3.60 ppm as deduced from ^{1}H-^{13}C NMR HMBC experiment.

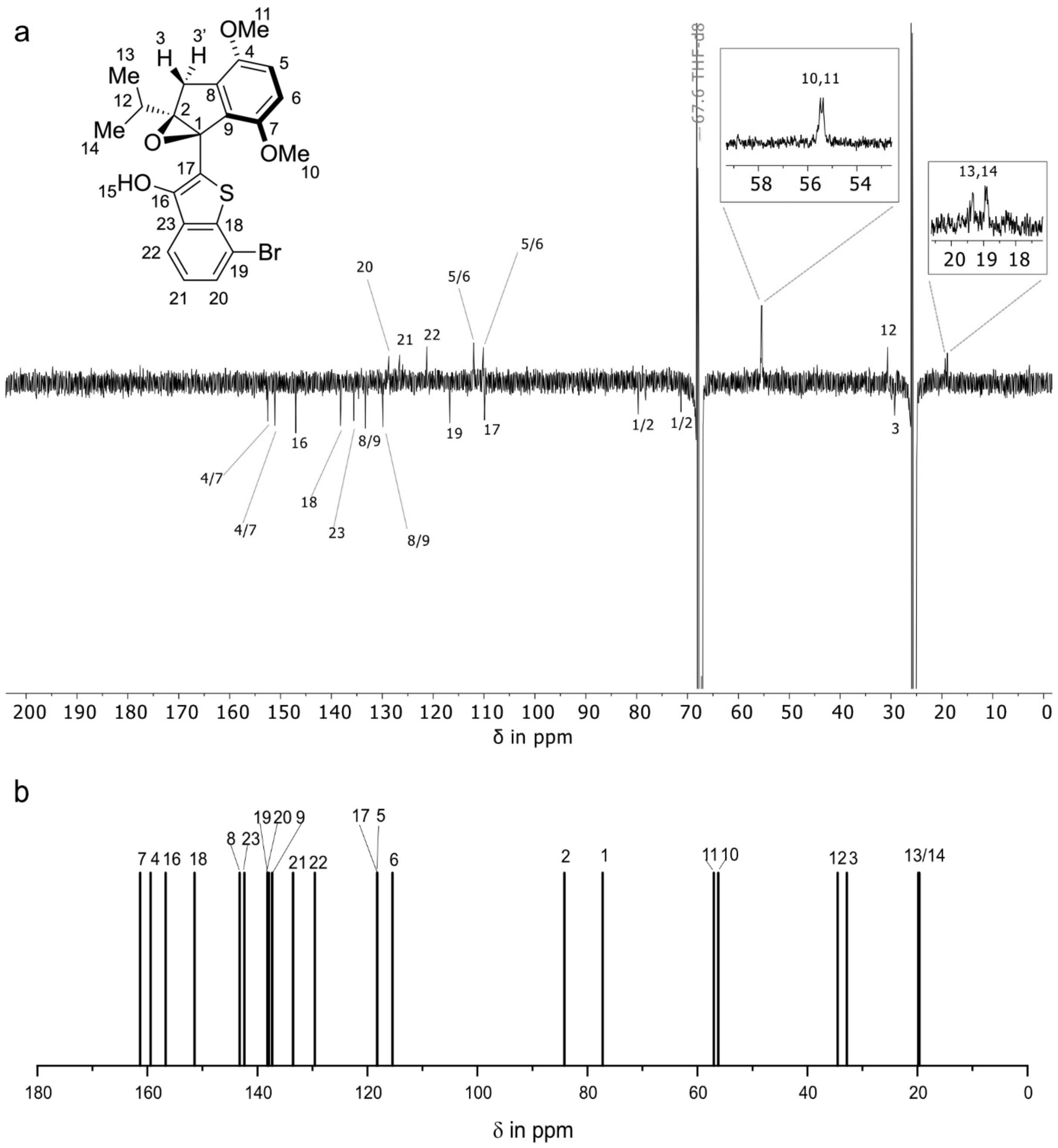

Supplementary Figure 6. Comparison of experimental and simulated ^{13}C NMR spectra of metastable isomer **D** of motor **1**. a) Experimental ^{13}C DEPTq NMR spectrum of 80% enriched sample of **D-1** (THF-*d*$_8$, 101 MHz, −120 °C). b) Theoretical ^{13}C NMR spectrum of **D-1** simulated at the CAM-B3LYP/6-311G(d,p)/PCM(THF) level of theory.

3.4 Methoxy-Substituted Motor 2

In order to investigate the effect of hydrogen bonding on the overall ground-state energy profile, the methoxy-substituted derivative **2** was calculated at the same CAM-B3LYP/6-311G(d,p)/PCM(THF) level of theory as the hydroxy-bearing motor **1**. The results show that in principle both motor setups are capable of rotating in the same direction (Supplementary Table 4). For methoxy-substituted motor **2**, **C** is the thermodynamically stable isomer instead of **A**. Energy differences between the isomers are significantly smaller than for the hydroxy motor **1**.

Supplementary Table 4. Calculated Gibbs energies in kcal/mol for the hydroxyl motor **1** and the methoxy-substituted derivative **2**, showing smaller energy differences between the isomers for the methoxy-derivative and reversed stabilities for **A** and **C** states.

**Compound**	**A**	**B**	**TS$_{BC}$**	**C**	**E**	**TS$_{EA}$**
HTI **1**	**0.00**	8.09	+9.63	3.66	2.28	+1.81
HTI **2**	1.35	3.13	+10.53	**0.00**	4.84	+3.06

3.5 Analysis of Non-Covalent Interactions in Motor 1

Molecular Electrostatic Potential Maps

Molecular electrostatic potential (MEP) maps serve as an important tool to rationalize the nature of both intra- and intermolecular interactions. Similarly, MEP maps have been widely used to explain trends observed in hydrogen bond donors and acceptors in a qualitative manner.[21] In general, the red color indicates high electron-density sites, blue color depicts low-electron-density sites, and green/yellow colors illustrates neutral sites in molecules. Therefore, the MEP maps of each relevant state for motor **1** were generated at the CAM-B3LYP/6-311G(d,p)/PCM(THF) level of theory with an isosurface value of 0.02. Supplementary Figure 7 shows that the MEP maps of **A-1**, **D-1** and **E-1** are characterized by significant electron-rich sites localized in O–H donor and C=O acceptor pairs involved in intramolecular hydrogen bonding. On the other hand, low-electron-density and/or neutral sites are observed for the molecular space of **B-1** and **C-1** including S atom and O–H moiety with no clear intramolecular hydrogen bonding formation.

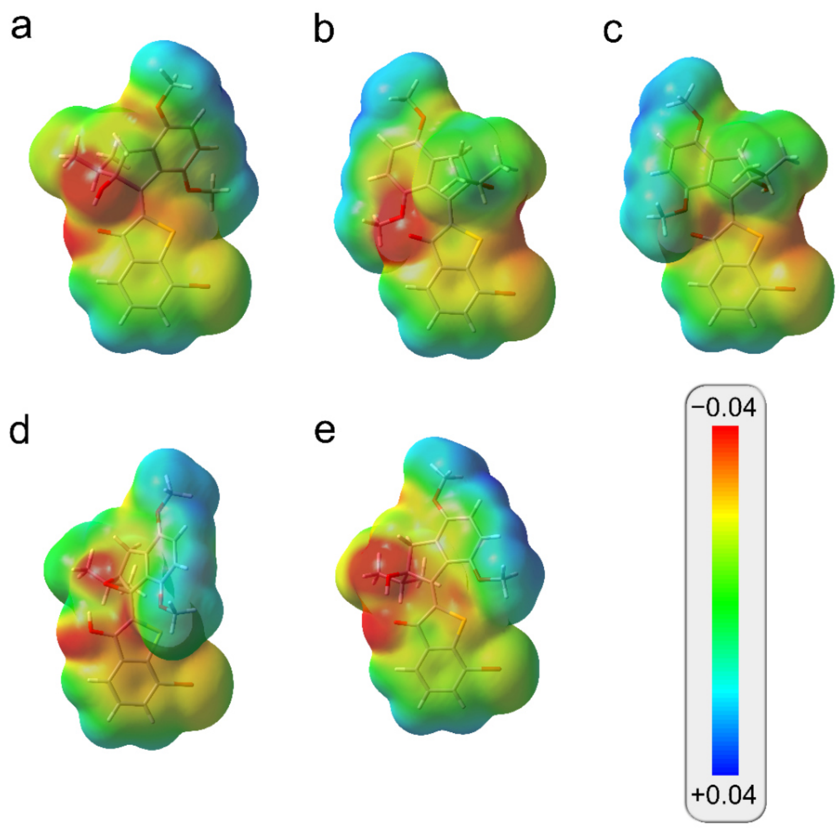

Supplementary Figure 7. Molecular electrostatic potential maps for isomers a) **A-1**, b) **B-1**, c) **C-1**, d) **D-1**, and e) **E-1** calculated at the CAM-B3LYP/6-311/PCM(THF) level of theory and shown with an isosurface value of 0.02.

Non-Covalent Interactions and Reduced Density Gradient Analysis

Non-covalent interaction (NCI) analysis[22] was performed using the *Multiwfn3.8* package.[10] The key quantity used in herein described method is the reduced density gradient (RDG) that is given by:

$$\mathrm{RDG} = \frac{1}{2(3\pi^2)^{\frac{1}{3}}} \frac{|\nabla\rho|}{\rho^{\frac{4}{3}}} \tag{4}$$

where ρ stands for the electron density. Regions of non-covalent interactions are characterized by low electron densities and reduced gradients approaching zero. In contrast, covalent bonds possess reduced gradients approaching zero but also higher electron densities and regions far from the molecule possess low electron densities but highly reduced gradients, such that regions of non-covalent interactions can unambiguously be identified using both quantities. The type of interaction is determined with the sign of the second-largest eigenvalue of the Hessian ($\mathrm{sign}(\lambda_2)$), where a positive sign indicates repulsive interactions like steric effects and a negative sign indicates attractive interactions like hydrogen bonds (Supplementary Fig. 8). Together with the fact that the electron density is higher in regions of stronger interactions and lower in regions of weaker interactions (like van der Waals interactions), different types of non-covalent interactions can be identified when plotting the RDG against $\mathrm{sign}(\lambda_2)\rho$. Showing isosurfaces of the reduced density gradient reveals the areas of intramolecular interactions in the molecule.[22]

Colored RDG-scatter plots show blue spikes indicating hydrogen bonding for isomers **A-1**, **D-1** and **E-1** (Supplementary Fig. 8). On the other hand, the lack of blue spikes confirms the absence of hydrogen bonding for *Z*-configured isomers **B-1** and **C-1**. Furthermore, in all isomers red spikes indicate the presence of steric effects and green spikes van der Waals interactions. The regions of each interaction are visualized as colored disks and can be seen in Supplementary Fig. 9.

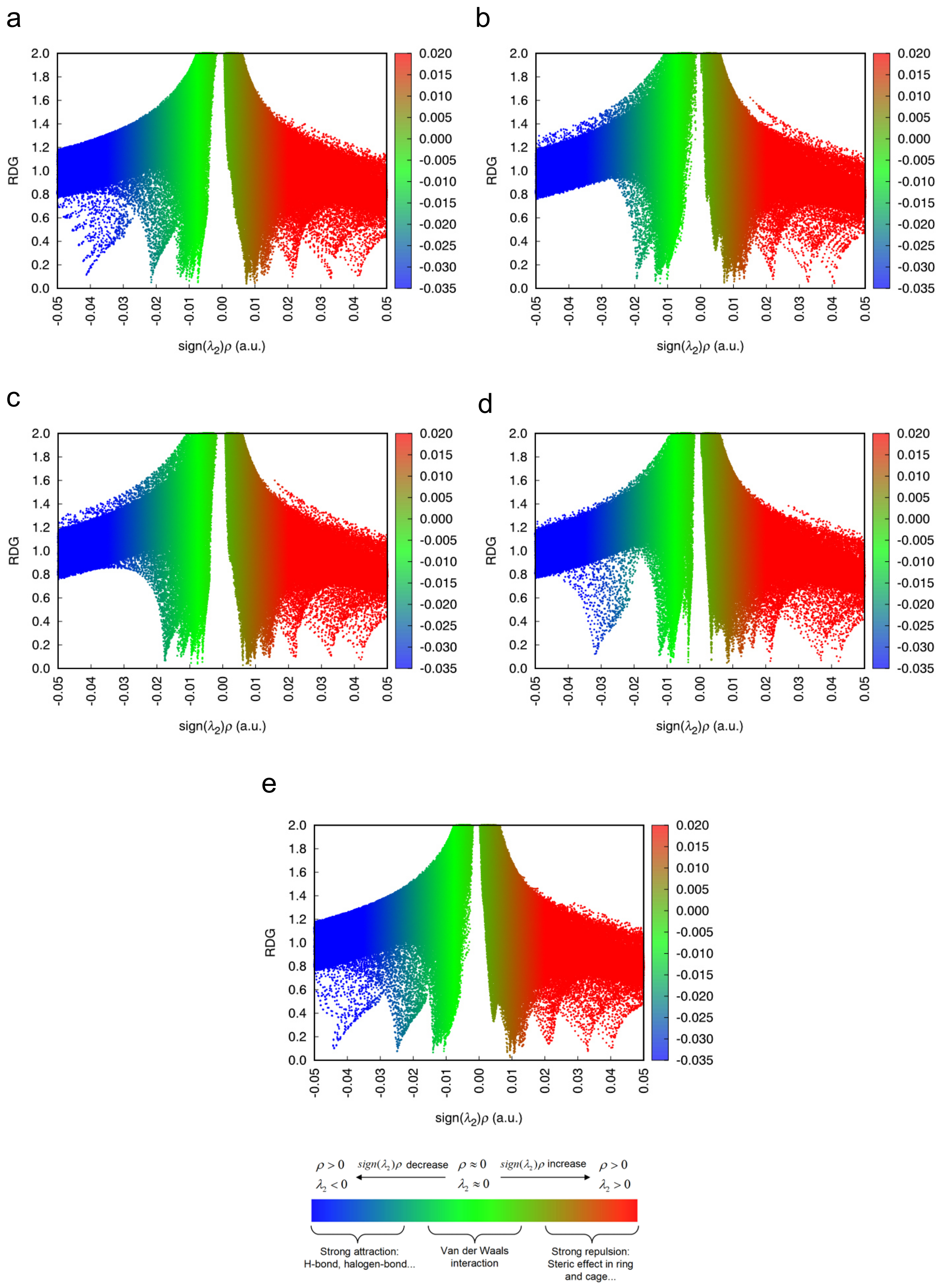

Supplementary Figure 8. Colored RDG plots for all isomers. **a) A-1**, **b) B-1**, **c) C-1**, **d) D-1**, and **e) E-1.**

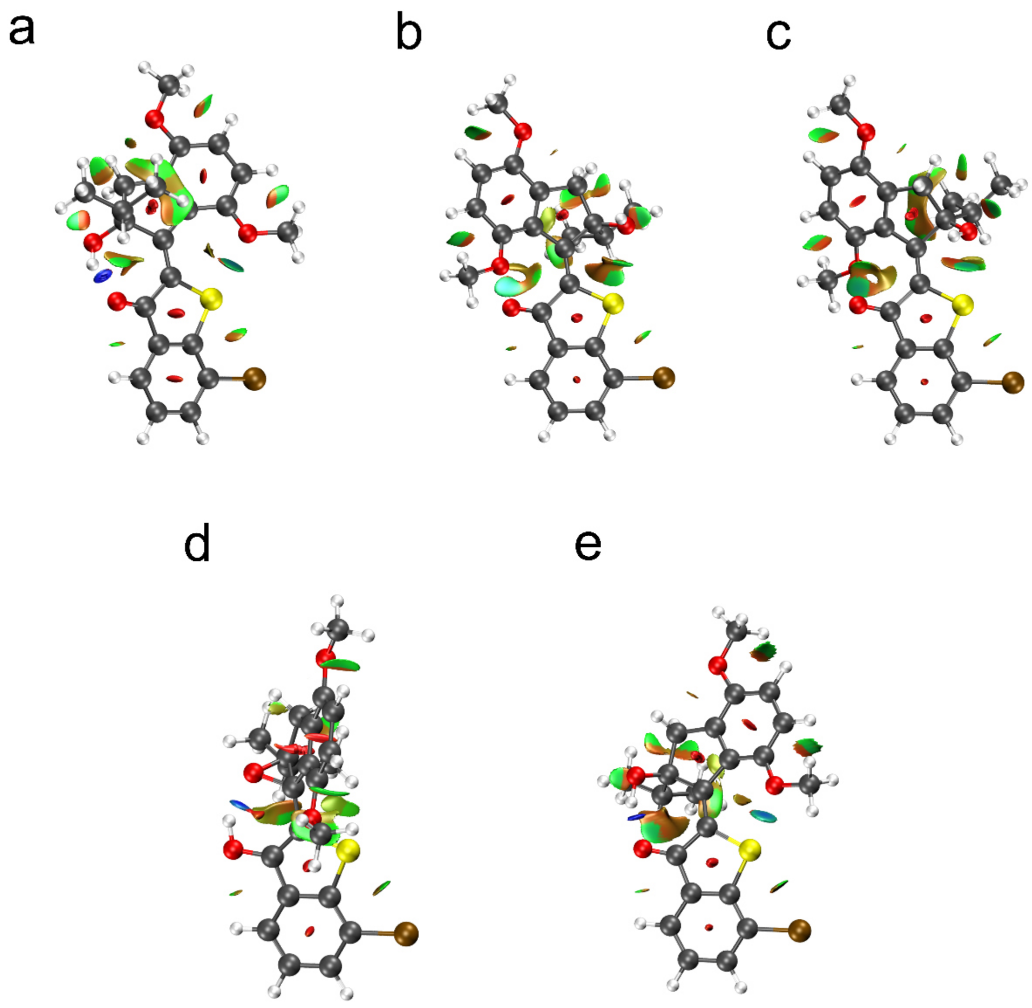

Supplementary Figure 9. Visualization of regions of non-covalent interactions using the RDG-isosurface of 0.5. **a) A-1**, **b) B-1**, **c) C-1**, **d) D-1**, and **e) E-1**. Blue disks in isomers **A-1**, **D-1** and **E-1** indicate hydrogen bonding. No hydrogen bonding is indicated for isomers **B-1** and **C-1**. Red forms show steric effects and greenish shapes illustrate van der Waals interactions in all isomers.

Binding Energy Calculations

In order to approximate and compare the strengths of the hydrogen bonding interactions among the isomers **A-1**, **D-1** and **E-1**, the strength of each hydrogen bond was estimated as suggested by Emamian *et al.*[23] using the fitted Equation 5:

$$BE = -223.08 \cdot \rho BCP + 0.7423 \quad (5)$$

where *BE* is the binding energy in kcal/mol and ρ_{BCP} stands for the electron density at the bond critical point (BCP) of the hydrogen bond, according to atoms in molecules (AIM) theory.[24]

For isomer **A-1**, a binding energy of −6.44 kcal/mol is obtained and the O–H distance is calculated as 1.74 Å. The hydrogen bond in the epoxide intermediate **D-1** is considerably stronger with −8.51 kcal/mol, agreeing well with the large shift in ^{1}H NMR, however the distance is calculated as 1.86 Å. Considering the **E-1** isomer, the hydrogen is predicted to be slightly stronger with a binding energy of −9.13 kcal/mol and the distance between OH donor and C=O acceptor is only 1.72 Å.

4. Theoretical Description of the Excited State of Motor 1

Quantum chemical calculations were performed using **Gaussian 16**,[1] **Orca 5**,[3,4] **GAMESS 2023 R1**,[5] and OpenQP[6,7,8,9] to characterize the excited state properties of the studied systems.

To investigate absorption properties, **time-dependent DFT (TD-DFT)** calculations were performed for **A-1, B-1, C-1, and D-1**. Vertical transition energies, oscillator strengths, and the electronic character of transitions were obtained at the **TD-CAM-B3LYP/6-311G(d,p)** level of theory. Simulated **ECD and UV/Vis absorption spectra** were based on the lowest **30 singlet states**, incorporating solvent effects for **Et_2O (dielectric constant (ε) = 7.43)** using a non-equilibrium solvation model.

Furthermore, multiconfigurational *ab initio* calculations were performed for isomer **C-1** and **D-1** using the complete active space self-consistent field (CASSCF) method, followed by second-order perturbation theory corrections (CASPT2) to account for dynamic correlation effects. For both, the cc-pVDZ[25] basis set was employed. Active spaces comprising 8, 10, 12, and 14 active electrons in 8, 10, 12, and 14 active orbitals, respectively, were utilized, denoted as (8,8), (10,10), (12,12), and (14,14). Each calculation was performed with state averaging over three singlet states.

To investigate key excited-state structures relevant to the photochemical process, we placed our focus on critical **static points**:

1. The **S_1/S_0 conical intersection (CI_{10}) between C-1** and **D-1**, which plays a key role in the photochemical reaction pathway.
2. The **minimum energy path (MEP)** connecting the equilibrium geometry of **C-1** *via* **CI_{10}** to the equilibrium geometry of **D-1** on multiple potential energy **surfaces**.

The **CI_{10} geometry** was optimized using **spin-flip TDDFT (SF-TDDFT)** and **mixed-reference SF-TDDFT (MRSF-TDDFT)** as implemented in **Orca** and **OpenQP**, respectively. These calculations employed the **BHandHLYP[26]/def2-TZVP[27,28] and DTCAM-AEE[29]/def2-SVP** functional, respectively, and were conducted in the **gas phase** (no solvent effects). To take potential tilt or pyramidalization[30] at the central double bond in minimum energy conical intersection points into account, we considered different structural guesses for the optimization of conical intersections, all of which resulted in the same final structure (see Supplementary Figures 16 and 17).

The **minimum energy path (MEP)** from **C-1 (S_1)** to **CI_{10}** was determined using **nudged elastic band (NEB) calculations** at the **MRSF-TDDFT/BHHLYP[31]/def2-SVP** level in **GAMESS**, optimizing along the **S_1 state**. **3 singlet** and 2 **triplet states** were included in the calculations.

The reaction path connecting **CI_{10} and D-1** was obtained at the **CAM-B3LYP/def2-TZVP** level of theory using **Orca**, considering this process on both **S_0 and T_1 potential energy surfaces**. To assess environmental effects, calculations were performed both **in vacuum** and with implicit solvent models for **THF** and **water** using the **linear response conductor-like polarizable continuum model[32] (LR-CPCM)**.

4.1 Excitation analysis based on time-dependent density functional theory

Supplementary Table 5. Vertical excitation energies (VEEs) and oscillator strengths (*f*) calculated at the CAM-B3LYP/ 6-311G(d,p)/IEFPCM(Et_2O) level of theory for isomers **A-1** to **E-1**.

	S_1		S_2	
isomer	VEE / eV	*f*	VEE / eV	*f*
**A-1**	3.11	0.297	3.45	0.065
**B-1**	2.97	0.327	3.40	0.068
**C-1**	3.21	0.272	3.58	0.060
**D-1**	4.52	0.264	4.66	0.124
**E-1**	2.98	0.324	3.42	0.033

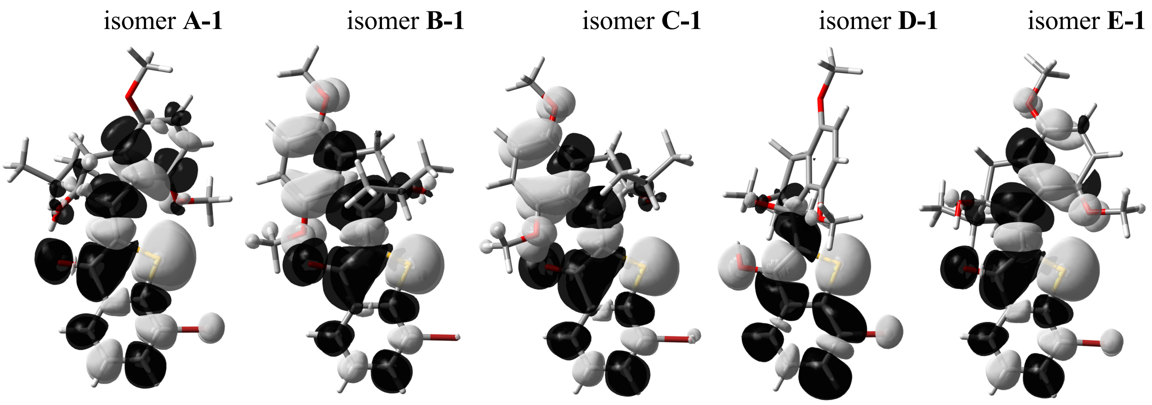

Supplementary Figure 10. Charge-density differences for $S_0 \rightarrow S_1$ transitions calculated at the CAM-B3LYP/ 6-311G(d,p)/IEFPCM(Et_2O) level of theory for isomers A-E. Black indicates regions of increased and grey regions of decreased electron density upon excitation.

4.2 Comparison of experimental and theoretical UV/vis and ECD Spectra

Good agreement between the simulated and experimental spectra not only supports the unidirectional rotation of motor **1** through the **A**, **B**, **C** and **D** state interconversion sequence but also allowed for assigning the absolute configuration by comparison of theoretical and experimental ECD spectra. The related corresponding spectra are shown in Supplementary Figures 11 and 12.

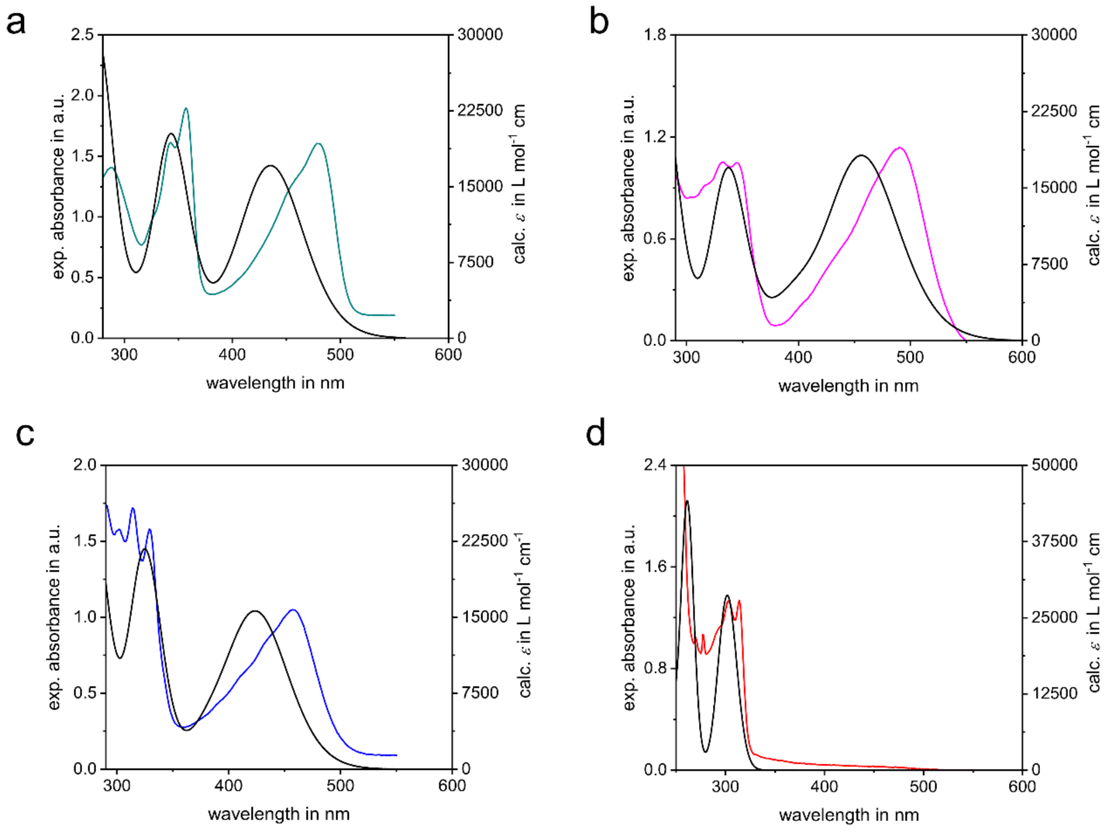

Supplementary Figure 11. Experimental (black) and calculated (colored) UV/Vis spectra for isomers of motor **1**. a) **A-1**, b) **B-1**, c) **C-1** and d) **D-1**. All experimental spectra were acquired in EPA (mixture of Et_2O:*i*-pentane:EtOH, 5:5:2 v/v). Experimental spectra of **A-1** and **B-1** isomers were measured at −160 °C, where the UV/Vis spectrum of **B-1** was obtained after 101 sec irradiation of **A-1** isomer at the same temperature. The experimental UV/Vis spectra of **C-1** and **D-1** were recorded at −120 °C and the spectrum of **D-1** was obtained after 69 min irradiation of **C-1** isomer at this temperature. Theoretical calculations were performed at the CAM-B3LYP/6-311G(d,p)/PCM(Et_2O) level of theory. Gaussian broadening of σ = 0.30 eV (0.20 eV for epoxide **D-1**) applied for all calculated spectra and red-shifted by 40 nm (30 nm for epoxide **D-1**).

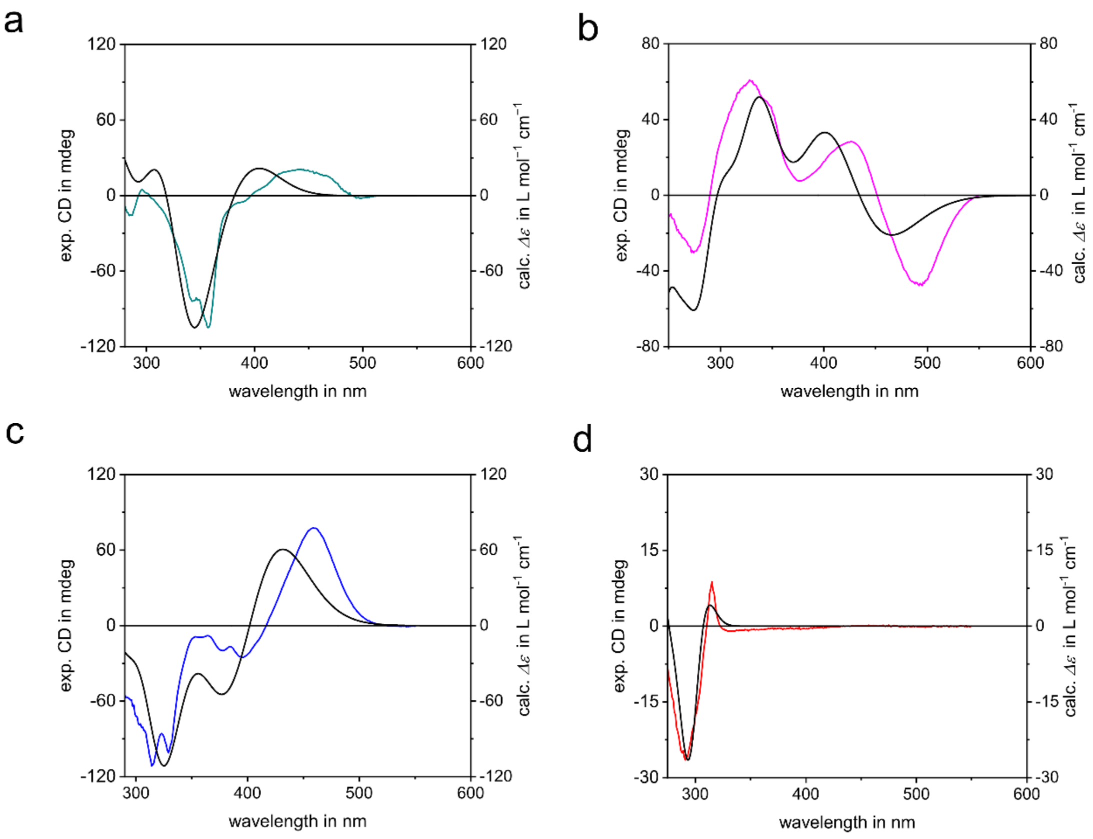

Supplementary Figure 12. Experimental (black) and calculated (colored) ECD spectra for isomers of motor **1**. a) **A-1**, b) **B-1**, c) **C-1** and d) **D-1**. All experimental spectra were acquired in EPA (mixture of Et_2O:*i*-pentane:EtOH, 5:5:2 v/v). Experimental spectra of **A-1** and **B-1** isomers were measured at −160 °C, where the ECD spectrum of **B-1** was obtained after 161 sec irradiation of **A-1** isomer with (*S*) configured stereocenter at the same temperature and both spectra were inverted for better comparison. The experimental ECD spectra of **C-1** and **D-1** were recorded at −120 °C and the spectrum of **D-1** was obtained after 69 min irradiation of **C-1** isomer at this temperature. Theoretical calculations were performed at the CAM-B3LYP/6-311G(d,p)/PCM(Et_2O) level of theory. Gaussian broadening of $\sigma = 0.30$ eV (0.20 eV for epoxide **D-1**) employed for all calculated spectra and red-shifted by 40 nm (30 nm for epoxide **D-1**). The theoretical ECD spectra of **A-1** was scaled by 1.87 and 1.44 for **B-1**. For **C-1** and **D-1** the scaling factors of 2.64 and 1.16 were applied, respectively.

4.3 Multiconfigurational excitation analysis and active spaces

Supplementary Table 6. Vertical excitation energies (VEEs) and oscillator strengths (*f*) for isomers **C-1** and **D-1**, computed at the CASSCF/cc-pVDZ and CASPT2/cc-pVDZ levels of theory using four different active spaces. The computational level (active space) that best aligns with the experimental UV/Vis absorption spectrum, considering the position and relative intensity of excitations into S1 and S2, is highlighted in green.

**C-1**			**S_1 VEE/eV**	***f***	**S_2 VEE/eV**	***f***
	**CASSCF**	8-8	4.3	0.25	5.1	0.60
		10-10	4.8	0.23	5.2	0.33
		12-12	4.2	0.18	4.7	0.09
		14-14	4.2	0.17	4.7	0.10
	**CASPT2**	8-8	2.8	0.16	3.3	0.39
		10-10	2.6	0.17	2.9	0.14
		12-12	2.8	0.12	3.2	0.06
		14-14	2.8	0.11	3.2	0.07
**D-1**						
	**CASSCF**	8-8	5.7	0.01	6.0	0.78
		10-10	5.7	0.02	6.6	0.29
		12-12	4.8	0.00	6.2	0.14
		14-14	4.8	0.01	201.4	0.17
	**CASPT2**	8-8	3.8	0.50	4.5	0.01
		10-10	4.3	0.01	4.5	0.20
		12-12	4.4	0.00	5.3	0.12
		14-14	4.3	0.01	5.0	0.14

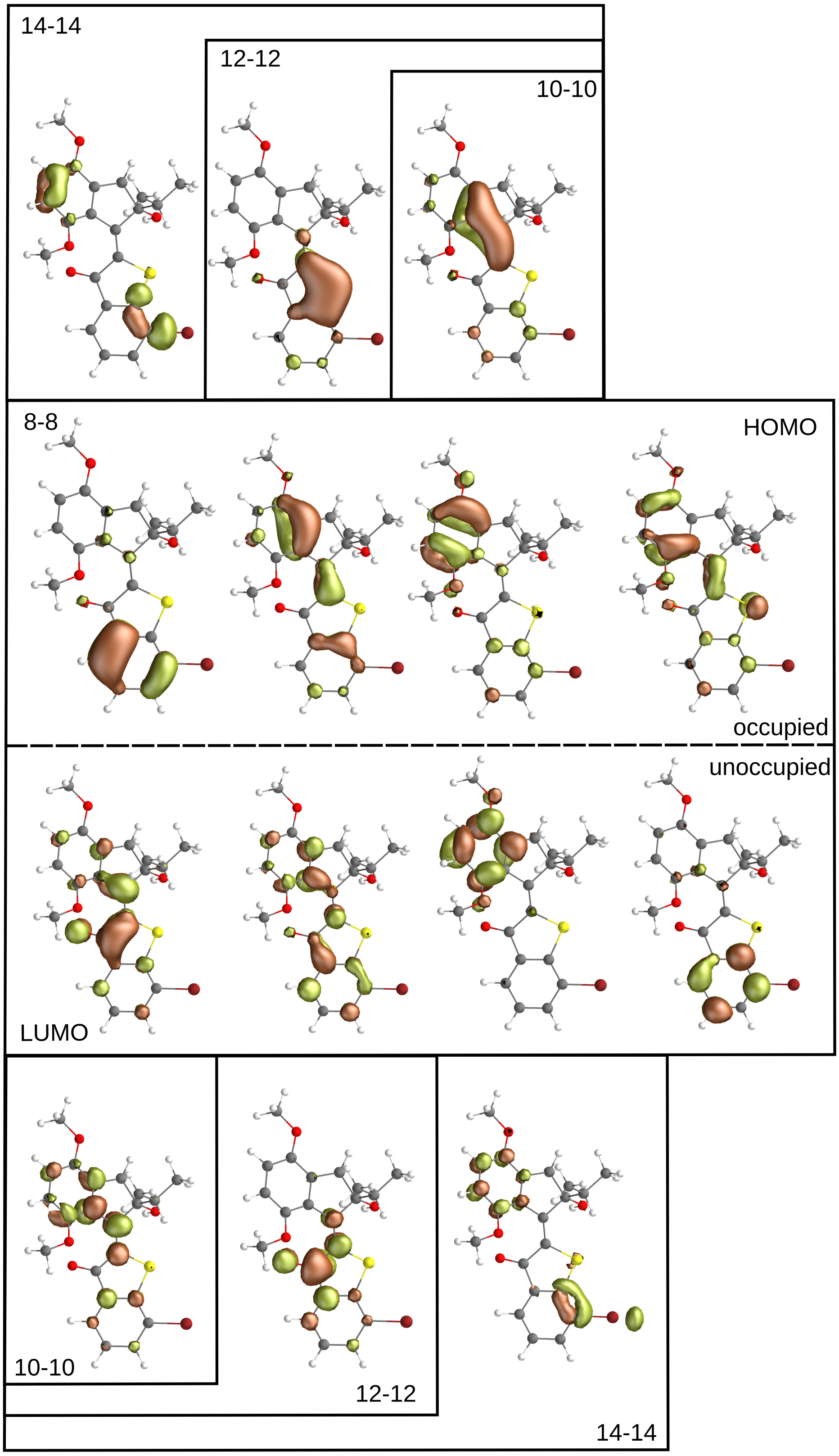

Supplementary Figure 13. Visualization of the molecular orbitals of **C-1** employed in the CASSCF simulations. The smallest active space, comprising 8 active electrons in 8 active orbitals, is depicted at the center. Successive expansion of the active space is achieved by systematically incorporating one additional occupied and one additional unoccupied orbital, yielding the (10,10), (12,12), and (14,14) active spaces, as indicated by the respective boxes. This systematic extension ensures consistency, as all orbitals included in smaller active spaces are retained in the larger ones.

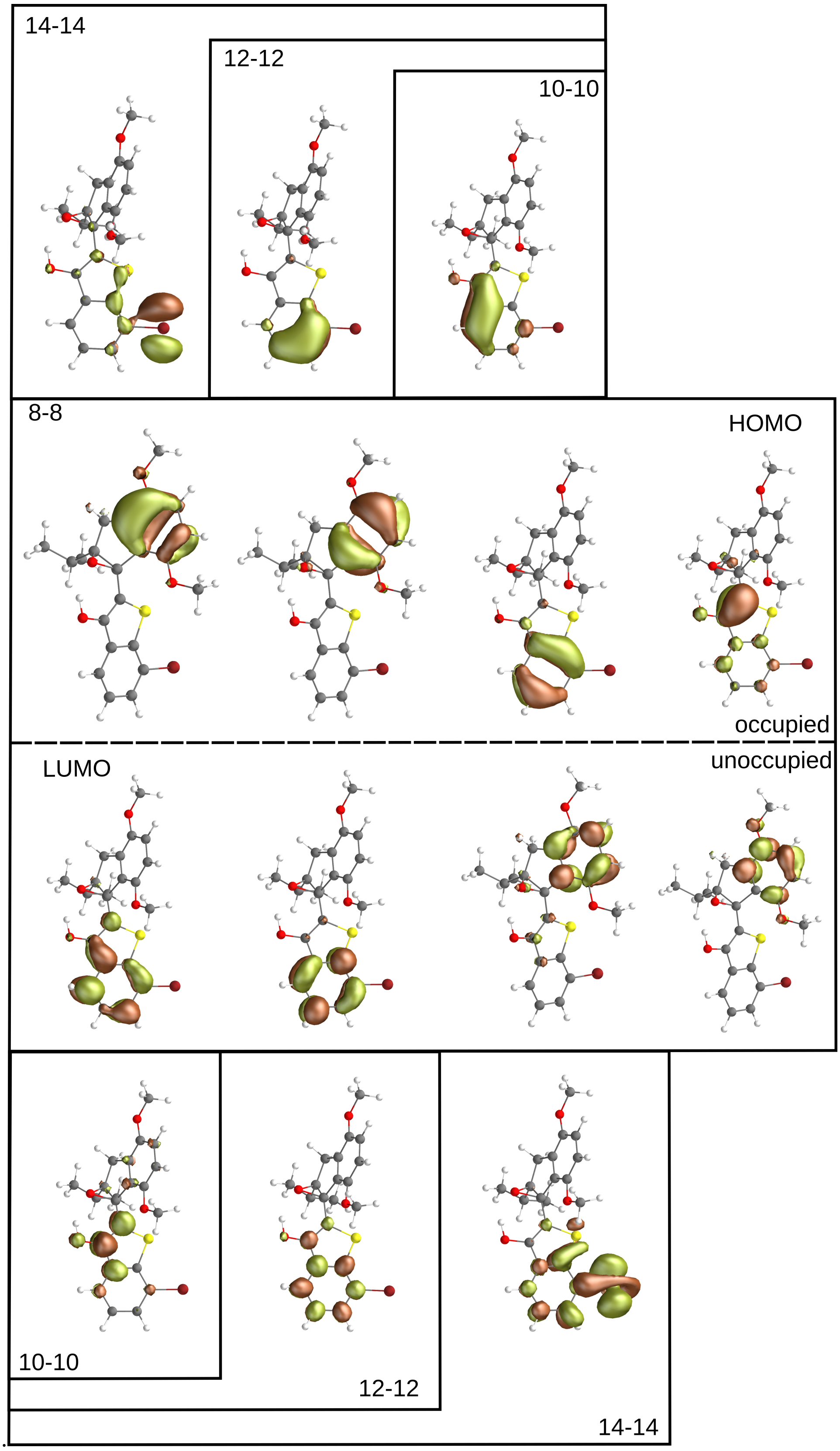

Supplementary Figure 14. Visualization of the molecular orbitals of **D-1** employed in the CASSCF simulations. The smallest active space, comprising 8 active electrons in 8 active orbitals, is depicted at the center. Successive expansion of the active space is achieved by systematically incorporating one additional occupied and one additional unoccupied orbital, yielding the (10,10), (12,12), and (14,14) active spaces, as indicated by the respective boxes. This systematic extension ensures consistency, as all orbitals included in smaller active spaces are retained in the larger ones.

4.4 Conical intersections optimizations

We tested our level of theory on the main MECIs of interest on the core structure of unsubstituted hemithioindigo. More precisely, we used MRSF to optimize CoIn HT, Et, S, S*, Ac and CHD from an earlier study on hemithioindigo.[30] We were able to reproduce all tested CIs, with an average RMSD of 0.15 Å. Two structures (S and S*) have a slightly higher but still satisfactory RMSD of 0.31 and 0.35 Å, whereas the other four show excellent agreement with RMSDs of 0.09, 0.10, 0.04 and 0.03Å for Ac, CHD, Et and HT, respectively.

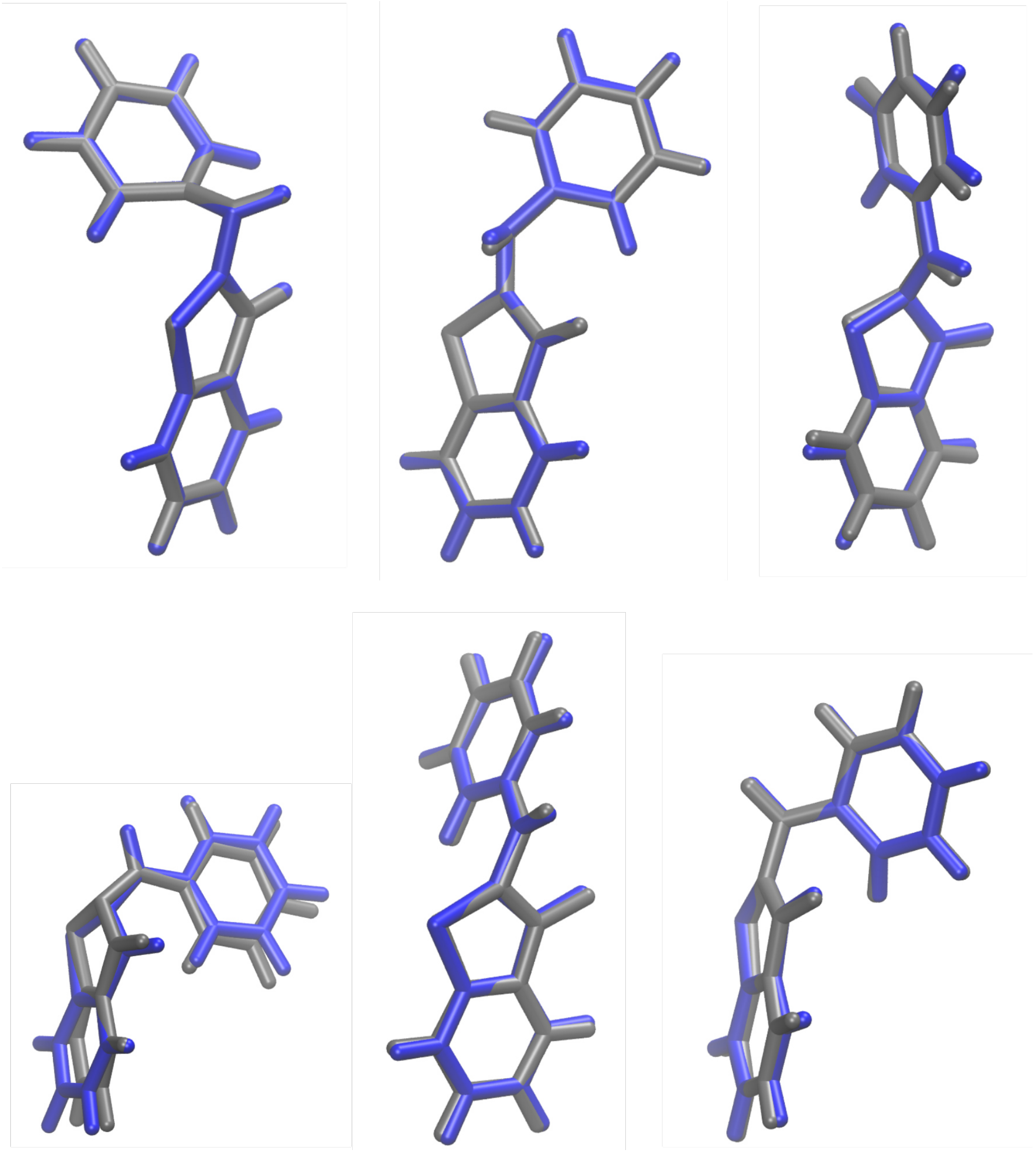

Supplementary Figure 15. Structure of the optimized S_1/S_0 CI of hemithioindigo obtained with MRSF-TDDFT (blue) and their reference structures (grey). First row: l.t.r. HT-, Et-, and S-type CI. Second row: l.t.r. S*-, Ac- and CHD-type CI.

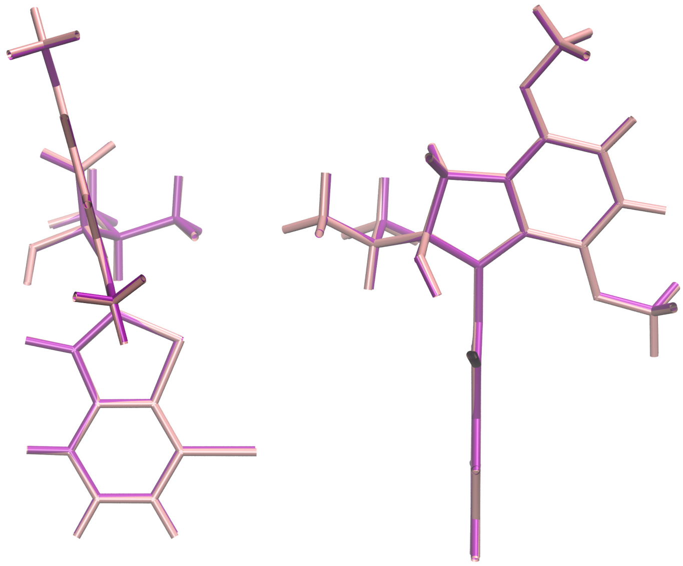

Supplementary Figure 16. Structure of the optimized S_1/S_0 CI of motor **1** obtained with MRSF-TDDFT (purple) and SF-TDDFT (light pink).

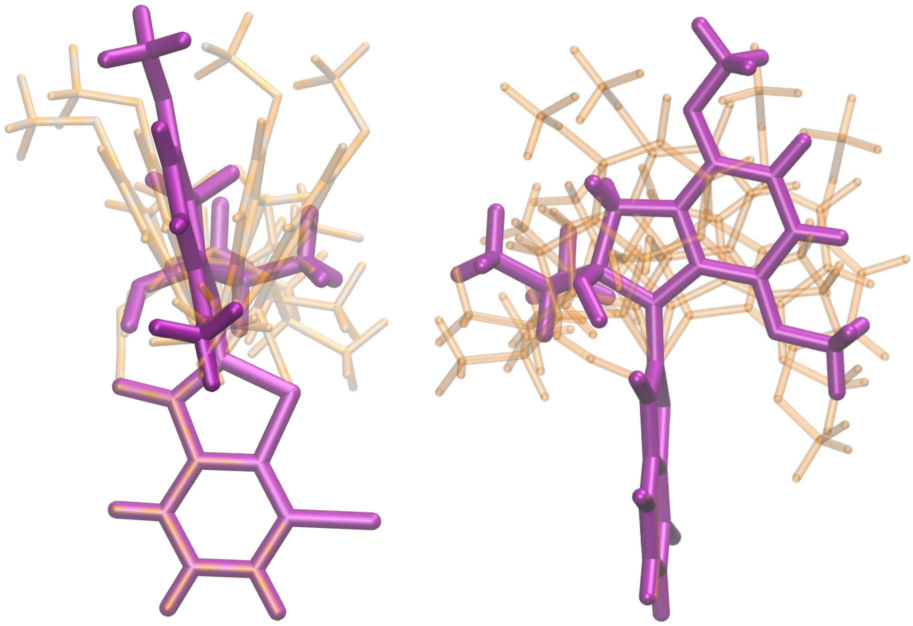

Supplementary Figure 17. Structural guesses of motor **1** with different degrees of tilt (left) or pyramidalization (right) in transparent orange and structure of the only optimized minimum energy conical intersection in purple.

4.5 Minimum energy path calculations

To explore the pathway connecting **C-1** upon excitation to the S_1 state to **D-1**, we explored the minimum energy path connecting **C-1**(S_1) *via* the conical intersection of S_1 and S_0 with **D-1** (S_0).

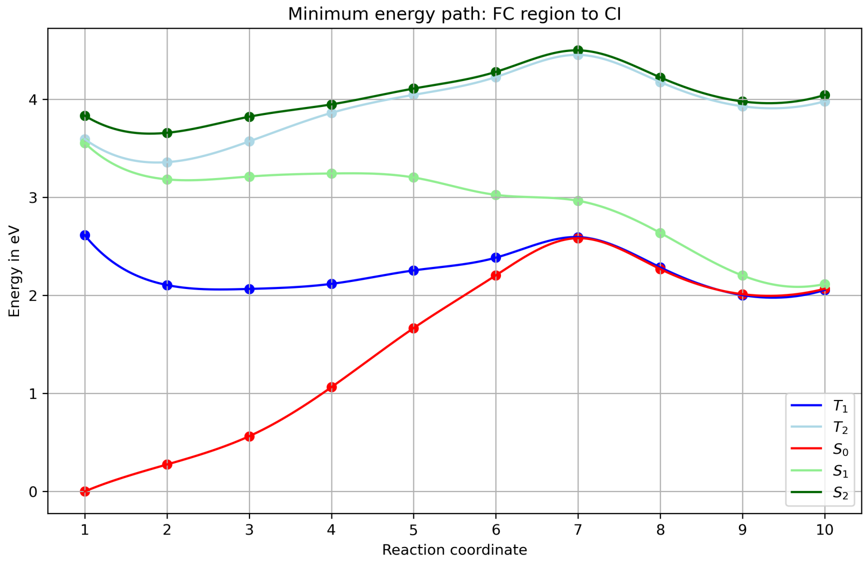

Supplementary Figure 18. Optimized minimum energy path (MEP) on the S_1 potential energy surface (PES) connecting **C-1** (1: ground-state minimum geometry of **C-1**) to the S_1/S_0 conical intersection (10: CI_{10}). The path was optimized on the S_1 state using MRSF-TDDFT/def2-SVP/gas phase. Energies for the S_0, S_2, T_1, and T_2 states were computed along the S_1-optimized path at the same level of theory.

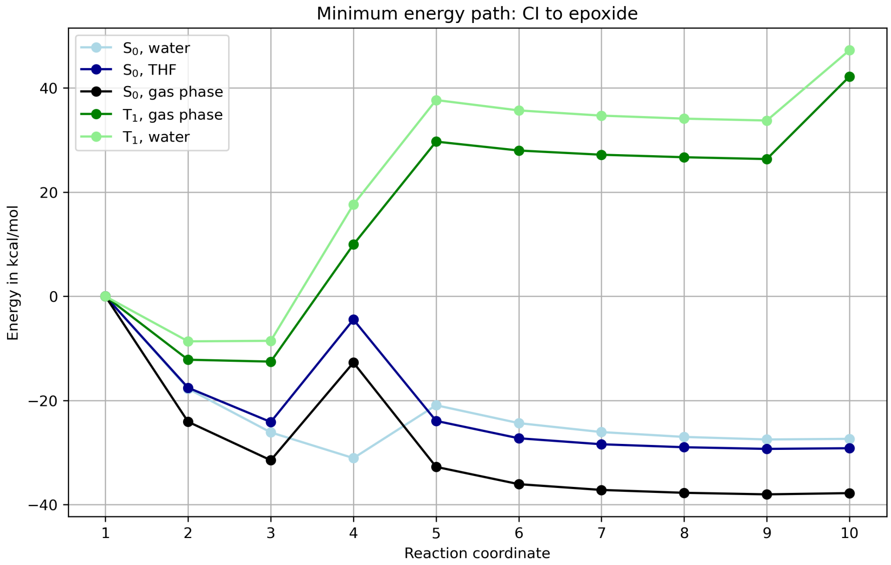

Supplementary Figure 19. Optimized minimum energy path (MEP) on the S_0 (blue curves) and T_1 (green curves) potential energy surfaces, connecting the S_1/S_0 conical intersection (1: CI_{10}) to the equilibrium geometry of **D-1** (10). The paths were optimized separately on the S_0 and T_1 states using CAM-B3LYP/def2-TZVP. For each environment — vacuum, IEFPCM (THF), and IEFPCM (water) — independent optimizations were performed, meaning that points 2 to 9 along the reaction coordinate do not correspond to identical geometries across S_0, T_1, or different solvation conditions.

5. Syntheses of Compounds

Synthesis of HTI 1

The target HTI motor **1** was prepared from the condensation reaction between indanone **6** and thiophenone **7** catalyzed by BCl_3 as Lewis acid. Starting from commercially available acid **3**, indanone **6** was synthesized within a three-step procedure including optimized conditions for the introduction of the hydroxy group.[33] On the other hand, commercially available thiophenol **10** afforded the target thiophenone **7** through a literature-known protocol.[34] The overall synthetic route for the construction of HTI **1** is depicted in Scheme 1.

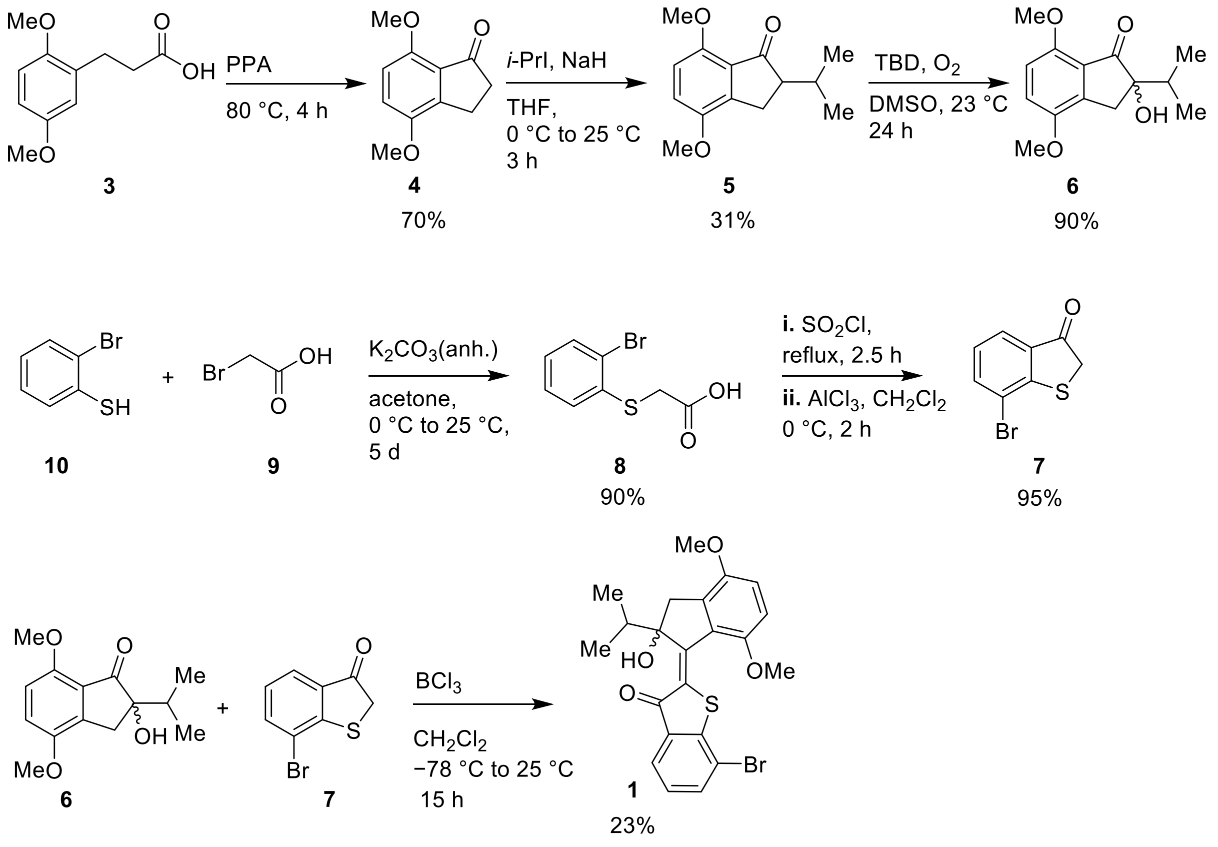

Scheme 1. Synthetic route for construction of motor **1**. PPA: polyphosphoric acid; TBD: 1,5,7-triazabicyclo[4.4.0]dec-5-ene.

4,7-dimethoxy-2,3-dihydro-1*H*-inden-1-one (4)[35]

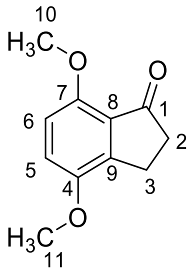

A mixture of 3-(2,5-dimethoxyphenyl)propanoic acid **3** (4.68 g, 22.3 mmol, 1.0 equiv.) and polyphosphoric acid (50.9 g) was stirred at 80 °C for 4 h. After the reaction was completed, 400 mL of ice water was added and the resulting mixture was extracted with EtOAc (3 x 100 mL). The combined organic phases were washed with a saturated aqueous solution of $NaHCO_3$ (200 mL) and brine (200 mL), dried over anhydrous Na_2SO_4, and filtered. The filtrate was dried under vacuum and product **4** (3.00 g, 15.6 mmol, 70%) was obtained as white powder after purification by flash column chromatography (SiO_2, EtOAc:*i*-Hex 35:65, v:v).

***R*f** (SiO_2, EtOAc:*i*-Hex, 35:65 v:v) = 0.33.

^{1}H NMR (400 MHz, $CDCl_3$,): δ / ppm = 6.98 (d, ^3J = 8.6, 1H, H(C5)), 6.73 (d, ^3J = 8.6, 1H, H(C6)), 3.90 (s, 3H, H(C10)), 3.85 (s, 3H, H(C11)), 2.98 (m, 3H, H(C2)), 2.67 (s, 3H, H(C3)).

^{13}C NMR (100 MHz, $CDCl_3$,): δ / ppm = 205.2 (C1), 151.9 (C7), 150.5 (C4), 146.1 (C8), 126.4 (C9), 116.7 (C5), 109.5 (C6), 56.1 (C10), 56.0 (C11), 36.9 (C2), 22.4 (C3).

HRMS (EI) calcd. for $[C_{11}H_{12}O_3]^+$: 192.0786; found: 192.0783.

Melting point: 122 °C.

2-*iso*propyl-4,7-dimethoxy-2,3-dihydro-1*H*-inden-1-one (5)

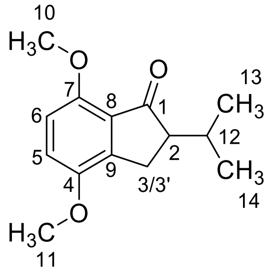

In a round bottom flask, compound **4** (540 mg, 2.80 mmol, 1.0 equiv.) was dried under vacuum and then set under nitrogen atmosphere. After the addition of dry THF (10 mL), the solution was cooled to 0 °C using an ice bath. Then, NaH (102 mg, 4.10 mmol, 1.46 equiv.) was added to the reaction mixture under nitrogen atmosphere and the resulting suspension was stirred for

30 min at 0 °C. To this mixture, isopropyl iodide (2.50 mL, 4.10 mmol) was added and the reaction was stirred at 23 °C for 20 h. Subsequently, the reaction was quenched with ice cold water (200 mL). The volatiles were removed under vacuum and the residue was mixed with EtOAc (200 mL). The organic layer was extracted with EtOAc (3 x 200 mL) and dried over anhydrous Na_2SO_4. After filtration, purification was performed by flash column chromatography (SiO_2, EtOAc:*i*-Hex 35:65) to afford the product **5** (200 mg, 0.85 mmol, 31%) as colorless semi-solid.

***R*f** (SiO_2, EtOAc:*i*-Hex, 35:65 v:v) = 0.44.

^{1}H NMR (400 MHz, $CDCl_3$): δ / ppm = 6.97 (d, ^3J = 8.6 Hz, 1H, H(C5)), 6.71 (d, ^3J = 8.6 Hz, 1H, H(C6)), 3.89 (s, 3H, H(C10)), 3.86 (s, 3H, H(C11)), 2.99 (dd, ^2J = 16 Hz, ^3J = 8 Hz, 1H, H(C3)-H), 2.76 (dd, ^2J = 16 Hz, ^3J = 8 Hz, 1H, H′(C3)), 2.62 (dt, ^3J = 8 Hz, ^3J = 4 Hz, 1H, H(C2)), 2.41 (dsept, ^3J = 6.8 Hz, ^3J = 4.4 Hz, 1H, H(C12)), 1.04 (d, ^3J = 7.2 Hz, 3H, H(C13/C14)), 0.78 (d, ^3J = 7.2 Hz, 3H, H(C13/C14)).

^{13}C NMR (100 MHz, $CDCl_3$): δ / ppm = 206.8 (C1), 151.6 (C7), 150.3 (C4), 145.0 (C8), 126.8 (C9), 116.4 (C5), 109.3 (C6), 56.0 (C10), 55.8 (C11), 53.2 (C2), 29.1 (C3), 24.5 (C12), 20.9 (C13/C14), 17.0 (C13/C14).

HRMS (EI) calcd. for $[C_{11}H_{12}O_3]^+$: 234.1256; found: 234.1258.

IR: $\tilde{\nu}$/cm^{-1} = 2958 (m), 2935 (m), 2907 (w), 2871 (w), 2834 (w), 1705 (s), 1592 (m), 1491 (s), 1461 (m), 1447 (m), 1435 (m), 1416 (w), 1384 (w), 1368 (w), 1331 (w), 1315 (w), 1284 (m), 1257 (s), 1214 (m), 1193 (m), 1178 (w), 1164 (w), 1140 (w), 1101 (m), 1061 (s), 1031 (m) , 1001 (m) , 970 (m), 945 (w), 914 (w), 905 (w), 815 (m), 807 (m), 792 (m), 773 (m), 718 (m), 706 (m), 678 (w), 650 (w), 619 (w), 578 (w), 510 (w), 501 (w), 476 (w), 458 (w), 440 (w), 432 (w), 424 (w), 417 (w), 401 (w).

Melting point: 60 °C.

2-hydroxy-2-*iso*propyl-4,7-dimethoxy-2,3-dihydro-1*H*-inden-1-one (6)

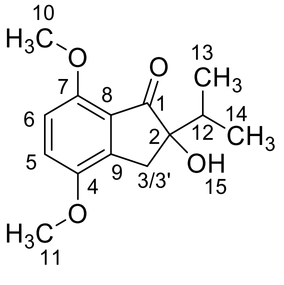

Into a Schlenk tube, compound **5** (50 mg, 0.21 mmol, 1.0 equiv.) and 1,5,7-triazabicyclo[4.4.0]dec-5-ene (29.2 mg, 0.21 mmol, 1.0 equiv.) were added and kept under vacuum for 2 h. Then, DMSO (0.84 ml) was added and, after three further degassing cycles, three balloons filled with oxygen gas were connected to the flask to keep the oxygen content of the reaction as high as possible. The resulting mixture was stirred for 24 h at 23 °C and then quenched with 2 M aqueous HCl (10 mL). The aqueous phase was extracted with EtOAc (3 x 30 mL). The combined organic phases were washed with brine (50 mL) and dried over anhydrous Na_2SO_4. The crude product was purified via flash column chromatography (SiO_2, gradient from EtOAc:*i*-Hex 35:75 to 50:50, v:v) affording **6** (47.3 mg, 0.19 mmol, 90%) as a colorless solid.

***R*f** (SiO_2, EtOAc:*i*-Hex, 35:65 v:v) = 0.16.

^{1}H NMR (400 MHz, $CDCl_3$,): δ / ppm = 7.02 (d, ^3J = 8.6 Hz, 1H, H(C5)), 6.74 (d, ^3J = 8.6 Hz, 1H, H(C6)), 3.90 (s, 3H, H(C10)), 3.85 (s, 3H, H(C11)), 3.18 (d, ^2J = 16 Hz, 1H, H(C3)-H), 2.79 (d, ^2J = 16 Hz, 1H, H′(C3)-H), 2.42 (br s, 1H, H(C15) 2.00 (sept, ^3J = 6.8 Hz, 1H, H(C12)), 1.03 (d, ^3J = 7.2 Hz, 3H, H(C13/C14)), 0.74 (d, ^3J = 7.2 Hz, 3H, H(C13/C14).

^{13}C NMR (100 MHz, $CDCl_3$,): δ / ppm = 206.8 (C1), 152.0 (C7), 150.3 (C4), 142.7 (C8), 124.3 (C9), 117.9 (C5), 109.9 (C6), 82.2 (C2), 56.2 (C10), 56.1 (C11), 35.5 (C3), 33.0 (C12), 17.4 (C13/C14), 16.7 (C13/C14).

HRMS (EI) calcd. for $[C_{11}H_{12}O_3]^+$: 250.1205; found: 250.1208.

Melting point: 143 °C.

IR: $\tilde{\nu}$/cm^{-1} = 3397 (w), 2999 (w), 2961 (w), 2926 (w), 2835 (w), 1686 (m), 1595 (m), 1497 (m), 1465 (w), 1447 (w), 1432 (w), 1417 (w), 1391 (w), 1365 (w), 1348 (w), 1322 (w), 1315 (w), 1269 (m), 1263 (m), 1239 (m), 1193 (m), 1172 (m), 1153 (m), 1128 (m), 1104 (m), 1070 (s), 1054 (s), 1006 (m), 974 (m), 920 (w), 899 (w), 820 (m), 812 (m), 770 (w), 719 (w), 698 (w), 595 (w), 572 (w), 467 (m), 450 8m), 441 (m), 436 (m), 419 (m), 408 (w).

Note: This reaction is very sensitive to the oxygen content of the vessel. Therefore, the best yields are achievable for small scale synthesis as described in the synthetic procedure, which is adopted from the literature.

2-((2-bromophenyl)thio)acetic acid (8)[34]

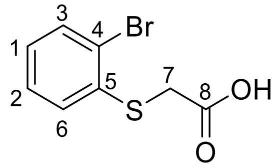

To a solution of acid **9** (2.50 g, 18.0 mmol, 1.1 equiv.) in acetone (170 mL), anhydrous K_2CO_3 (7.00 g, 50.6 mmol, 3.0 equiv.) and thiophenol **10** (2.0 mL, 16.6 mmol, 1.0 equiv.) were added at 0 °C. The suspension was allowed to warm to 25 °C and it was stirred for 5 days. The reaction was stopped by the addition of aqueous HCl (2.0 M, 200 mL). Acetone was removed under vacuum and the resulting precipitate was separated by filtration, washed with deionized H_2O (150 mL) and dried under high vacuum. Compound **8** (3.60 g, 14.6 mmol, 88%) was obtained as colorless microcrystalline solid, which was used without further purification.

^{1}H NMR (400 MHz, $(CD_3)_2SO$): δ / ppm = 12.93 (s, 1H, CO_2H), 7.60 (dd, ^3J = 8.0 Hz, ^4J = 1.2 Hz, 1H, H(C3)), 7.37 (ddd, ^3J = 8.5, 7.3 Hz, ^4J =1.4 Hz, 1H, H(C1)), 7.30 (dd, ^3J = 8.0 Hz, ^4J =1.6 Hz, 1H, H(C6)), 7.10 (ddd, ^3J = 7.9, 7.2 Hz, ^4J =1.6 Hz, 1H, H(C2)), 3.90 (s, 2H, H(C7)).

^{13}C NMR (100 MHz, $(CD_3)_2SO$): δ / ppm = 170.1 (C8), 137.2 (C5), 132.6 (C3), 128.3 (C1), 126.7 (C2/C6), 126.6 (C2/C6), 120.8 (C4), 34.2 C7).

HRMS (EI) calcd. for $[C_8H_7BrO_2S]^+$: 245.9350; found: 245.9344.

Melting point: 121 °C.

7-bromobenzo[b]thiophen-3(2H)-one (7)[34]

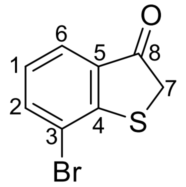

Compound **8** (3.60 g, 14.6 mmol, 1.0 equiv.) was refluxed in thionyl chloride (9.0 mL, 123 mmol, 8.5 equiv.) for 2.5 h. The excess of thionyl chloride was removed under vacuum and the residue was dissolved in dry CH_2Cl_2 (60 mL). At 0 °C, $AlCl_3$ (3.70 g, 27.7 mmol, 1.9 equiv.) was added and the mixture was stirred under nitrogen atmosphere at 0 °C for 2 h. The reaction mixture was poured into ice water (300 mL) and the mixture was extracted with CH_2Cl_2 (2 × 200 mL). The combined organic phases were dried over Na_2SO_4, filtered, and concentrated

under vacuum to give compound **7** (3.20 g, 14.0 mmol, 96%) as a light pink solid, which was used without further purification.

^{1}H NMR (400 MHz, CDCl$_3$): *δ* / ppm = 7.76-7.72 (m, 2H, H(C6 and C2)), 7.14 (t, ^3J = 7.8 Hz, 1H, H(C1)), 3.84 (s, 2H, H(C7)).

^{13}C NMR (100 MHz, CDCl$_3$): *δ* / ppm = 199.6 (C8), 155.5 (C4), 138.2 (C2), 133.1 (C5), 126.3 (C1), 125.4 (C6), 118.9 (C3), 40.3 (C7).

HRMS (EI) calcd. for [C_8H_5BrOS]$^+$: 227.9245; found: 227.9238.

Melting point: 110 °C.

(*E*/*Z*)-7-bromo-2-(2-hydroxy-2-*iso*propyl-4,7-dimethoxy-2,3-dihydro-1*H*-inden-1-ylidene)benzo[*b*]thiophen-3(2*H*)-one ((*E*/*Z*)-1)

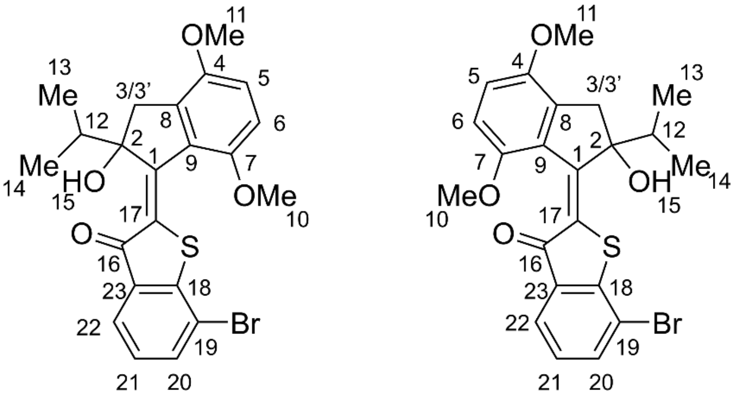

In a flame-dried Schlenk flask, a solution of thiophenone **7** (132 mg, 0.58 mmol, 1.2 equiv.) in dry CH_2Cl_2 (3 mL) was cooled to −78 °C under nitrogen atmosphere. Subsequently, BCl_3 (1 M in CH_2Cl_2, 0.6 mL, 0.60 mmol, 1.3 equiv.) was added. The mixture was immediately taken up by a syringe (an additional 2 mL of dry CH_2Cl_2 were added to rinse the flask) and added to a second Schlenk-flask containing a solution of indanone **6** (120 mg, 0.48 mmol, 1.0 equiv.) in dry CH_2Cl_2 (1.5 mL) at 0 °C. The reaction mixture was allowed to warm to 25 °C and it was stirred at this temperature for 15 h. The reaction was stopped by the addition of deionized H_2O (5 mL) and the mixture was diluted with H_2O (30 mL) and extracted with EtOAc (3 × 50 mL). The combined organic phases were dried over Na_2SO_4, filtered and concentrated under vacuum. The crude product was purified by flash column chromatography (SiO_2, CH_2Cl_2) to give the title compound **1** (50 mg, 0.11 mmol, 23%) as only *E*-isomer as an orange solid. The *Z*-configured isomer was obtained as an orange solid after irradiation with a 450 nm LED and purification through flash column chromatography (SiO_2, EtOAc:*i*-Hex, gradient from 20:80 to 25:75 v:v).

E isomer

***R*f** (SiO_2, EtOAc:*i*-Hex, 15:85 v:v) = 0.35.

^{1}H NMR (600 MHz, CD_2Cl_2): δ / ppm = 7.77 (dd, ^3J = 7.7, ^4J = 1.1 Hz, 1H, H(C22)), 7.72 (dd, ^3J = 7.7 Hz, ^4J = 1.1 Hz, 1H, H(C20)), 7.16 (t, ^3J = 7.7 Hz, 1H, H(C21), 7.01 (d, ^3J = 8.9 Hz, 1H, H(C5)), 6.83 (d, ^3J = 8.9 Hz, 1H, H(C6)), 6.12 (br s, 1H, H15), 3.97 (s, 3H, H(C10)), 3.82 (s, 3H, H(C11)), 3.28 (d, ^2J = 16.8 Hz, 1H, [3/3′]-H), 2.88 (d, ^2J = 16.8 Hz, 1H, [3′/3]-H), 2.48 (sept, ^3J = 6.9 Hz, 1H, H(C12)), 1.08 (d, ^3J = 6.9 Hz, 3H, H(C13/C14)), 0.68 (d, ^3J = 6.9 Hz, 3H, H(C14/C13)).

^{13}C NMR (100 MHz, CD_2Cl_2): δ / ppm = 191.3 (C16), 167.6 (C1), 150.3 (C7), 149.9 (C4), 148.6 (C18), 138.4 (C8), 138.1 (C20), 134.4 (C23), 128.6 (C9/C17), 128.5 (C9/C17), 126.5 (C21), 125.9 (C22), 117.1 (C19), 116.8 (C5), 111.0 (C6), 91.9 (C2), 56.5 (C11), 55.4 (C10), 38.7 (C3), 36.5 (C12), 19.1(C13/C14), 17.5 (C13/C14).

HRMS (EI) calcd. for $[C_{22}H_{21}BrO_4S]^+$: 460.0343; found: 460.0347.

IR: $\tilde{\nu}$/cm^{-1} = 3850 (w), 3231 (w), 3076 (w), 3001 (m), 2962 (m), 2919 (m), 2872 (w), 2838 (w), 1634 (m), 1580 (m), 1560 (w), 1529 (s), 1490 (s), 1447 (m), 1435 (m), 1407 (m), 1379 (w), 1362 (w), 1334 (w), 1301 (w), 1265 (s) 1190 (m), 1164 (w), 1146 (w), 1133 (w), 1124 (w), 1105 (m), 1088 (m), 1077 (m), 1063 (m), 1039 (m), 1024 (w), 1007 (w), 990 (m), 965 (w) ,947 (w), 937 (w), 922 (w), 909 (w), 895 (w), 796 (w), 777 (w), 743 (s), 731 (m), 718 (w), 690 (m), 675 (m), 650 (m), 628 (m), 582 (w), 533 (w), 503 (w), 472 (w), 457 (w), 416 (w).

Melting point: 161 °C

Single crystals of the *E* isomer for structure determination by X-ray diffraction could be obtained by recrystallization from CH_2Cl_2:*n*-heptane (1:1).

Z isomer

***R*f** (SiO_2, EtOAc:*i*-Hex, 20:80 v:v) = 0.27.

^{1}H NMR (600 MHz, CD_2Cl_2): δ / ppm = 7.79 (dd, ^3J = 7.8, ^4J = 1.2 Hz, 1H, H(C22)), 7.69 (dd, ^3J = 7.8 Hz, ^4J = 1.2 Hz, 1H, H(C20)), 7.17 (t, ^3J = 7.8 Hz, 1H, H(C21), 6.92 (d, ^3J = 8.8 Hz, 1H, H(C5)), 6.77 (d, ^3J = 8.8 Hz, 1H, H(C6)), 3.81 (s, 3H, H(C11)), 3.80 (s, 3H, H(C10)), 3.39 (d, ^2J = 16 Hz, 1H, [3/3′]-H), 2.80 (d, ^2J = 16 Hz, 1H, [3/3′]-H), 2.51 (br s, 1H, H15), 2.08 (sept, ^3J = 6.8 Hz, 1H, H(C12)), 1.02 (d, ^3J = 6.8 Hz, 3H, H(C13/C14)), 0.79 (d, ^3J = 6.8 Hz, 3H, H(C13/C14)).

^{13}C NMR (100 MHz, CD_2Cl_2): δ / ppm = 186.9 (C16), 154.5 (C1), 152.8 (C7), 149.5 (C4), 147.1 (C18), 137.0 (C20), 134.5 (C23), 133.8 (C8), 128.7 (C9), 126.6 (C17), 126.5 (C21), 125.0 (C22), 117.9 (C19), 114.9 (C5), 110.4 (C6), 91.2 (C2), 56.7 (C10/C11), 55.9 (C10/C11), 41.3 (C3), 36.0 (C12), 18.2 (C13/C14), 18.1 (C13/C14).

HRMS (EI) calcd. for $[C_{22}H_{21}BrO_4S]^+$: 460.0343; found: 460.0347.l.

IR: $\tilde{\nu}$ = 3432 (w), 2957 (w), 2933 (w), 2895 (w), 2874 (w), 2831 (w), 1654 (s), 1582 (m), 1565 (w), 1494 (m), 1467 (w), 1450 (w), 1434 (w), 1408 (m), 1385 (w), 1366 (w), 1332 (w), 1300 (w), 1272 (w), 1256 (s), 1187 (w), 1157 (w), 1136 (w), 1101 (w), 1086 (s), 1020 (s), 975 (m), 954 (m), 910 (w), 855 (w), 804 (w), 791 (m), 772 (w), 757 (m), 739 (m), 721 (m), 705 (w), 683 (w), 662 (w), 648 (w), 630 (w), 585 (w), 650 (w), 545 (w), 501 (w), 492 (w), 468 (w), 458 (w), 450 (w), 441 (w), 430 (w), 422 (w), 408 (w).

Melting point: 173 °C

Single crystals of the *Z* isomer for structure determination by X-ray diffraction could be obtained by recrystallization from EtOAc:*n*-heptane (1:1).

6. NMR Spectra of Synthesized Compounds

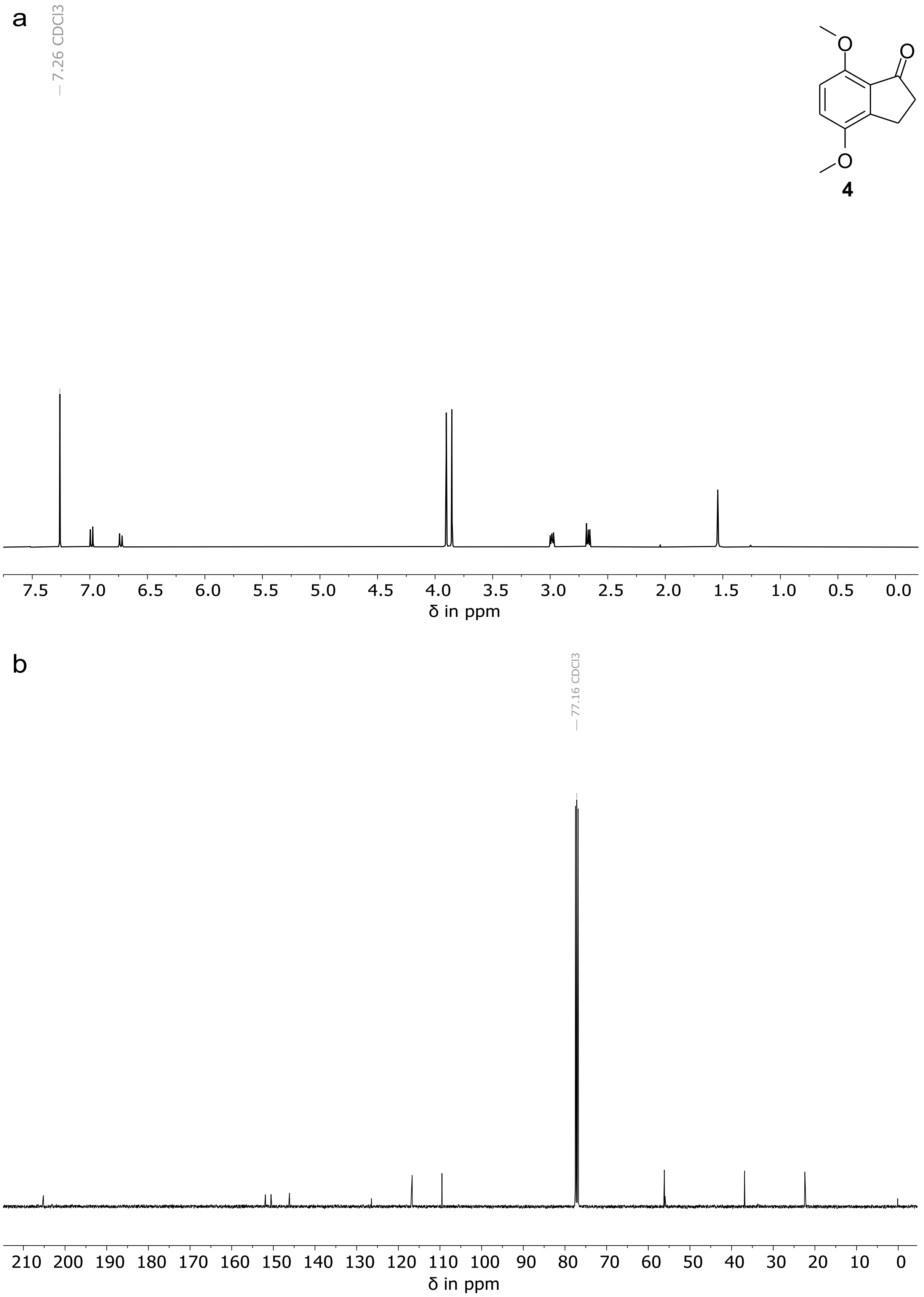

Supplementary Figure 20. ^{1}1D NMR spectra of **4**. a) ^{1}H NMR spectrum ($CDCl_3$, 400 MHz, 25 °C). b) ^{13}C NMR spectrum ($CDCl_3$, 100 MHz, 25 °C).

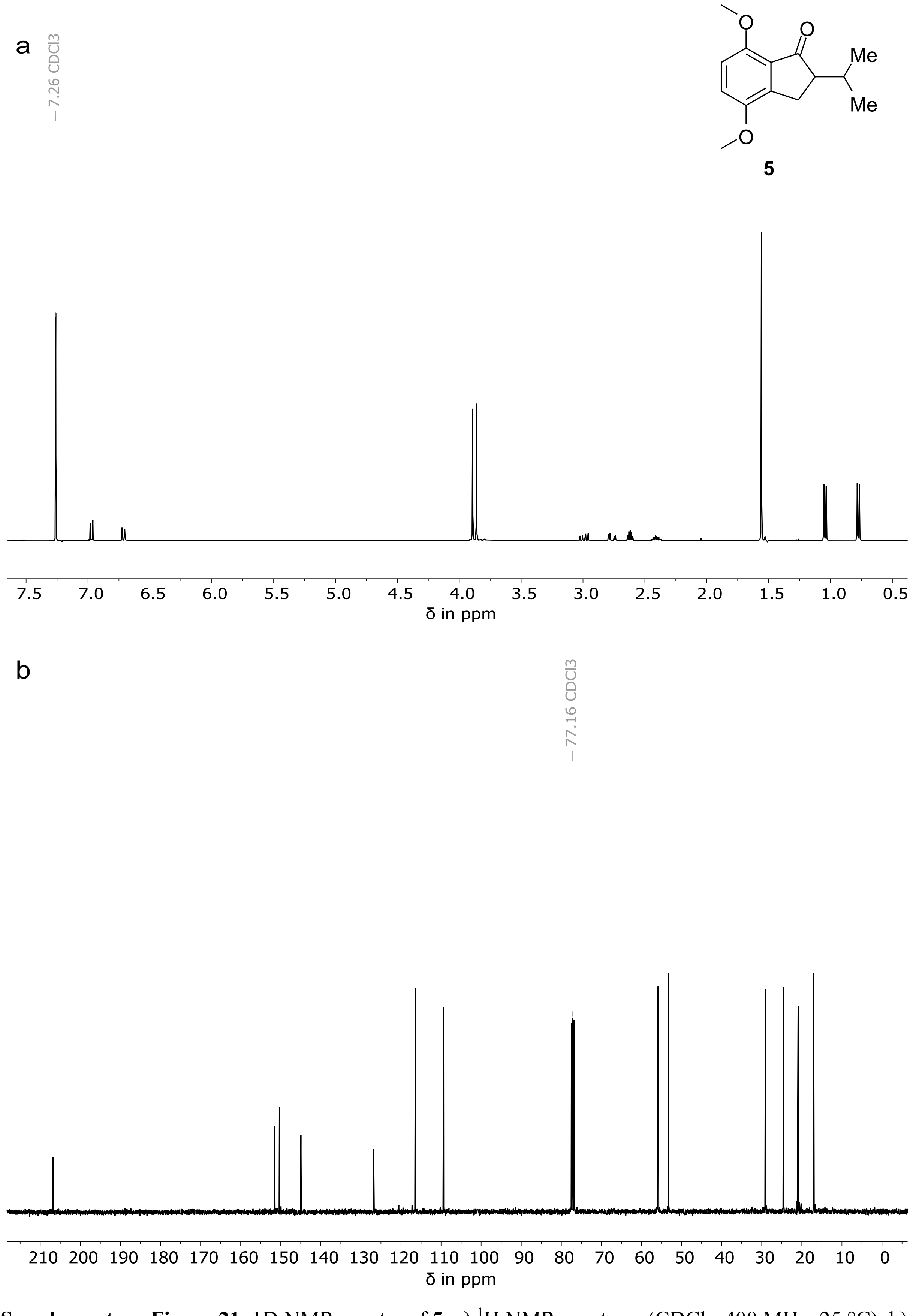

Supplementary Figure 21. 1D NMR spectra of **5**. a) ^{1}H NMR spectrum ($CDCl_3$, 400 MHz, 25 °C). b) ^{13}C NMR spectrum ($CDCl_3$, 100 MHz, 25 °C).

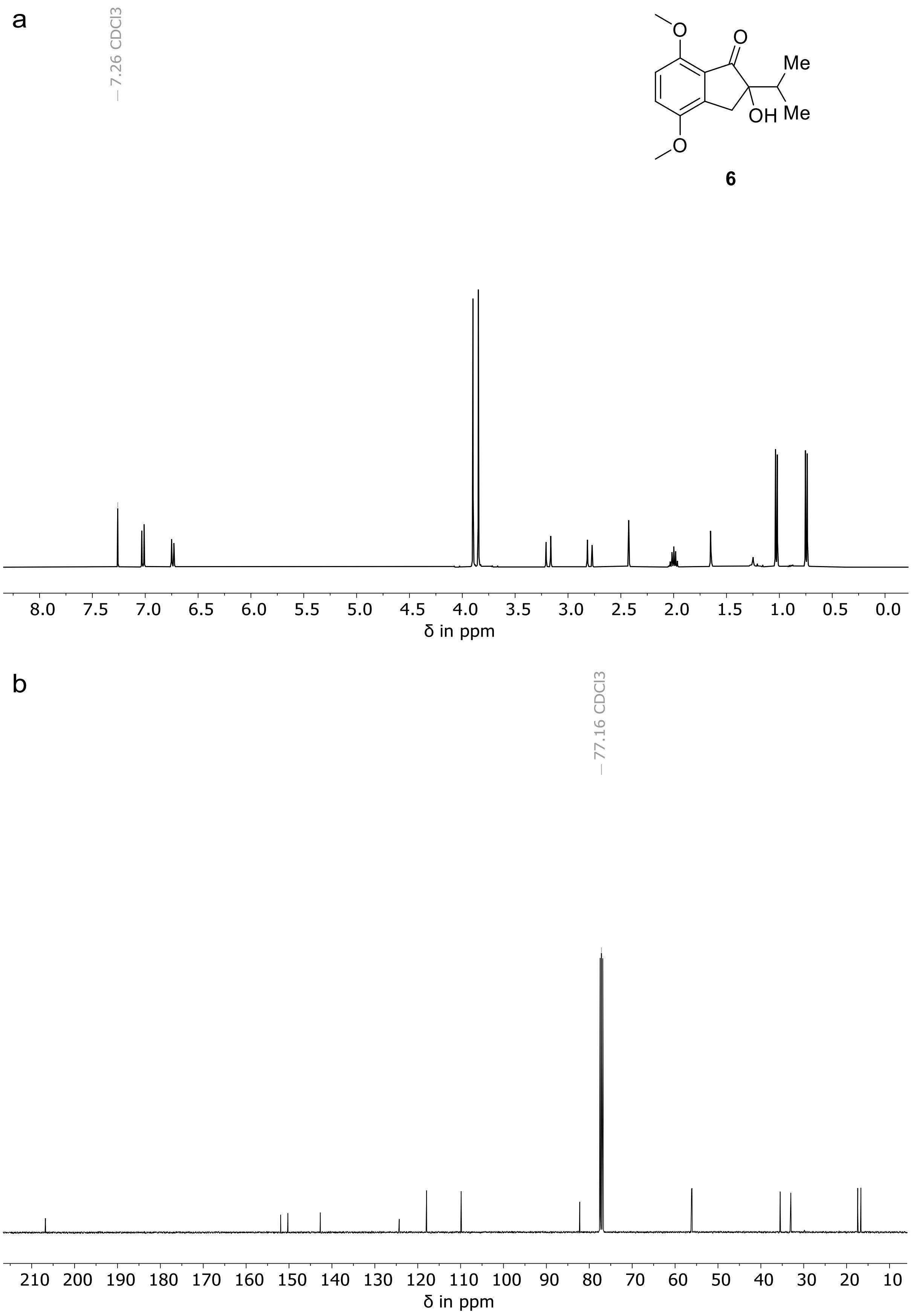

Supplementary Figure 22. 1D NMR spectra of **6**. a) ^{1}H NMR spectrum ($CDCl_3$, 400 MHz, 25 °C). b) ^{13}C NMR spectrum ($CDCl_3$, 100 MHz, 25 °C).

a

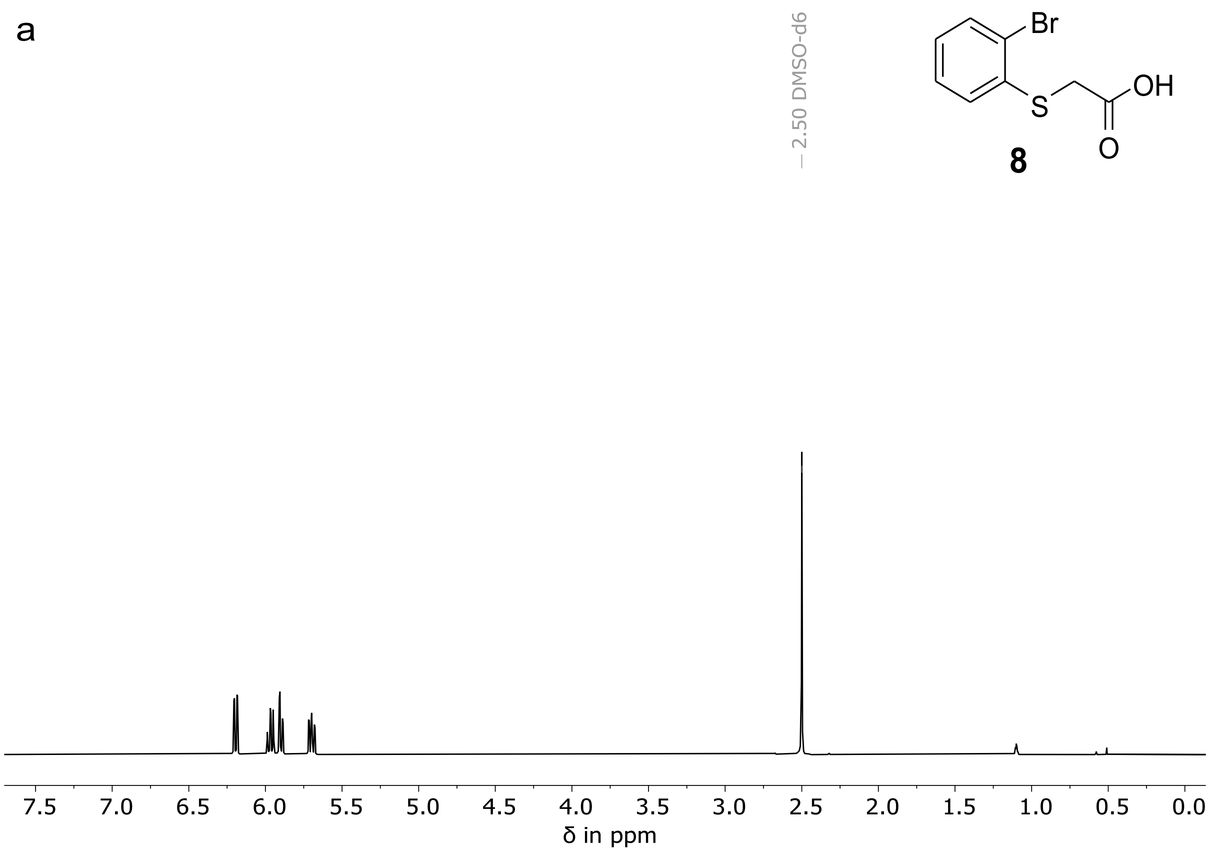

b

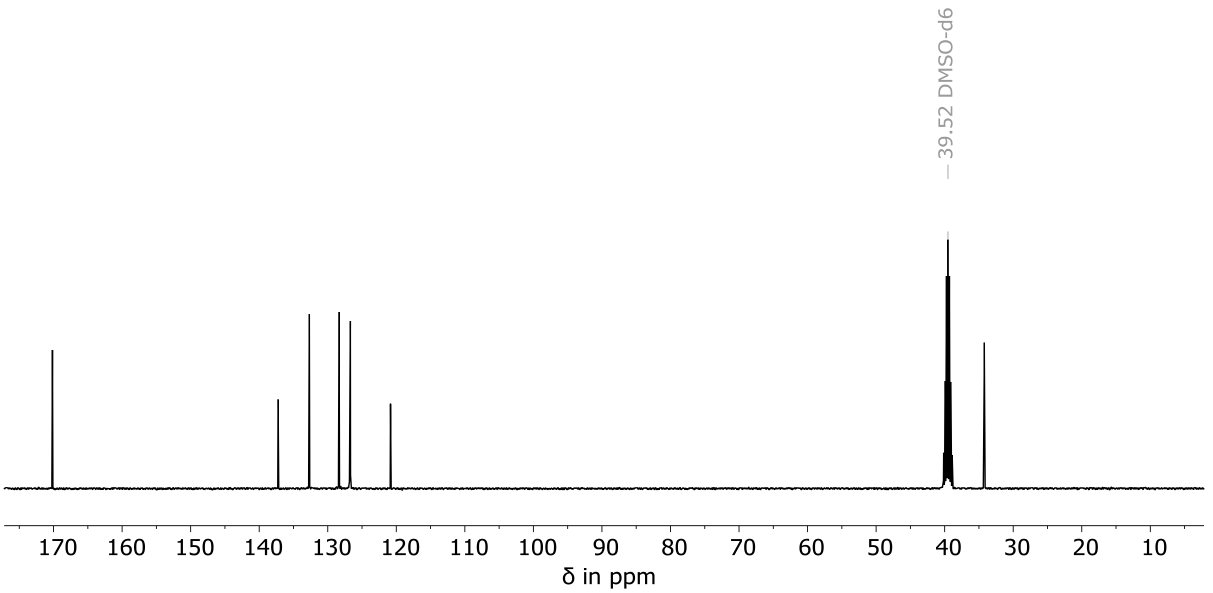

Supplementary Figure 23. 1D NMR spectra of **8**. a) ^{1}H NMR spectrum DMSO-d_6, 400 MHz, 25 °C) b) ^{13}C NMR spectrum (DMSO-d_6, 100 MHz, 25 °C).

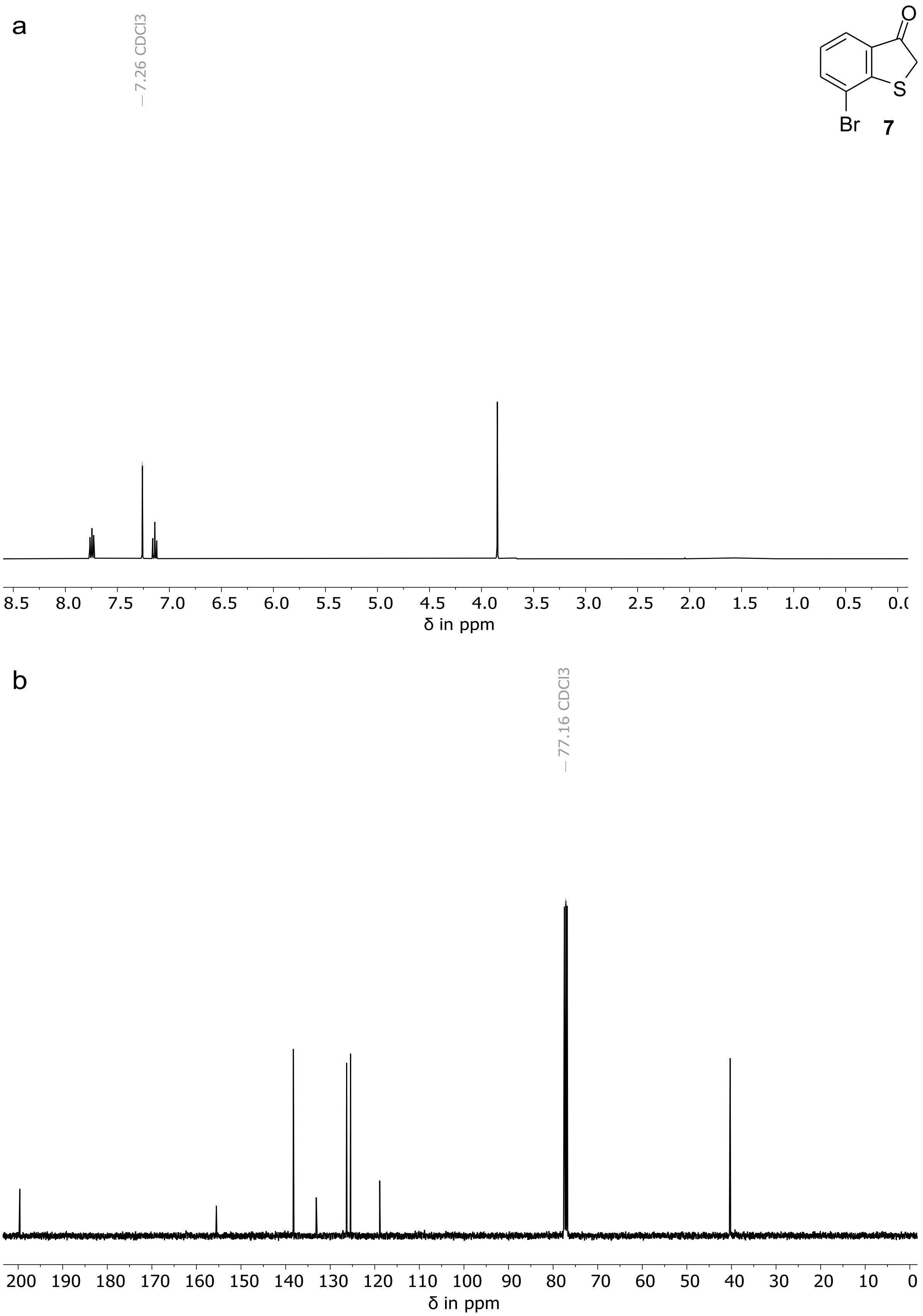

Supplementary Figure 24. 1D NMR spectra of **7**. a) ^{1}H NMR spectrum ($CDCl_3$, 400 MHz, 25 °C). b) ^{13}C NMR spectrum ($CDCl_3$, 100 MHz, 25 °C).

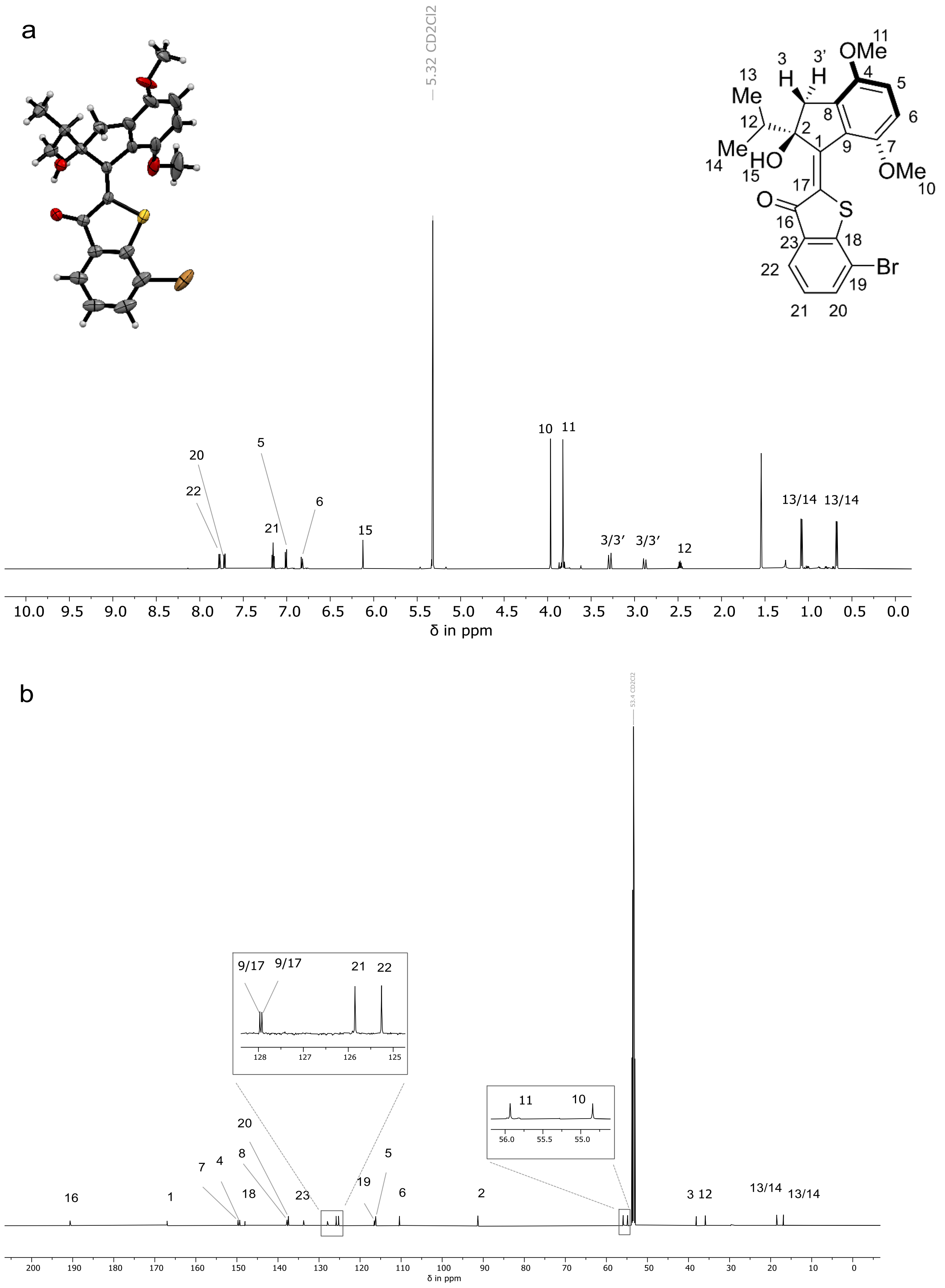

Supplementary Figure 25. 1D NMR spectra of *E*-configured stable isomer (**A**) of motor **1**. a) ^{1}H NMR spectrum (CD_2Cl_2, 600 MHz, 25 °C). b) ^{13}C NMR spectrum (CD_2Cl_2, 151 MHz, 25 °C).

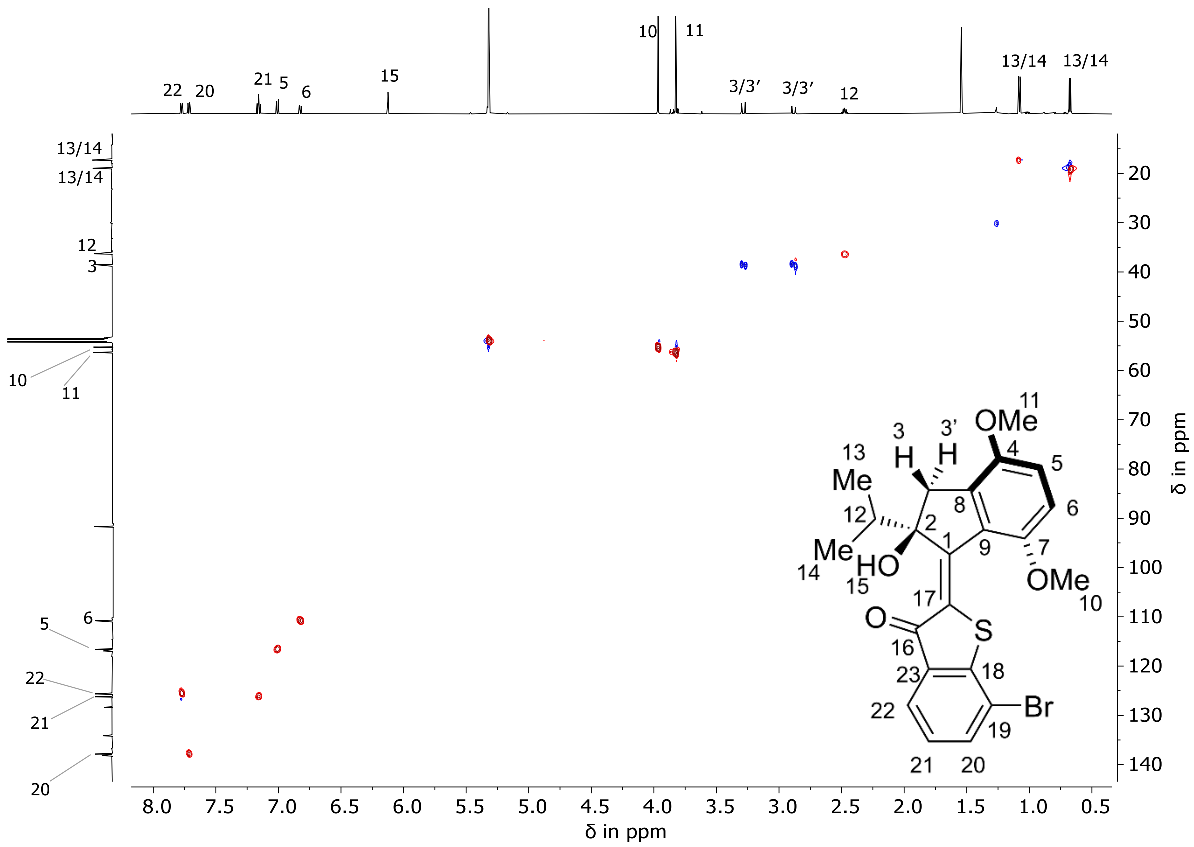

Supplementary Figure 26. ^{1}H-^{13}C NMR HSQC spectrum (CD_2Cl_2, 600 MHz, 25 °C) of *E*-configured stable isomer **A** of motor **1** and related peak assignments to the molecular structure.

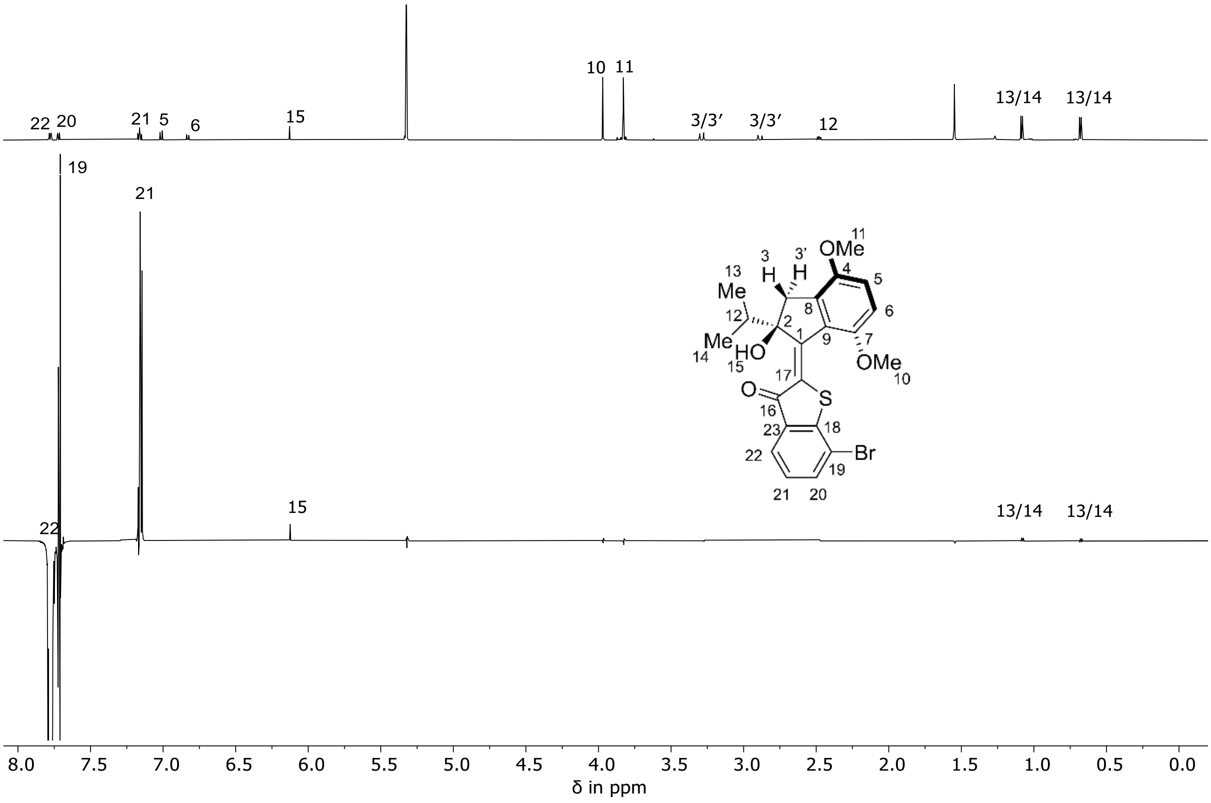

Supplementary Figure 27. 1D NOE experiment (CD_2Cl_2, 600 MHz, 25 °C) of stable isomer **A** of motor **1** confirming *E* configuration. Double bond configuration was assigned by the couplings of aromatic proton H-C22 with H-15 (OH) and protons H-C13 and H-C14.

a

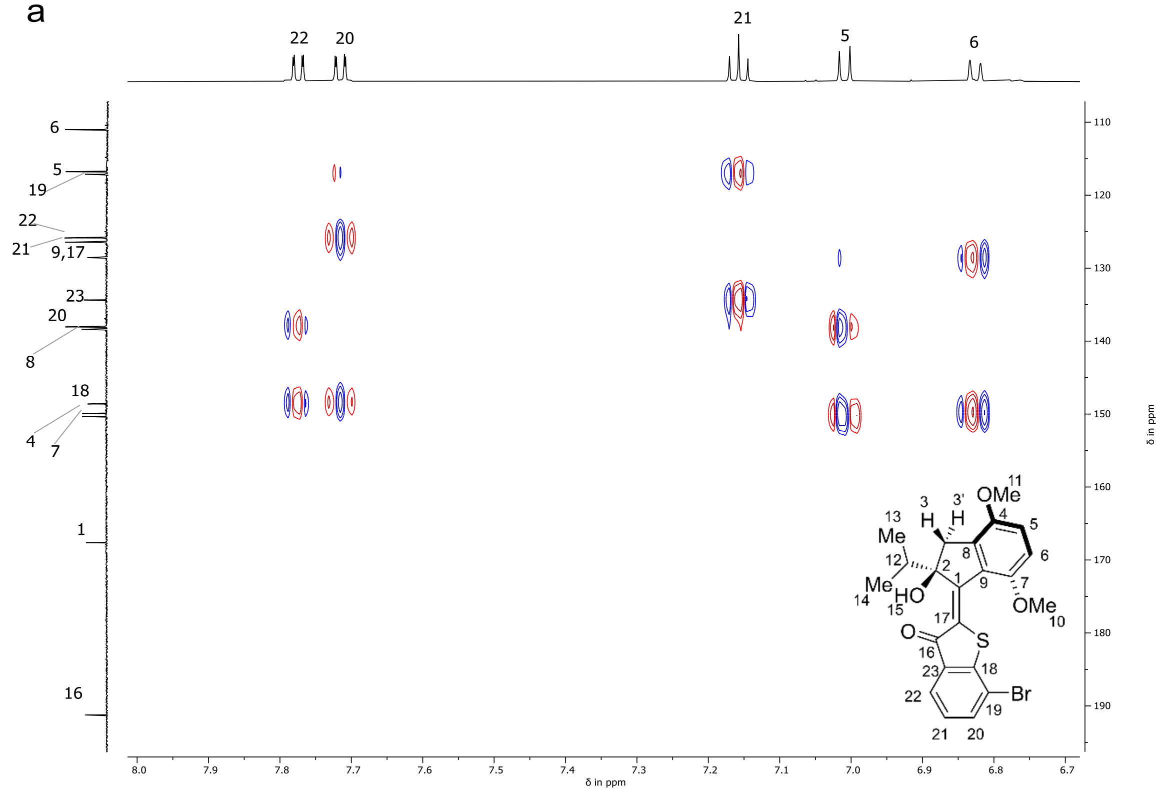

b

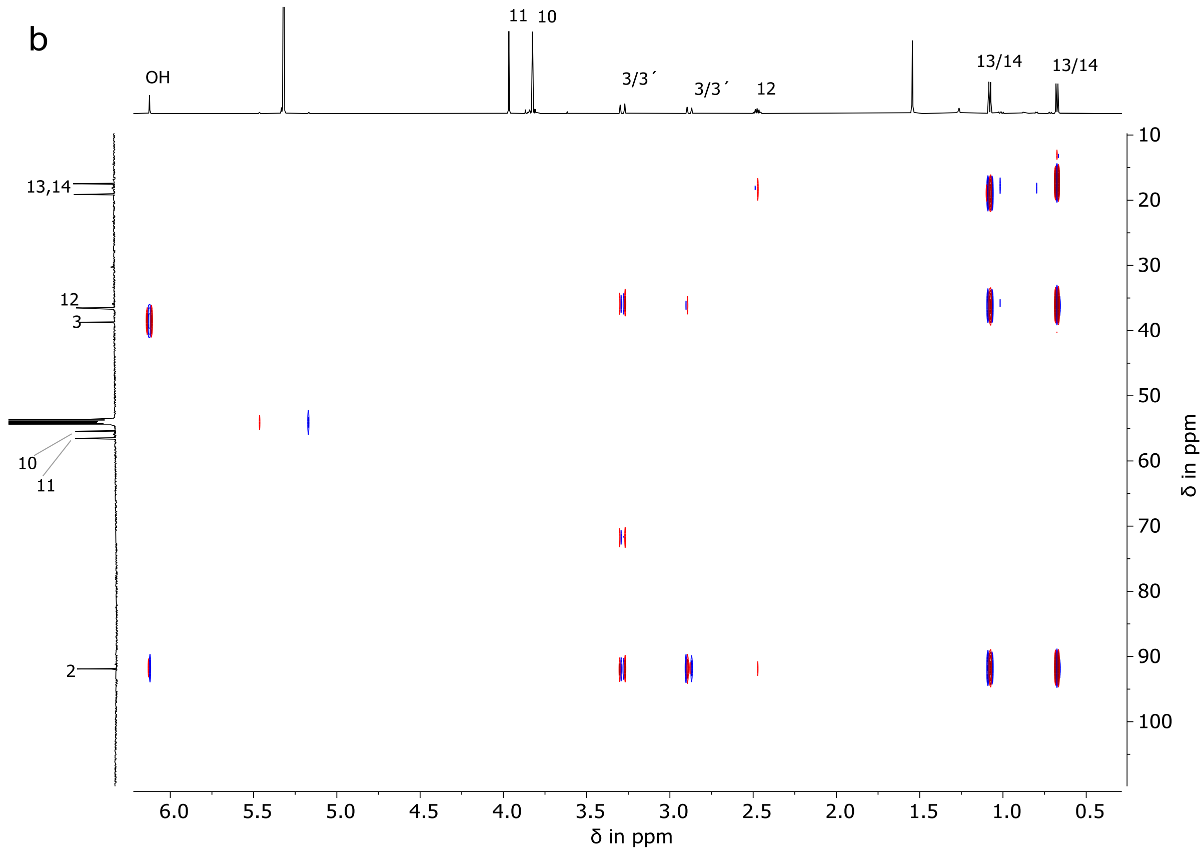

Supplementary Figure 28. ^{1}H-^{13}C NMR HMBC spectrum (CD_2Cl_2, 600 MHz, 25 °C) of *E*-configured stable isomer (**A**) of motor **1** and related peak assignments of the molecular structure. a) Enlarged aromatic-aromatic region of the spectrum. b) Enlarged aliphatic-aliphatic region of the spectrum.

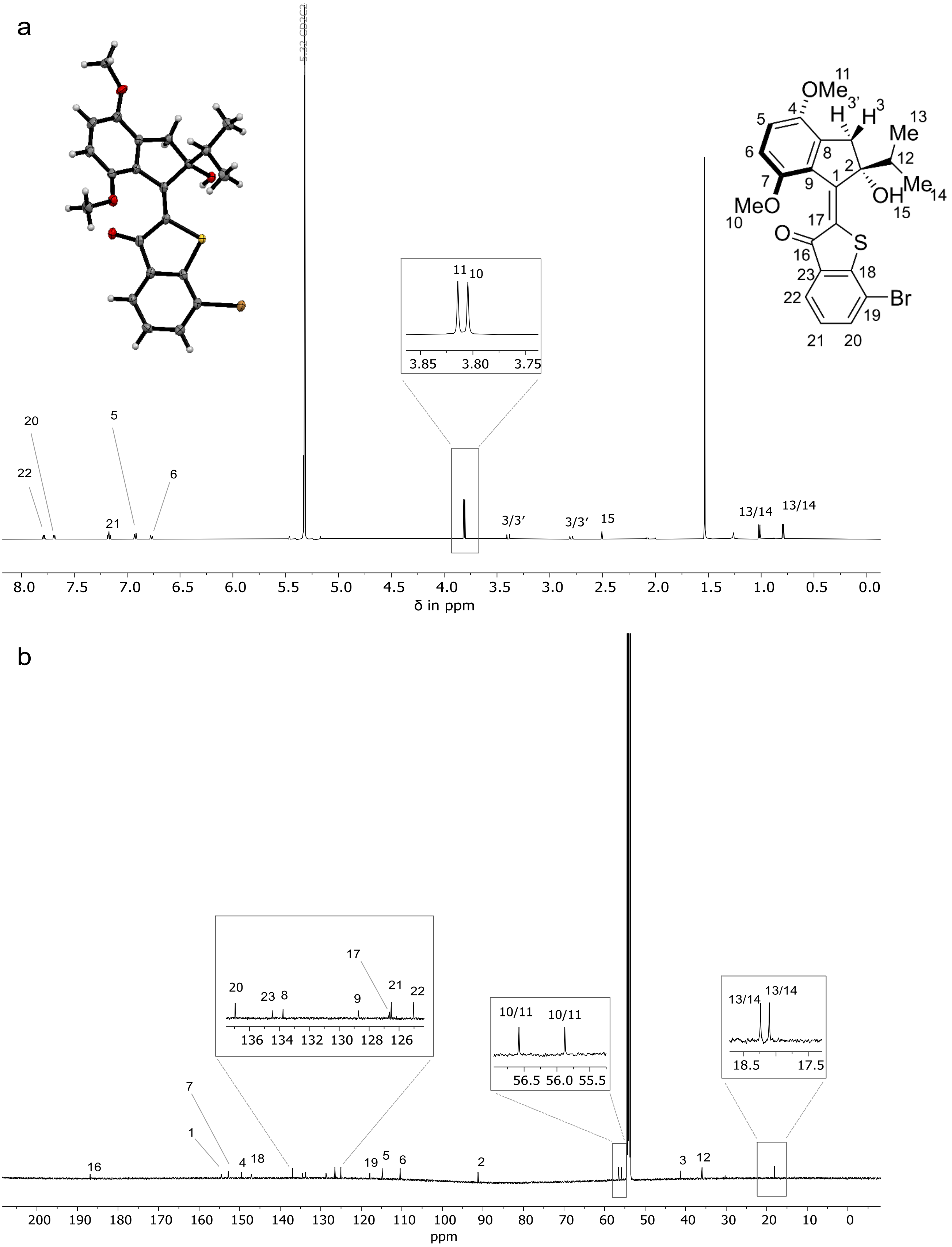

Supplementary Figure 29. 1D NMR spectra of *Z*-configured isomer (**C**) of motor **1**. a) ^{1}H NMR spectrum (CD_2Cl_2, 600 MHz, 25 °C). b) ^{13}C NMR spectrum (CD_2Cl_2, 151 MHz, 25 °C).

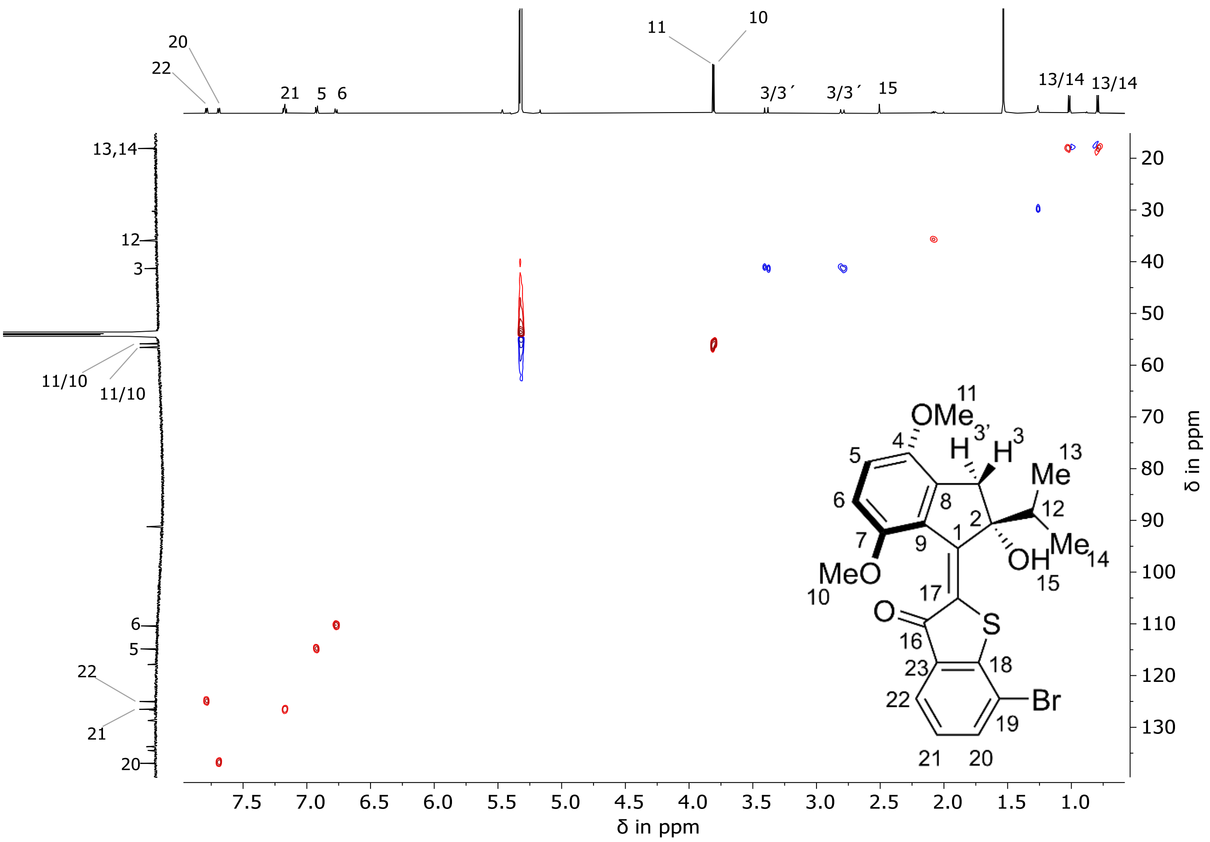

Supplementary Figure 30. ^{1}H-^{13}C NMR HSQC spectrum (CD_2Cl_2, 600 MHz, 25 °C) of *Z*-configured isomer **C** of motor **1** and related peak assignments to the molecular structure.

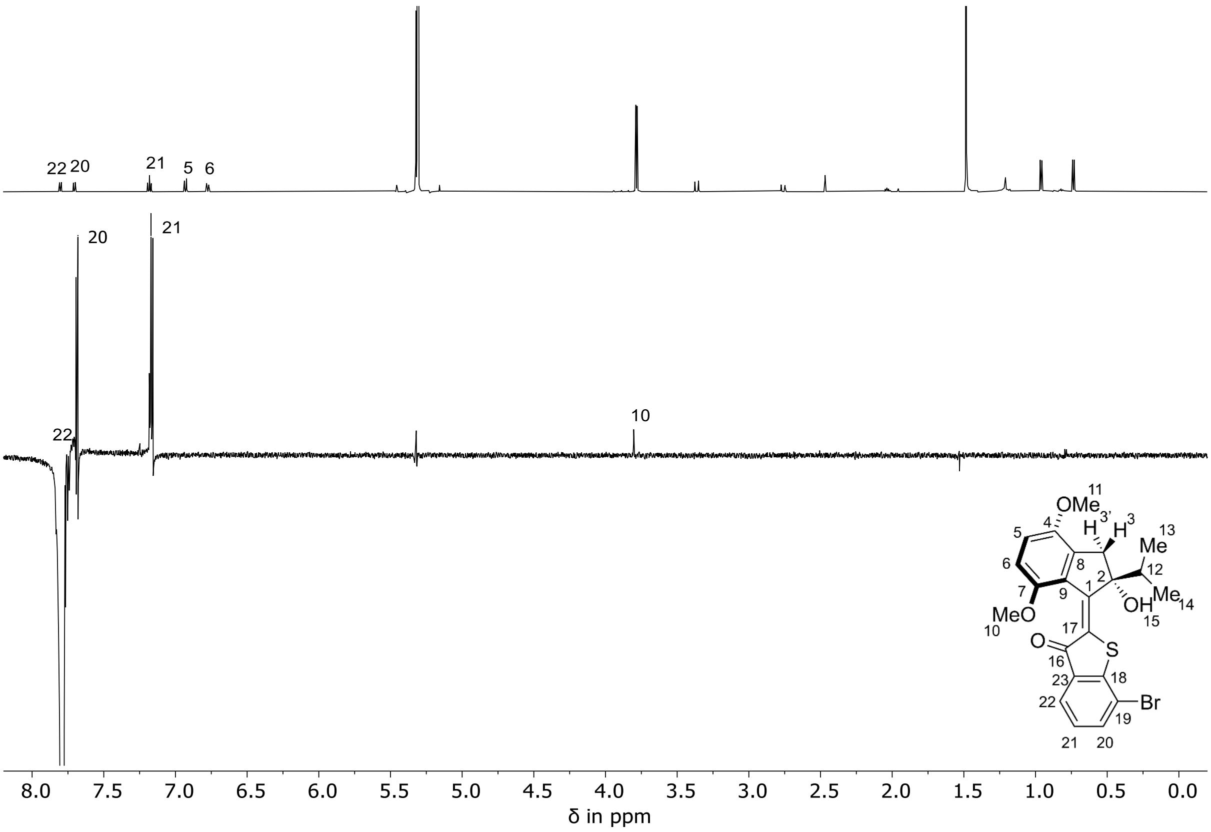

Supplementary Figure 31. 1D NOE experiment (CD_2Cl_2, 600 MHz, 25 °C) of isomer **C** of motor **1** confirming *Z* configuration. Double bond configuration was assigned by the couplings of aromatic proton H-C22 with and proton H-C10.

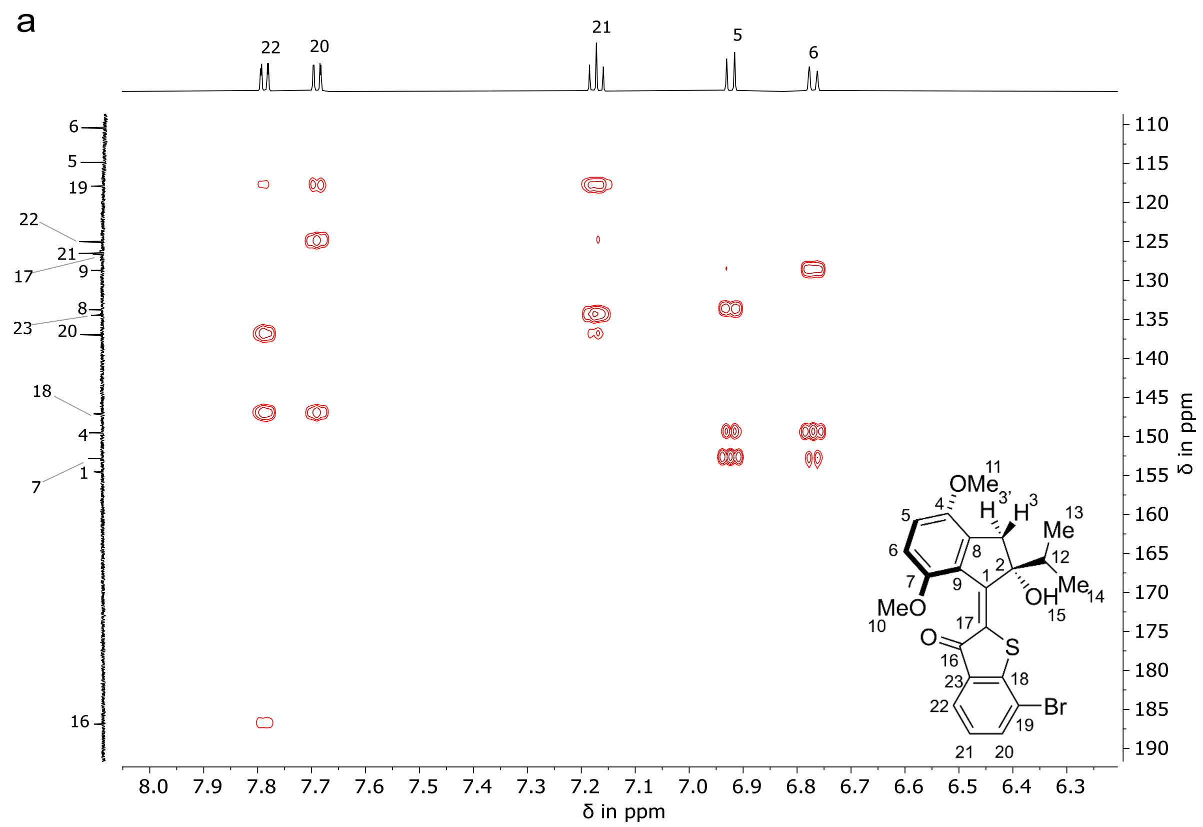

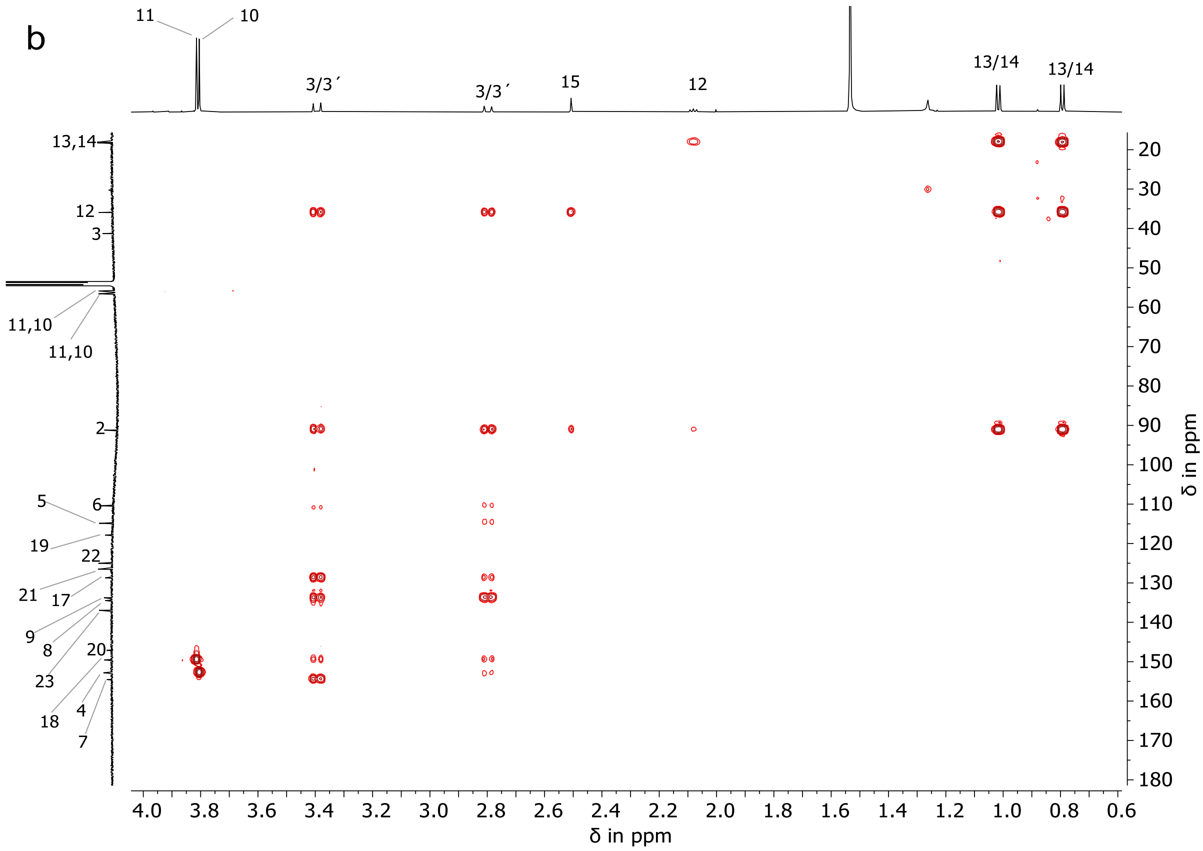

Supplementary Figure 32. ^{1}H-^{13}C NMR HMBC spectrum (CD_2Cl_2, 600 MHz, 25 °C) of *Z*-configured isomer (**C**) of motor **1** and related peak assignments of the molecular structure. a) Enlarged aromatic-aromatic region of the spectrum b) Enlarged aliphatic-aliphatic region of the spectrum.

7. Enantiomeric Resolution

The enantiomers of stable isomers **A-1** and **C-1** were separated through HPLC using a semi-preparative chiral stationary phase CHIRALPAK® ID (Daicel Chemical Industries) column at 30 °C. The results and chromatograms are shown in the Supplementary Fig. 33 and 34.

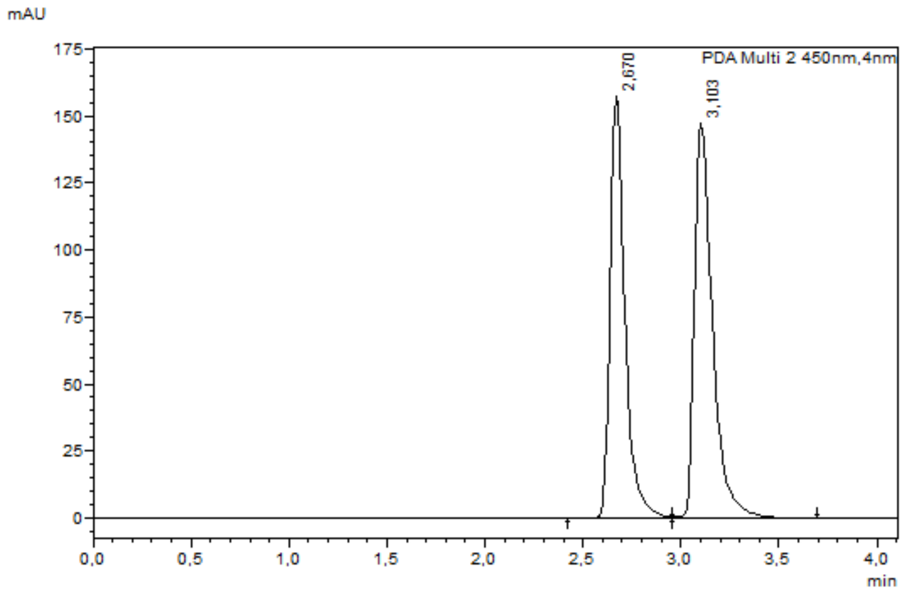

Supplementary Figure 33. Chromatogram for the enantiomeric resolution of stable isomer **A-1**. Conditions: EtOAc:*i*-Hex 30:70, v:v. Flow rate: 10 mL/min. Retention times: $t_{(\text{frI})}$ = 2.67 min and $t_{(\text{frII})}$ = 3.10 min.

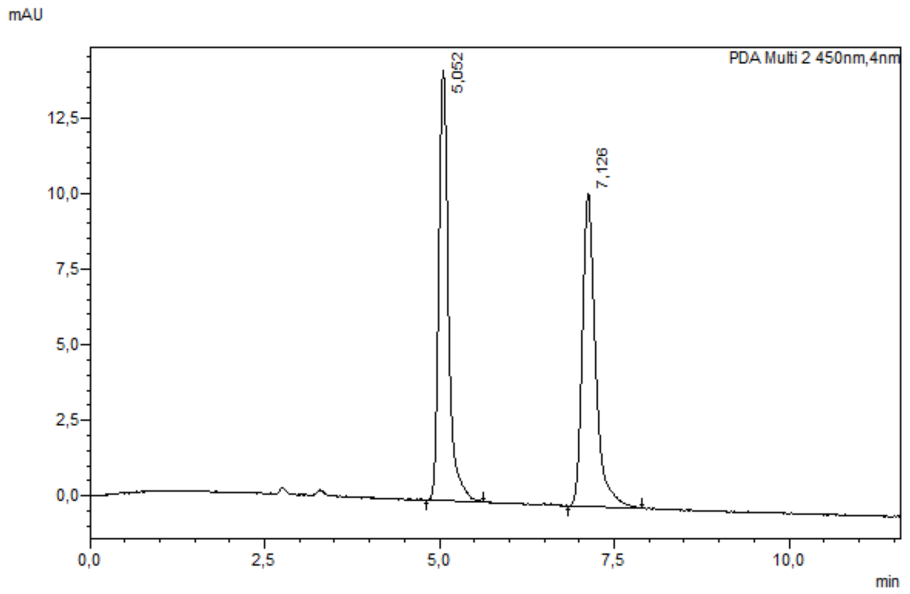

Supplementary Figure 34. Chromatogram for the enantiomeric resolution of stable isomer **C-1**. Conditions: EtOAc:*i*-Hex 30:70, v:v. Flow rate: 10 mL/min. Retention times: $t_{(\text{frI})}$ = 5.05 min and $t_{(\text{frII})}$ = 7.13 min.

8. Temperature and Irradiation-Dependent Behavior of Motor

8.1 Elevated Temperature Behavior

As stated above, the *E*-configured **A** isomer is the thermodynamically most stable form of motor **1**. At elevated temperatures in the dark, the **C-1** isomer converts to **A-1** isomer quantitatively. Supplementary Fig. 35 shows the ^{1}H NMR spectra of **1** during the heating experiment at 100 °C in toluene-d_8 and the corresponding kinetic plots can be found in Supplementary Fig. 36.

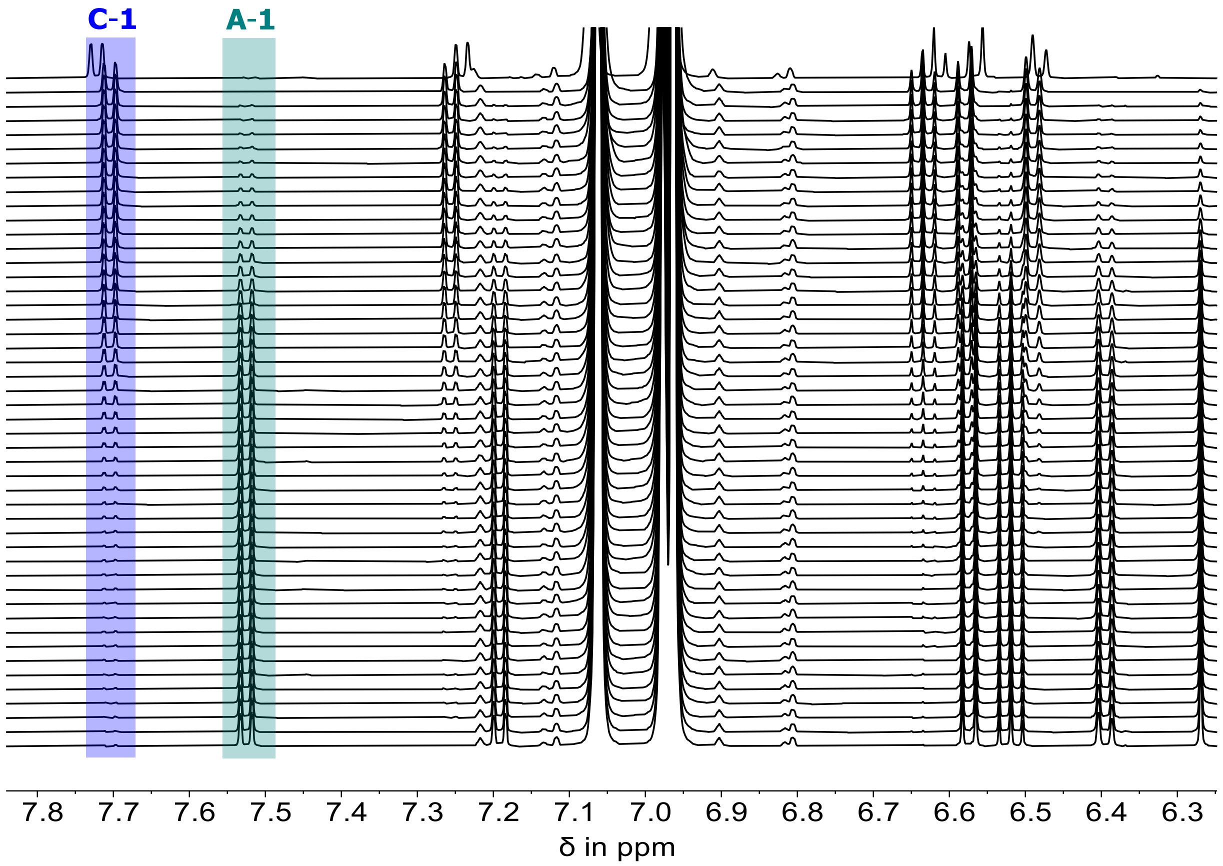

Supplementary Figure 35. Stacked ^{1}H NMR spectra (toluene-d_8, 500 MHz) recorded during heating at 100 °C of *Z*-configured isomer **C-1** (blue), which leads to quantitative conversion to *E*-configured isomer **A-1** (green). The initial spectrum was measured at 80 °C and the remaining spectra were taken at 100 °C.

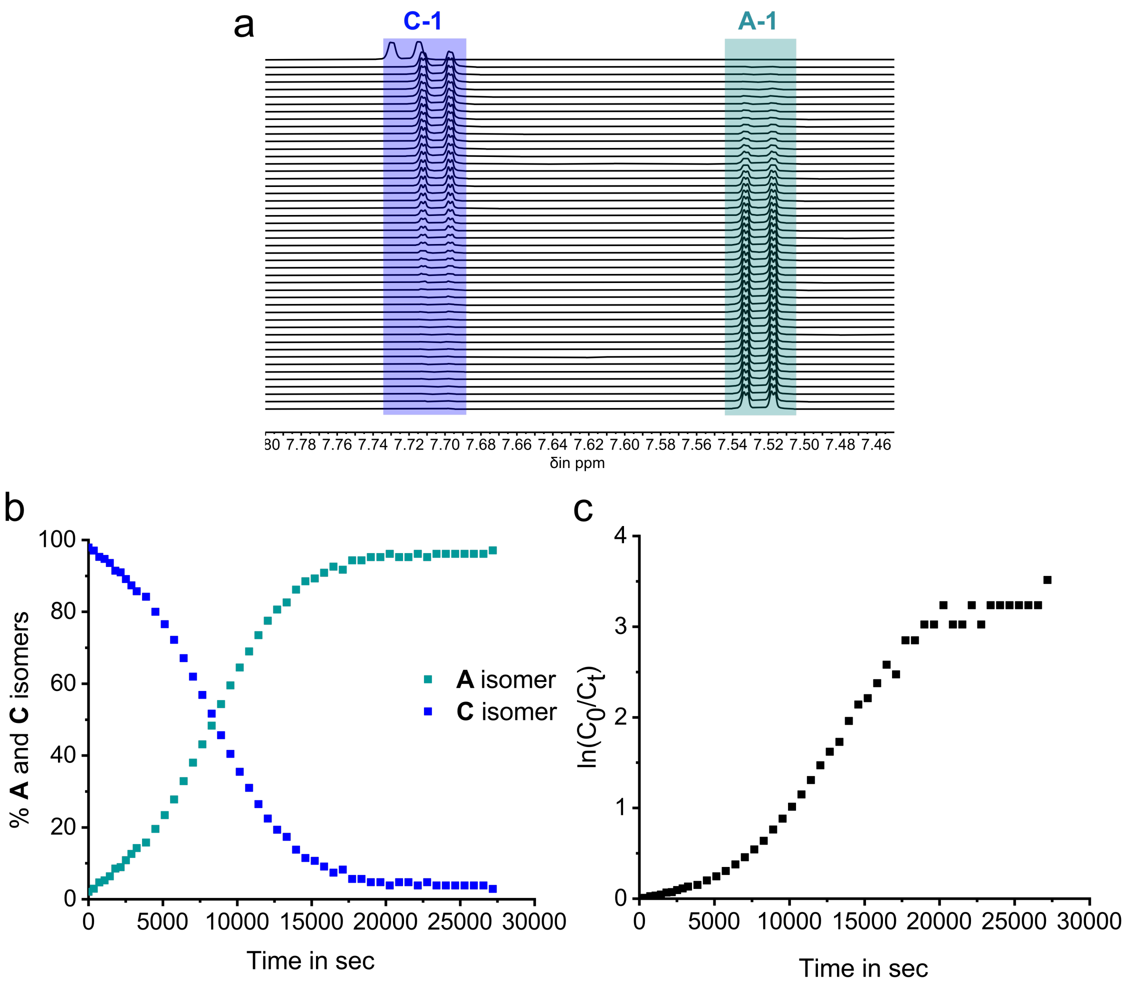

Supplementary Figure 36. Kinetic analysis of the thermal conversion of **C-1** to **A-1** performed at 100°C in the dark. **a)** ^{1}H NMR spectra (toluene-d_8, 500 MHz) with signals corresponding to the aromatic protons of **C-1** (blue) and **A-1** (green) depicted for demonstration of the gradual conversion. The initial spectrum was recorded at 80 °C and the remaining spectra were taken in 6 min or 10 min time intervals at 100 °C over the course of 453 min of heating. **b)** Decrease of **C-1** isomer and increase of **A-1** isomer are shown over time. Data points were obtained from integration of indicative NMR signals. At the starting point 98% **C-1** isomer was present. After completion of the heating experiment, **A-1** isomer is enriched up to 96%, which can be translated to an energy difference of at least 2.36 kcal/mol between the two isomers (an equilibrium of $K = 96/4$ between **A-1**/**C-1**, according to $\Delta G = \text{RT}\ln K$). **c)** First-order kinetic analysis on **C-1** to **A-1** conversion. As the obtained plot is not linear, the isomerization process does not obey first-order kinetics and follows a more complex mechanism.

According to Supplementary Figures 35 and 36, the **A-1**:**C-1** isomeric ratio in thermal equilibrium at 100 °C is 96:4. This equilibrium reflects the minimum relative energy difference –ΔG between the two isomeric states as calculated by Equation 1:

$$-\Delta G = R \cdot T \cdot \ln K \qquad (1)$$

with K = equilibrium constant = [**A**]/[**C**]

R = ideal gas constant = (8.314 J K^{-1} mol^{-1})

T = temperature in K

After applying Equation 1 a minimum energy difference between the **C-1** and **A-1** isomers of 2.36 kcal/mol was found. This process does not follow a first-order mechanism as plotting time vs. $\ln(C_0/C_t)$ does not result in a linear correlation.

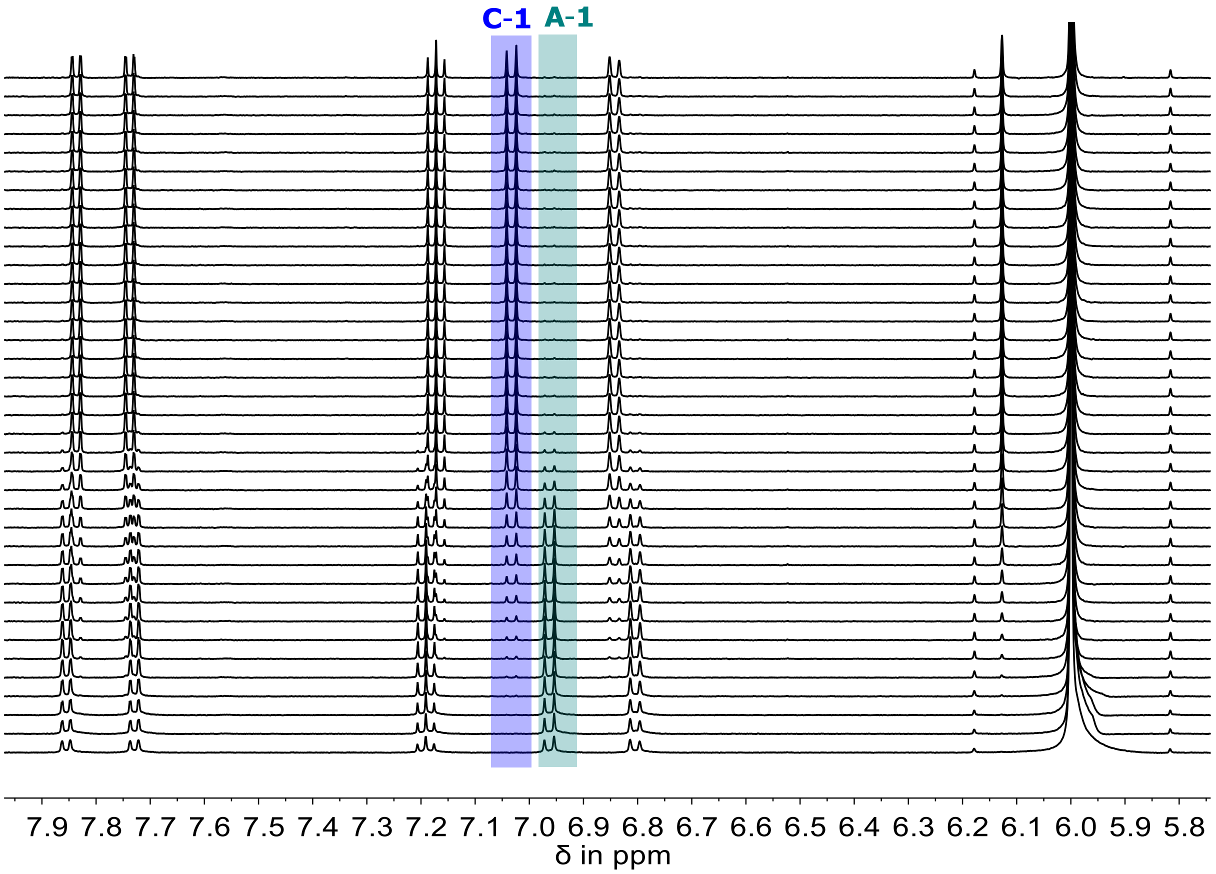

Supplementary Figure 37. Stacked ^{1}H NMR spectra (TCE-d_2, 500 MHz, 100 ºC) recorded during heating at 100 °C of *Z*-configured isomer **C-1** (blue), which leads to quantitative conversion to *E*-configured isomer **A-1** (green).

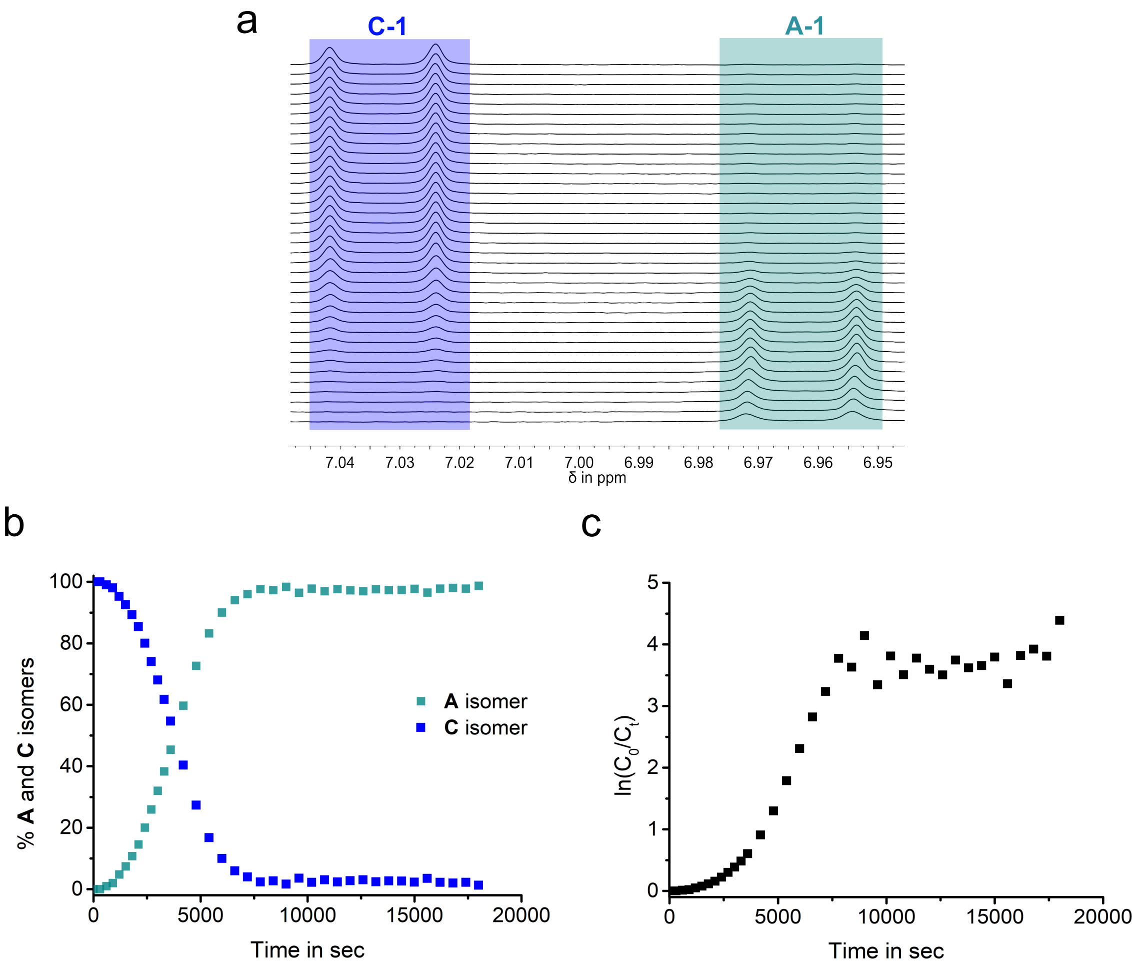

Supplementary Figure 38. Kinetic analysis of the thermal conversion of **C-1** to **A-1** performed at 100°C in the dark. **a)** ^{1}H NMR spectra (TCE-d_2, 500 MHz) with signals corresponding to the aromatic protons of **C-1** (blue) and **A-1** (green) depicted for demonstration of the gradual conversion. The spectra were recorded every 5, 10, or 60 min at 100 °C over the course of 420 min of heating. **b)** Decrease of **C-1** isomer and increase of **A-1** isomer are shown over time. Data points were obtained from integration of indicative NMR signals. Initially, 100% **C-1** isomer was present. After completion of the heating experiment, **A-1** isomer is enriched quantitatively, which can be translated to an energy difference of at least 2.89 kcal/mol between the two isomers (from a conservatively assumed equilibrium of K = 98/2 between **A-1**/**C-1**, according to ΔG = RTln$K$). **c)** First-order kinetic analysis on **C-1** to **A-1** conversion. As the obtained plot is not linear, the isomerization process does not obey first-order kinetics and follows a more complex mechanism.

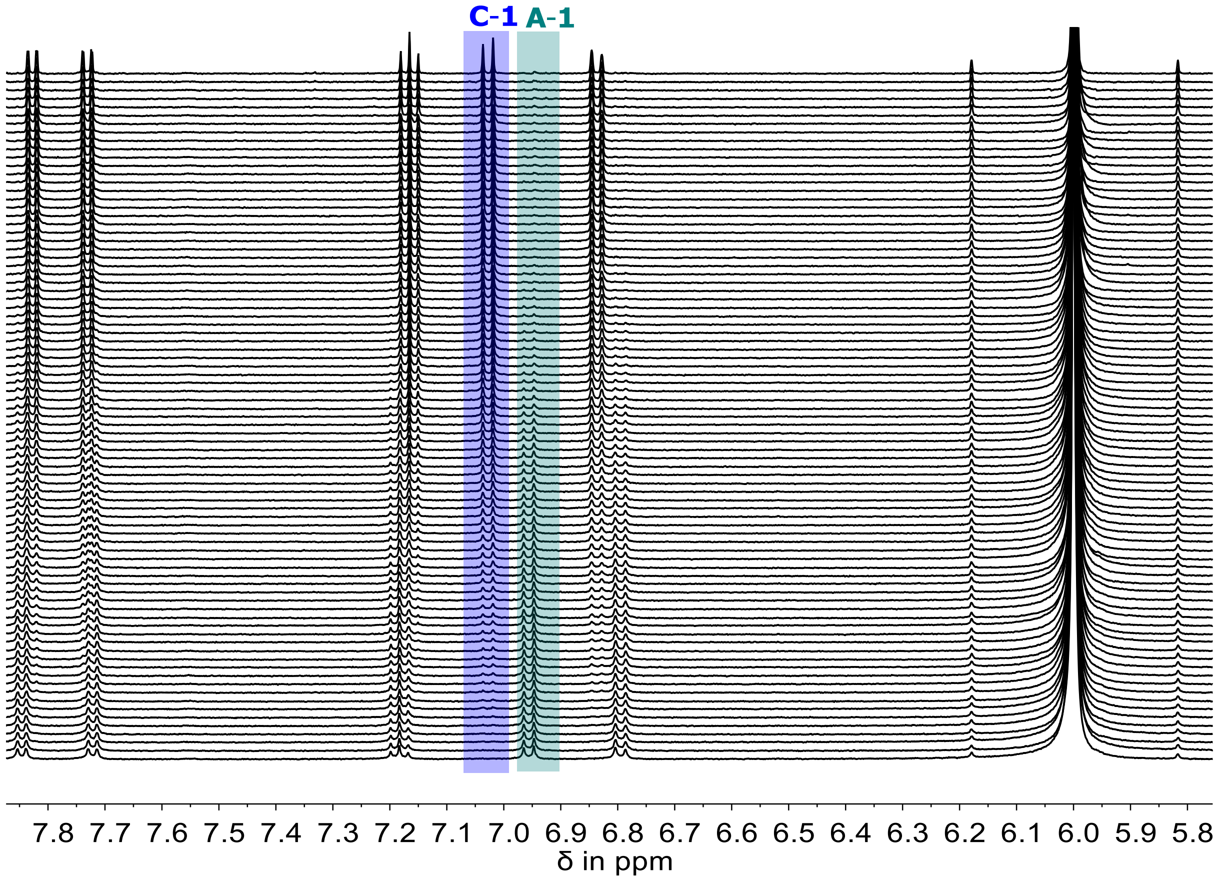

Supplementary Figure 39. Stacked ^{1}H NMR spectra (TCE-d_2 and 2 drops of D_2O, 500 MHz, 100 ºC) recorded during heating at 100 °C of *Z*-configured isomer **C-1** (blue), which leads to quantitative conversion to *E*-configured isomer **A-1** (green).

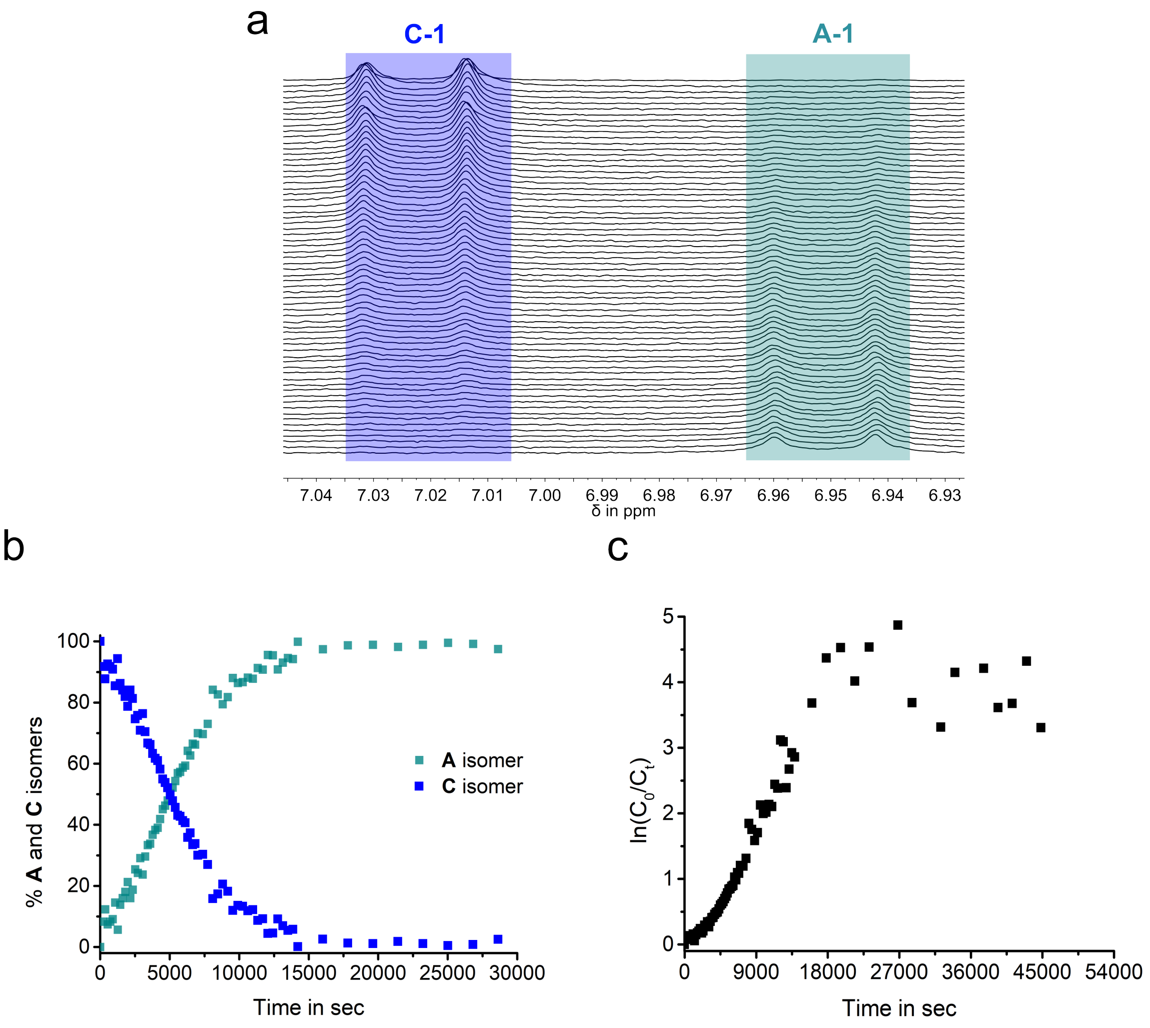

Supplementary Figure 40. Kinetic analysis of the thermal conversion of **C-1** to **A-1** performed at 100°C in the dark. **a)** ^{1}H NMR spectra (TCE-d_2 and 2 drops of D_2O, 500 MHz) with signals corresponding to the aromatic protons of **C-1** (blue) and **A-1** (green) depicted for demonstration of the gradual conversion. The spectra were recorded every 3, 10, or 30 min at 100 °C over the course of 837 min heating. **b)** Decrease of **C-1** isomer and increase of **A-1** isomer are shown over time. Data points were obtained from integration of indicative NMR signals. Initially, 100% **C-1** isomer are present. After completion of the heating experiment, **A-1** isomer is enriched up to 99.6%, which can be translated to an energy difference of at least 4.10 kcal/mol between the two isomers (from the equilibrium of K = 99.6/0.4 between **A-1**/**C-1**, according to ΔG = RTln$K$). **c)** First-order kinetic analysis on **C-1** to **A-1** conversion. As the obtained plot is not linear, the isomerization process does not obey first-order kinetics and follows a more complex mechanism.

The kinetic analysis on **C-1** to **A-1** isomerization was carried out in solvents of different polarity and the related experimental data are depicted in Supplementary Figures 37– 40. In TCE-d_2, using both the value of K = 98/2 for the equilibrium constant at 100 °C and Equation 1, 2.89 kcal/mol are obtained as minimum energy difference between the **C-1** and **A-1** isomers. To reveal the influence of water in this isomerization process of motor **1**, the kinetics were also studied in TCE-d_2 saturated in D_2O at 100 ºC. A minimum value of K = 99.6/0.4 was used

although quantitative conversion was observed in the ^{1}H NMR measurements to account for possible small amounts of residual **C-1** isomer that the NMR experiment would not be able to discern. The corresponding energy difference was found to be significantly higher with 4.10 kcal/mol. In line with the experimental data obtained in toluene-*d*$_8$*,* time vs. $\ln(C_0/C_t)$ plots neither in TCE-*d*$_2$ nor D_2O-saturated TCE-*d*$_2$ resulted in a linear relationship, meaning that thermal *Z* to *E* isomerization of motor **1** follows a higher-order mechanism. The presence of inter- and/or intramolecular hydrogen bonding in this molecular motor setup might be the reason for such complex kinetics. Upon addition of D_2O the kinetics are indeed slowed down, showing a noticeable effect in agreement with the observation that the evaporation of water during heating can lead to an acceleration of the kinetics. Note, however that **C-1** to **A-1** thermal isomerization does not take place upon heating to 55 ºC in $CDCl_3$ for 76 min, or in THF-*d*$_8$ at 60 ºC for 168 min. Also, in DMSO-*d*$_6$, **A-1** can be only enriched by 2% after heating to 100 ºC for a total of 960 min. Thus, thermal double bond rotation is not taking place at ambient temperatures and does not interfere with the motor operation under irradiation. Because of the non-linear kinetics we report half-lives for the conversions instead of rate constants. The data related to all these kinetic experiments in different solvents are detailed in Supplementary Table 7.

Supplementary Table 7. Kinetic analysis data for **C-1** to **A-1** isomerization in different solvents. (a) The sample from the kinetics in $CDCl_3$ was used. (b) Time required to reach equilibrium. (c) The same concentration was employed and kept as 1.65 x 10^{-3} M.

Entry	Initial Ratio (A:C)	Solvent	Temperature / ºC	Time / min	$t_{1/2}$ / min	Final Ratio (A:C)	ΔE / kcal/mol
**1**	29:71	THF-*d*$_8$	60	168	>168	No change	-
**2**	0:100	$CDCl_3$	58	76	>76	No change	-
**3**	3:97	Toluene-*d*$_8$	100	318.5$^{(b)}$	128.5	96:4	2.36
**4**	0:100	TCE-*d*$_2$$^{(c)}$	100	150$^{(b)}$	60	98:2	2.89
**5**	0:100	TCE-*d*$_2$ + 2 drops of $D_2O^{(c)}$	100	417$^{(b)}$	84	99.6:0.4	4.10
**6**	2:98	DMSO-*d*$_6$$^{(a)(c)}$	100	960	>960	4:96	-

9. *In situ* NMR Irradiation Experiments at Low Temperature

9.1 Motor Function and Thermal Behavior of HTI-1

In an NMR tube, *E*-configured isomer **A** of motor **1** was dissolved in a CD_2Cl_2:CS_2 = 4:1 mixture and ^1H NMR spectra were measured at −105 °C during extended irradiation with a high-power 450 nm LED. Supplementary Figure 41 illustrates the ^1H NMR spectra of pure **A-1** at different temperatures. When **A-1** is irradiated, it converts to **C-1** *via* metastable state **B-1**. However, at this temperature the metastable state **B-1** is not observable, which indicates a lower barrier of thermal helix inversion for conversion from **B-1** to **C-1** isomer. Only after the signals of **C-1** isomer (see Supplementary Fig. 42 and 43) are present, the signals of **D-1** start rising. Upon warming to −90 °C (Supplementary Fig. 44), the signals of **D-1** convert directly into the isomer **A-1** within 7 min of time and metastable **E-1** isomer cannot be observed due to the low barriers associated with the thermal helix inversion from **E-1** to **A-1**.

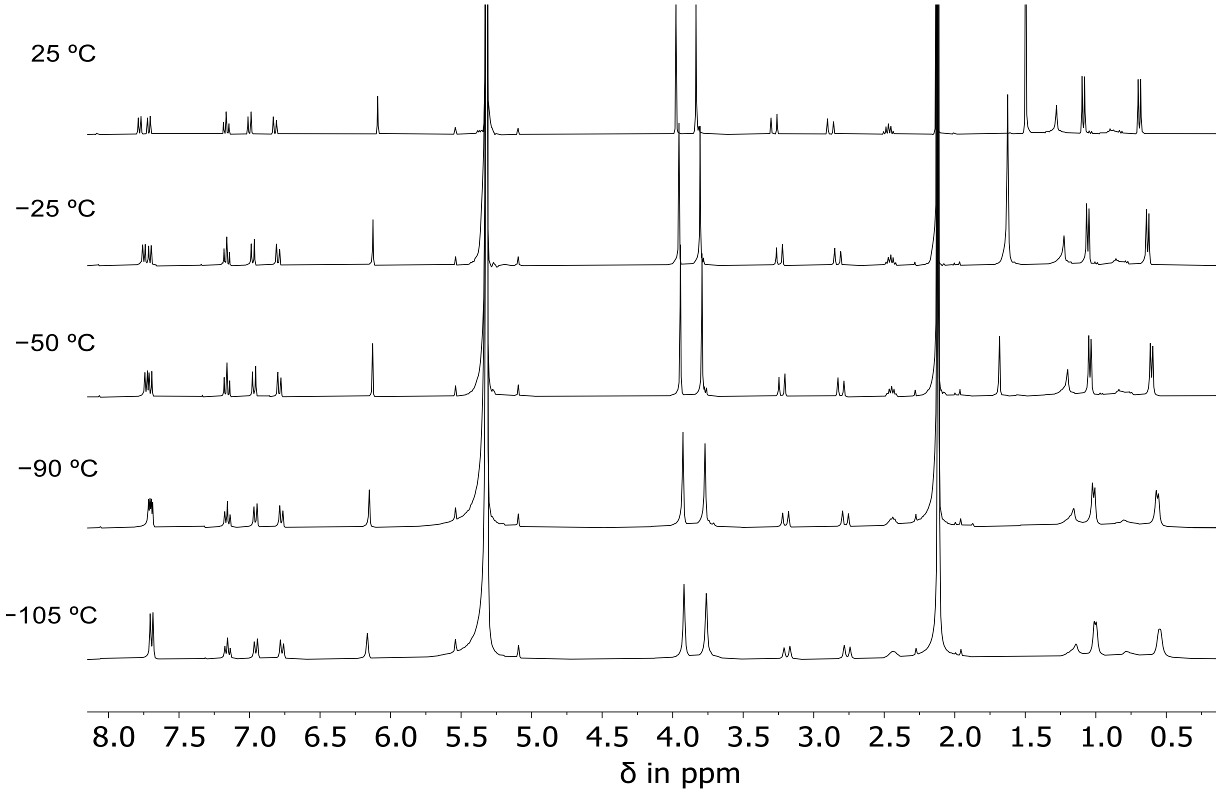

Supplementary Figure 41. Stacked variable-temperature ^1H NMR (CD_2Cl_2:CS_2 = 4:1, 400 MHz) spectra of racemic **A-1** measured at 25 °C, −25 °C, −50 °C, −90 °C and −105 °C.

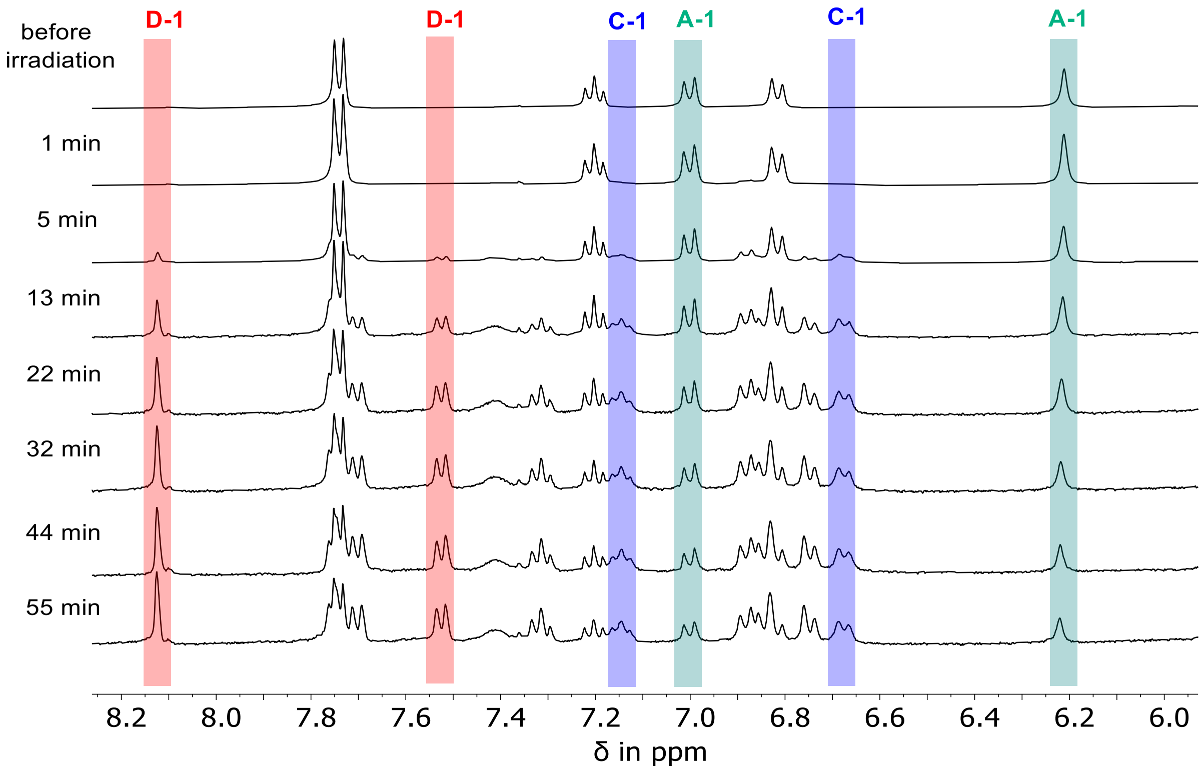

Supplementary Figure 42. ^{1}H NMR spectra (aromatic region) of isomer **A-1** (CD_2Cl_2:CS_2 = 4:1, 400 MHz) measured at −105 °C during irradiation with a high-power 450 nm LED. Indicative signals are assigned exemplary.

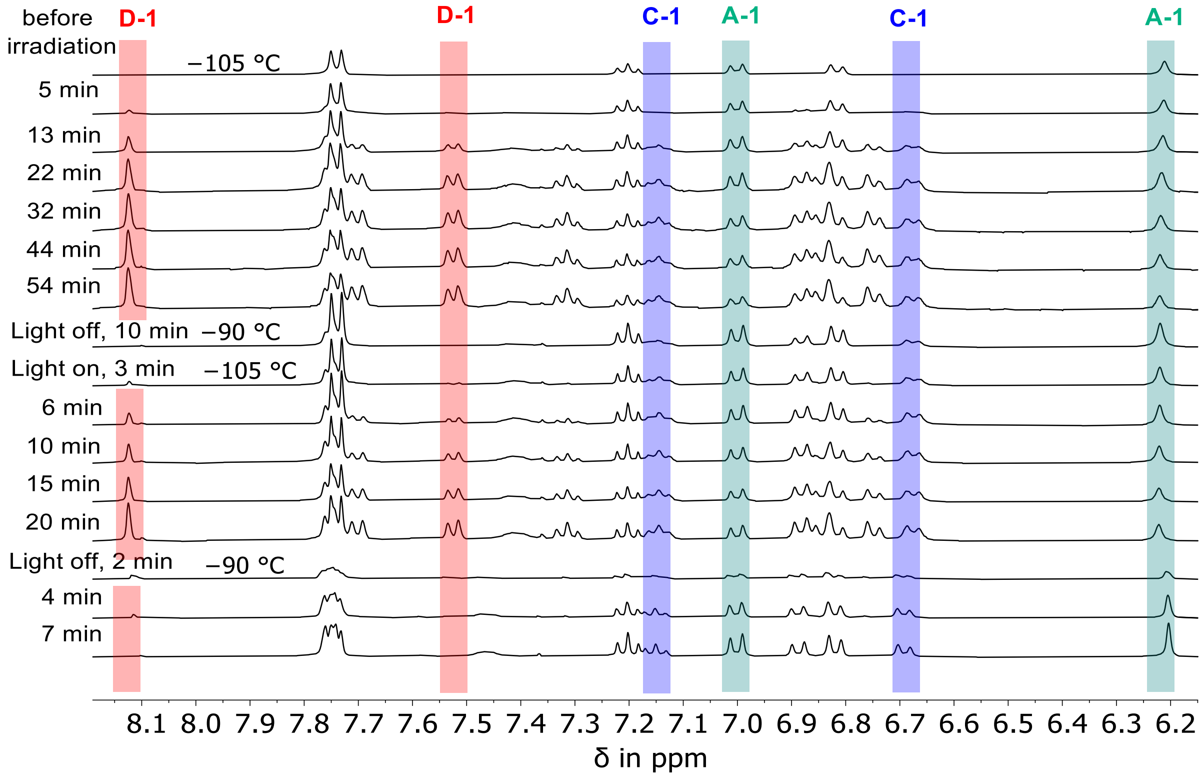

Supplementary Figure 43. ^{1}H NMR spectra (aromatic region) of motor **A-1** (CD_2Cl_2:CS_2 = 4:1, 400 MHz) measured at −105 °C during high power 450 nm irradiation followed by thermal relaxation at −90 °C in the dark, allowing for full conversion of **D-1** to **A-1** isomer. This process was repeated. Signals of different isomers were assigned exemplary.

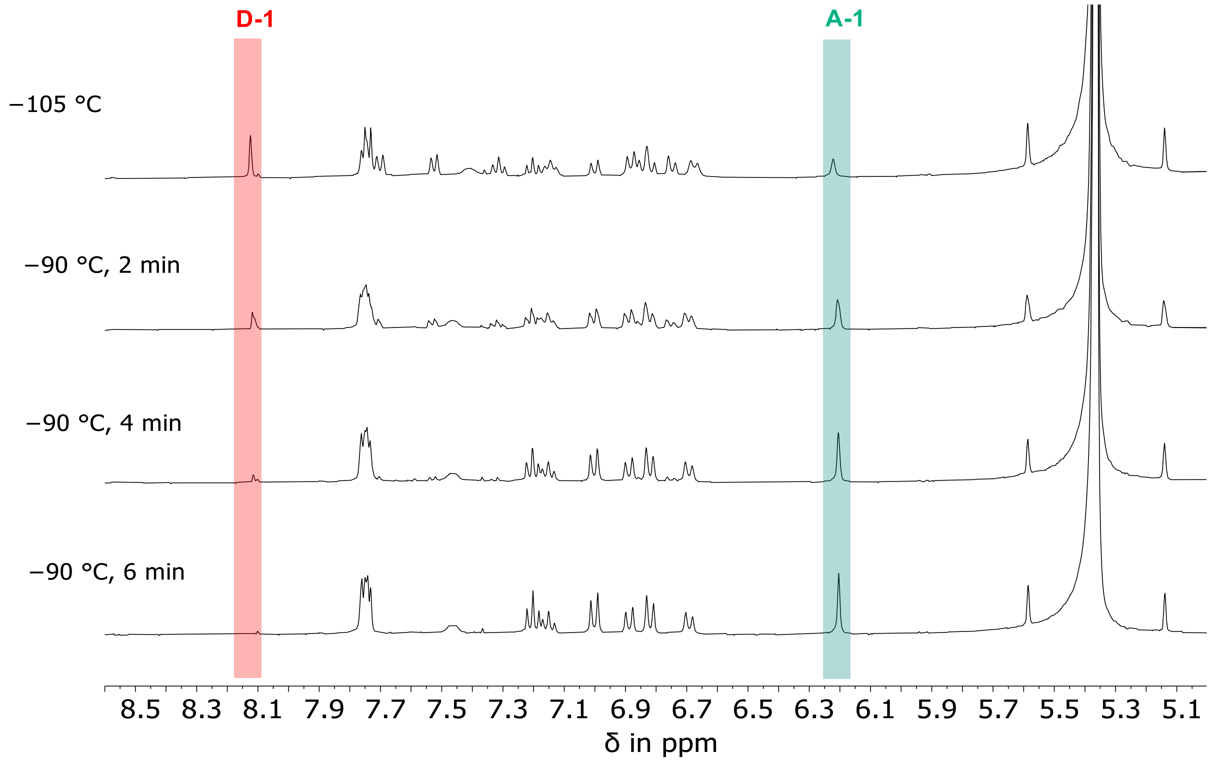

Supplementary Figure 44. ^{1}H NMR spectra (CD_2Cl_2:CS_2 = 4:1, 400 MHz, aromatic region) showing the thermal conversion from **D-1** to **A-1** in the dark at −90 °C.

To further analyze the sequential isomer conversion **A-1** to **B-1** to **C-1** to **D-1**, pure *E*-configured **A** isomer was irradiated with a 450 nm high power LED in THF-d_8 at −105 ºC. First, the ^{1}H NMR spectra of **A-1** and **C-1** were measured in THF-d_8 at different temperatures to enable spectral assignment to these isomers and trace temperature induced signal shifts (Supplementary Fig. 45 and 46). When irradiating **A-1** at –105 °C a clear isomer conversion sequence was observed First isomer **C-1** is formed and only after a substantial amount of it is present, isomer **D-1** is formed next. Upon warming to −80 °C in the dark the **D-1** isomer completely reverted back the initial state **A-1** and no other metastable state was observable.

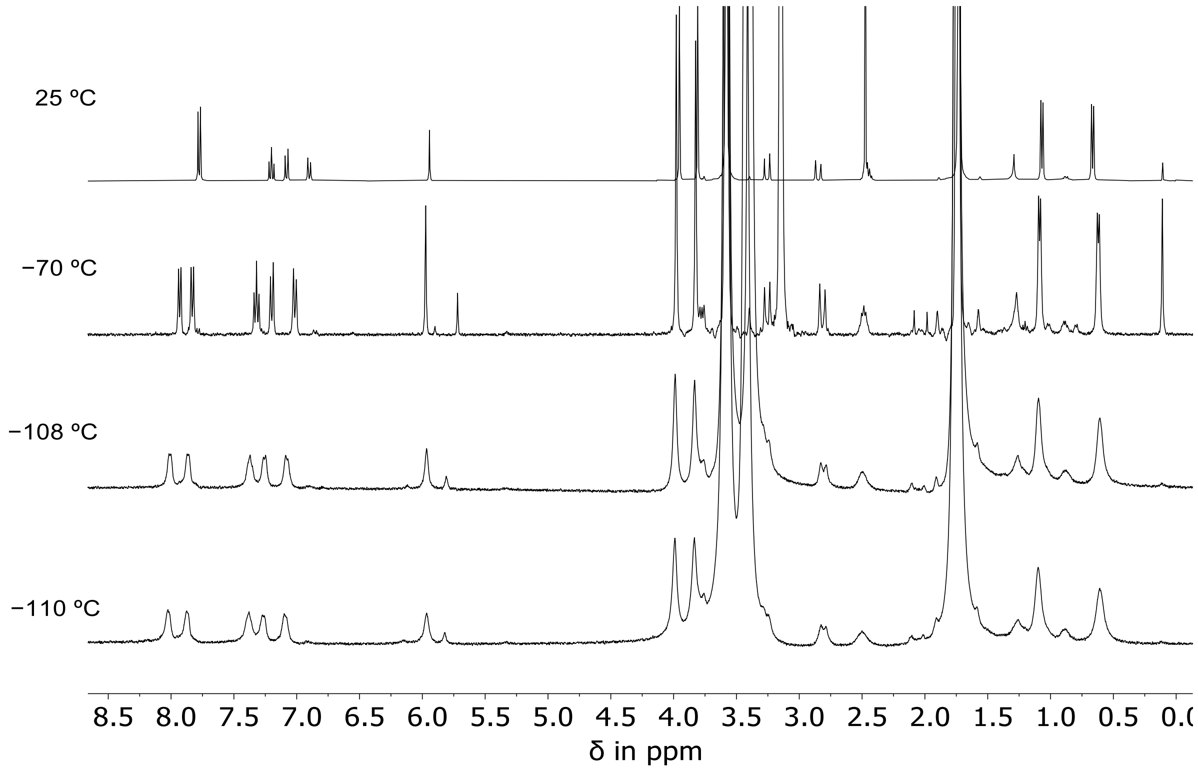

Supplementary Figure 45. Stacked variable-temperature ^{1}H NMR (THF-d_8, 400 MHz) spectra of racemic **A-1** measured at 25 °C, −70 °C, −108 °C and −110 °C.

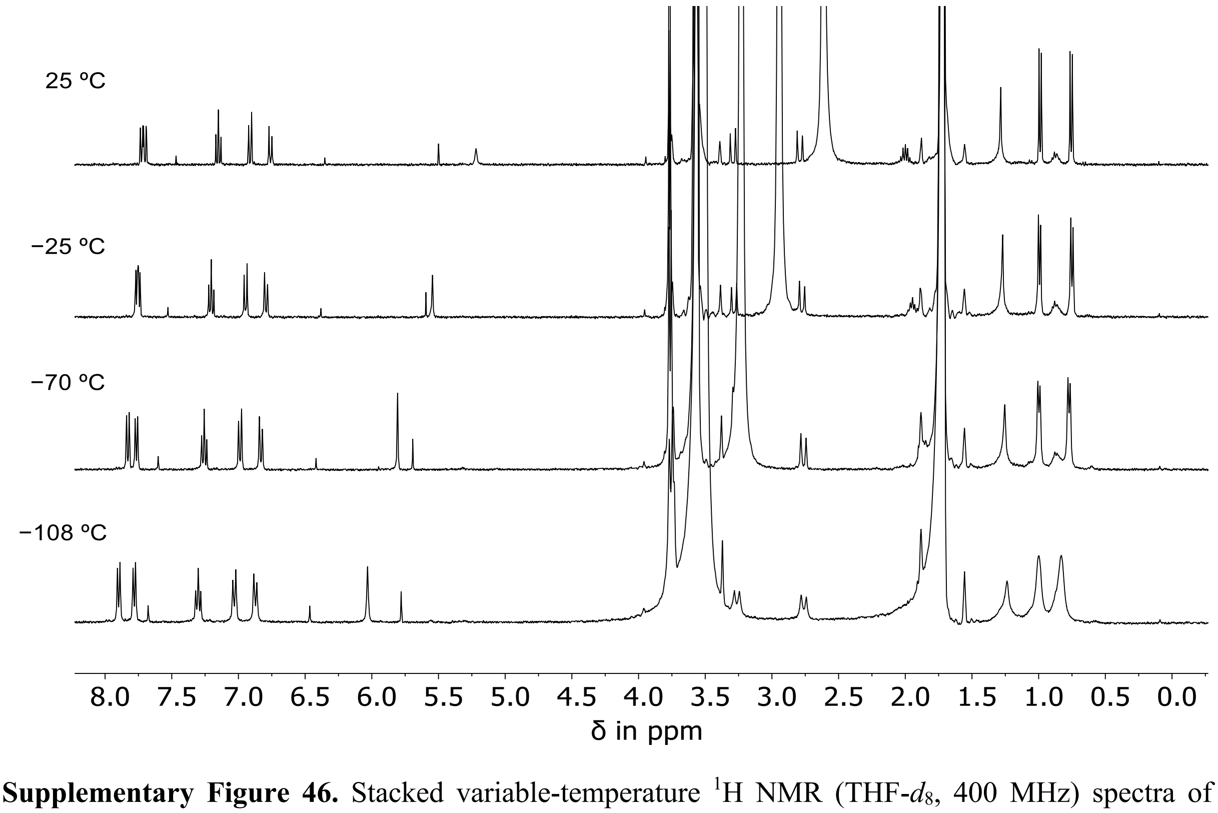

Supplementary Figure 46. Stacked variable-temperature ^{1}H NMR (THF-d_8, 400 MHz) spectra of racemic **C-1** measured at 25 °C, −25 °C, −70 °C, and −108 °C.

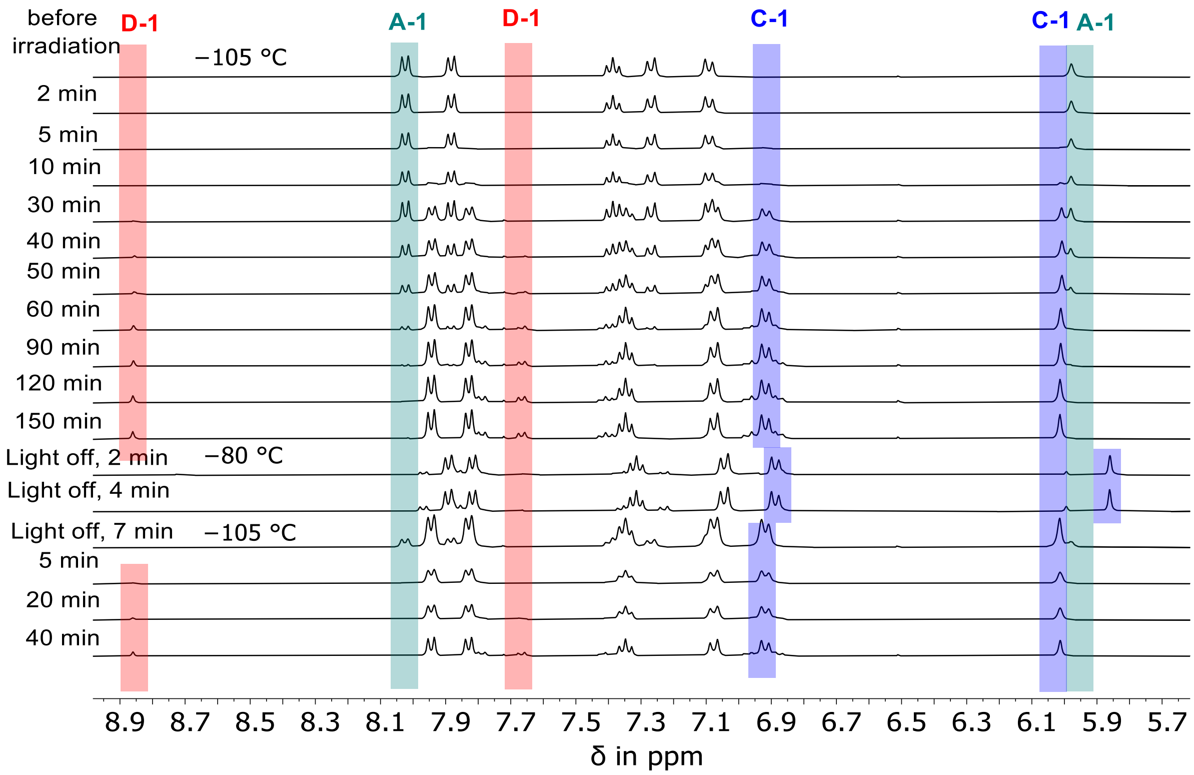

Supplementary Figure 47. ^{1}H NMR spectra (THF-d_8, 400 MHz, aromatic region) of motor **A-1** measured at −105 °C during high power 450 nm irradiation followed by thermal relaxation at −80 °C in the dark. This process was repeated again. Signals of different isomers are assigned exemplarily.

In a separate experiment, the **C-1** to **D-1** to **A-1** conversion sequence of motor **1** was explored by irradiating pure *Z*-configured **C-1** isomer with a 450 nm high power LED in THF-d_8 at −108 ºC (Supplementary Fig. 48). For the more concentrated sample, epoxide **D-1** was quantitatively obtained within 360 min.

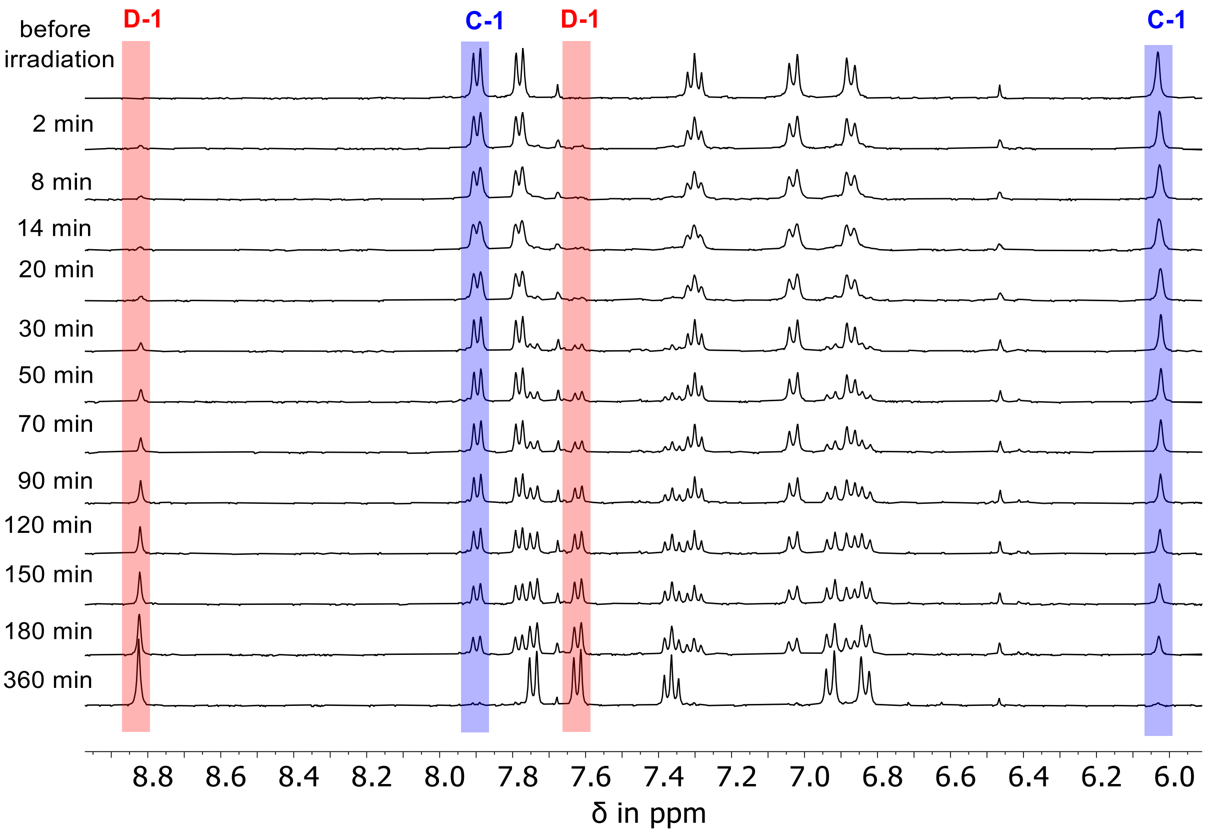

Supplementary Figure 48. ^{1}H NMR spectra (THF-d_8, 400 MHz, aromatic region) of isomer **C-1** measured at −108 °C during high power 450 nm irradiation. Indicative signals are assigned exemplarily. Almost quantitative accumulation of isomer **D-1** is possible under these conditions.

Supplementary Figure 49 shows the thermal conversion from photochemically-generated epoxide **D-1** to stable **A-1**. The related kinetic analysis is also described in the following.

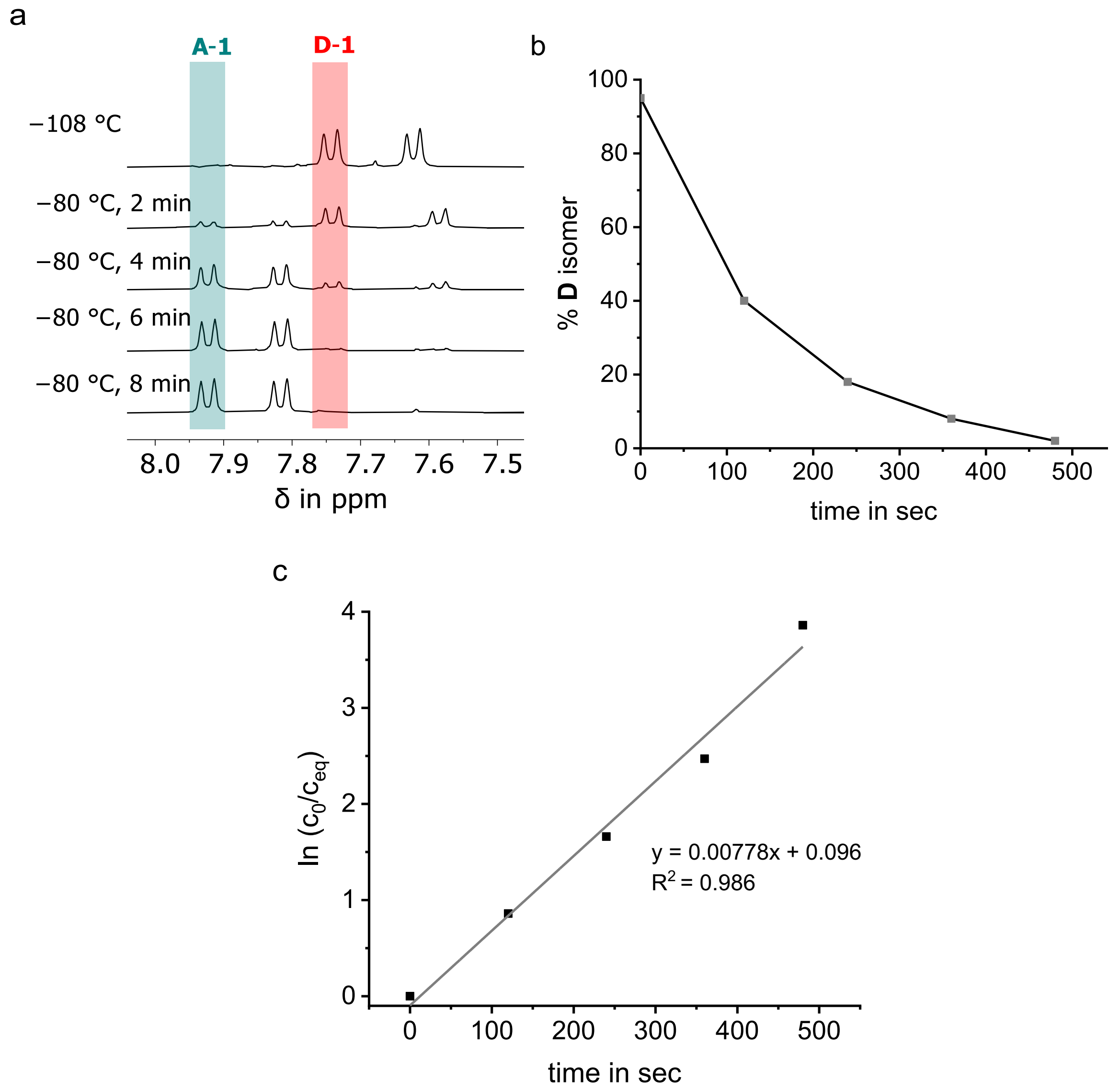

Supplementary Figure 49. Kinetic analysis of the thermal conversion of metastable **D-1** to stable **A-1** in THF-d_8 solution. a) ^{1}H NMR spectra (THF-d_8, 400 MHz) for **D-1** to **A-1** isomerization acquired at −80 °C in the dark. For clarity the gradual conversion of the proton signals in the aromatic region are shown. Spectra were recorded in 2 min time intervals. b) Plot of decrease of **D-1** isomer over time. c) A first-order kinetic analysis of the ^{1}H NMR data, giving a linear relationship. The slope of the linear fit (formula given in the diagram) is the first-order rate constant $k_{(D\rightarrow A)}$ = 0.00778 s^{-1}.

The thermal conversion from metastable **D-1** to stable **A-1** isomer can be analyzed using a first-order kinetics description without entering equilibrium. At –80 °C, the first-order rate constant of thermal **D-1** to **A-1** isomerization is $k_{(D\rightarrow A)}$ = 0.00778 s^{-1}. The Gibbs energy of activation $\Delta G^{\neq}$ for such transformation can be calculated from the rate constant $k_{(D\rightarrow A)}$ of the reaction using the Eyring equation:

$$k_{(D\rightarrow A)} = {}^{(k_B \cdot T)}/_{h} \cdot e^{-\Delta G^{\neq}/_{RT}} \qquad (2)$$

with k_B = Boltzmann constant (1.38 x 10^{-23} J.K^{-1}), T = temperature in K, h = Planck constant (6.626 x 10^{-34} Js), $k_{D\rightarrow A}$ = rate constant of the **D-1** to **A-1** thermal isomerization. After re-arranging equation 2 and including the numerical values of the constants, $\Delta G^{\neq}$ is given by:

$$\Delta G^{\neq}(in\, J \cdot mol^{-1}) = 8.314 \cdot T \cdot [23.760 + \ln\left({T}/{k(D \rightarrow A)}\right) \quad (3)$$

As the rate constant was determined at –80 °C (193.15 K), the activation energy was calculated as $\Delta G^{\neq}$ = 54.4 kJ/mol = 13.0 kcal/mol for the thermal conversion from **D-1** to **A-1** in THF-d_8.

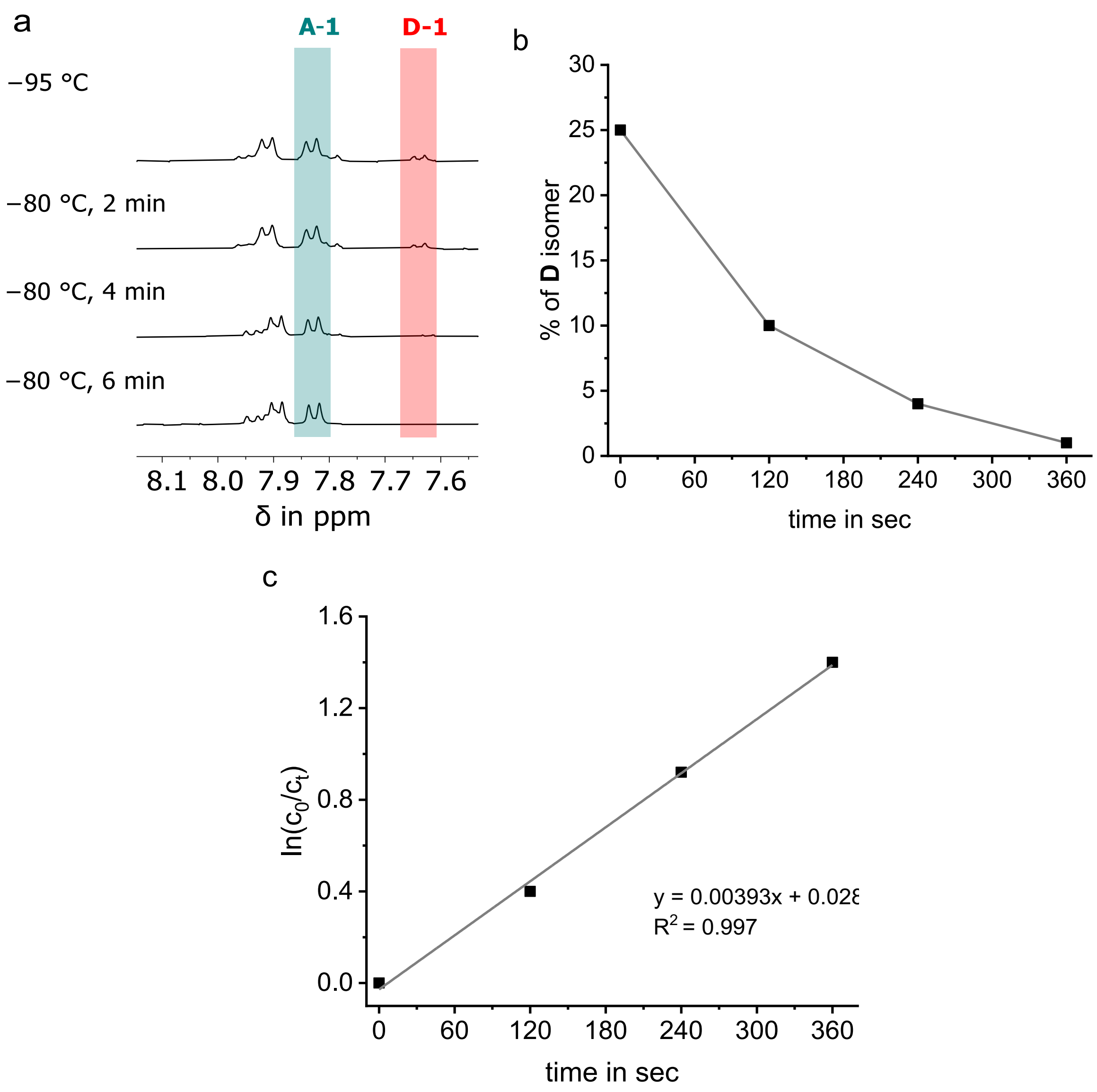

Supplementary Figure 50. Kinetic analysis of the thermal conversion from metastable **D-1** to stable **A-1** isomer in CD_3OD solution. a) ^{1}H NMR spectra (CD_3OD, 400 MHz) for thermal **D-1** to **A-1** isomerization measured at −80 °C in the dark. For clarity the gradual conversion of the proton signals in the aromatic region are shown. Spectra were recorded in 2 min time intervals. b) Plot of decrease of **D-1** isomer over time. c) First-order kinetic analysis of the ^{1}H NMR data, showing a linear relationship. The slope of the linear fit (formula given in the diagram) is the first-order rate constant $k_{(D\rightarrow A)}$ = 0.00393 s^{-1}.

Similarly, the thermal conversion from metastable **D-1** to stable **A-1** isomer in CD_3OD solution was analyzed using a first-order kinetics description (Supplementary Figure 50). At –80 °C, the first-order rate constant of the **D-1** to **A-1** isomerization is $k_{(D\rightarrow A)}$ = 000393 s^{-1}. The Gibbs energy of activation $\Delta G^{\neq}$ for this transformation can be calculated from the rate constant $k_{(D\rightarrow A)}$ of the reaction using the Eyring equation 2, as well as rearranging equation 2 and adding the numerical values of the constants. In this way a corresponding Gibbs energy of activation $\Delta G^{\neq}$ = 55.4kJ/mol = 13.2 kcal/mol was found for the thermal **D-1** to **A-1** isomerization in CD_3OD.

The experiments described above allowed for evidencing the **D-1** intermediate, but the **B-1** intermediate was not visible even in traces within the explored temperature windows. After several attempts to study the irradiation process at lower temperature, presence of the **B-1** intermediate could be evidenced at –130 °C upon irradiation of the **A-1** isomer in a THF-d_8:CS_2 mixture (1:1, v:v). The results are shown in Supplementary Figures 51 and 52.

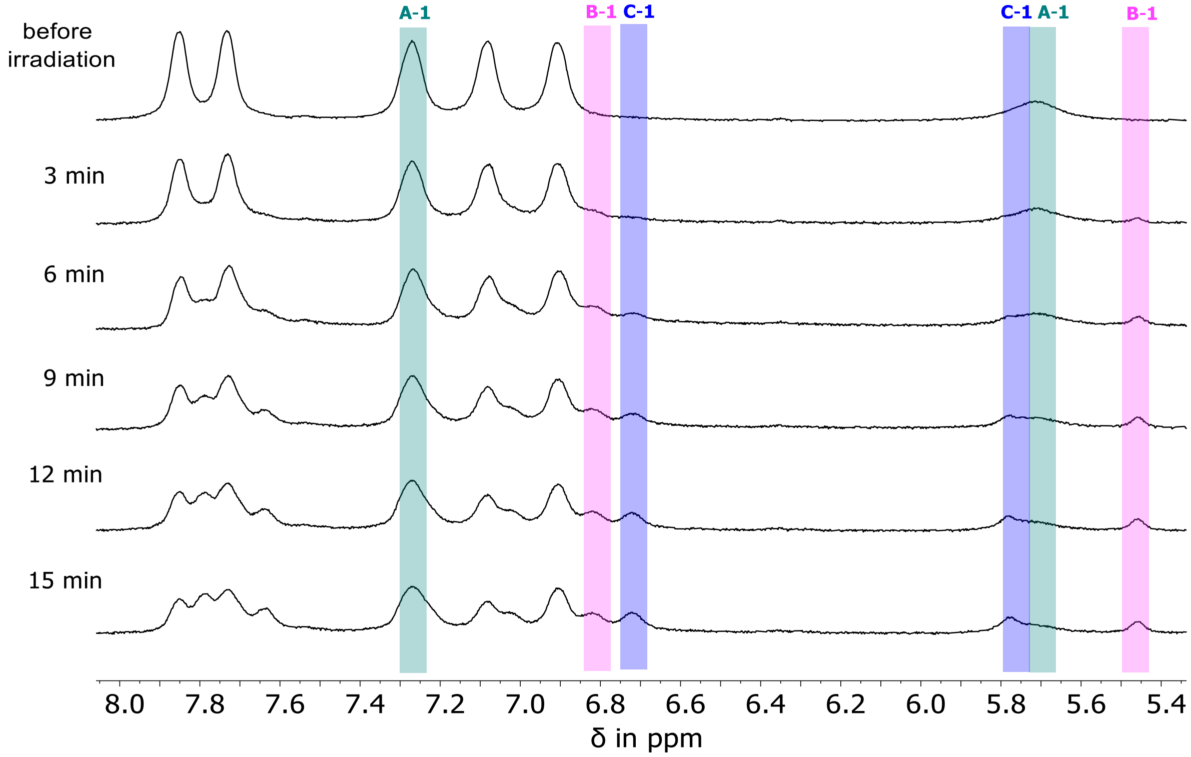

Supplementary Figure 51. ^{1}H NMR spectra (THF-d_8:CS_2 = 1:1, 400 MHz, aromatic region) of isomer **A-1** acquired at –130 °C during high power 450 nm irradiation. Indicative signals are assigned exemplarily. Spectra were recorded in 3 min time intervals.

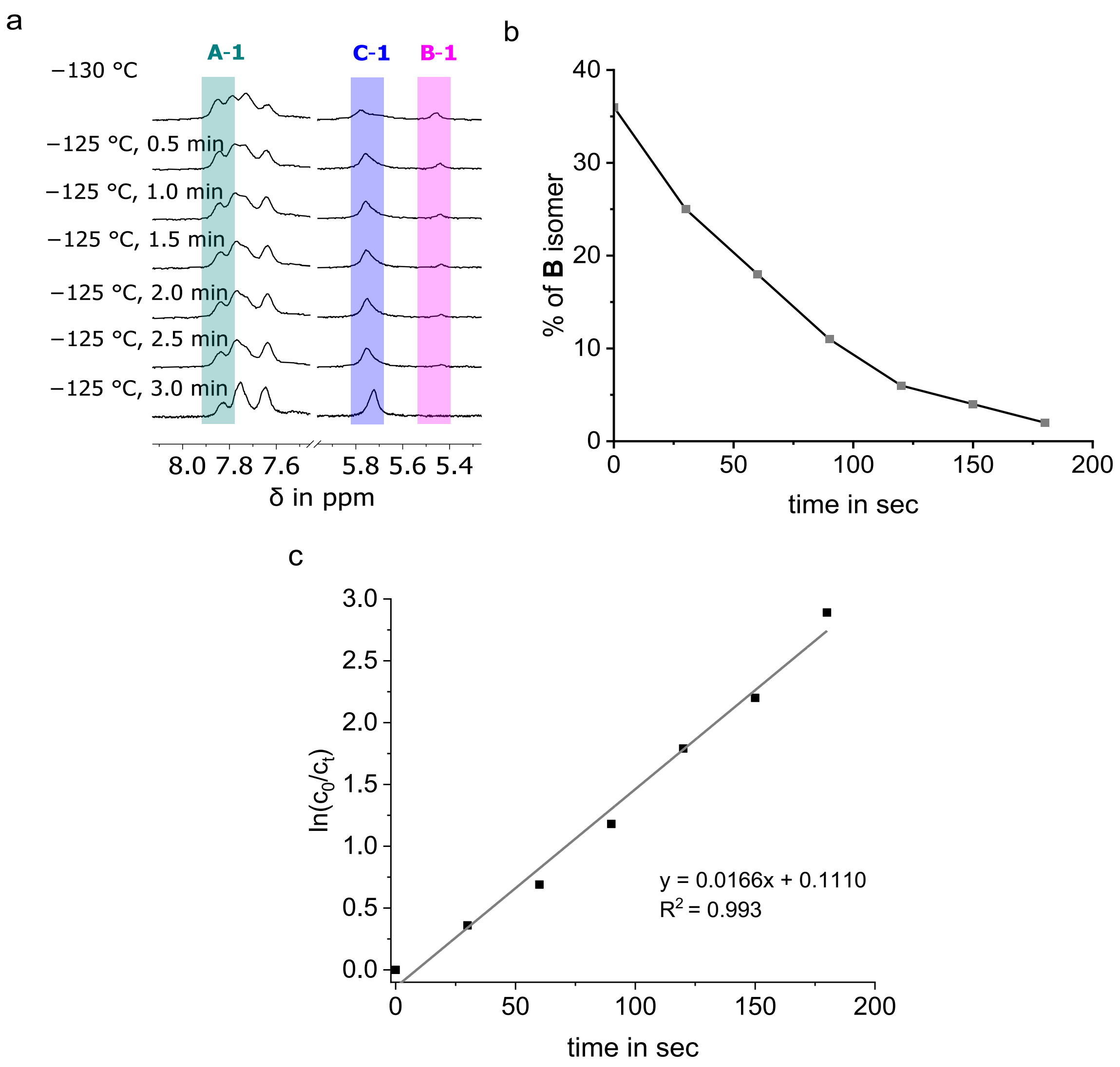

Supplementary Figure 52. Kinetic analysis of the thermal helix inversion from metastable **B-1** to stable **C-1** isomer. a) ^{1}H NMR spectra (THF-d_8:CS_2 = 1:1, 400 MHz) for **B-1** to **C-1** isomerization acquired at −125 °C in the dark. For clarity the gradual conversion of the proton signals in the aromatic region are shown. Spectra were recorded in 0.5 min time intervals. b) Plot of decrease of **B-1** isomer over time. c) First-order kinetic analysis of the ^{1}H NMR data, which shows a linear relationship. The slope of the linear fit (formula given in the diagram) is the first-order rate constant $k_{(B\rightarrow C)}$ = 0.0166 s^{-1}.

As depicted in Supplementary Figure 52, the thermal helix inversion from **B-1** to **C-1** in THF-d_8:CS_2 (1:1, v:v) solution can be analyzed using a first-order kinetics description and, at –125 °C, the first-order rate constant of the thermal **B-1** to **C-1** isomerization is $k_{(B\rightarrow C)}$ = 0.0166 s^{-1}. The Gibbs energy of activation $\Delta G^{\neq}$ for this thermal helix inversion can be calculated from the rate constant $k_{(B\rightarrow C)}$ of the reaction using the rearranged Eyring equation 2 with added numerical values of the constants. From the rate constant at –125 °C (148.15 K) a Gibbs energy of activation of $\Delta G^{\neq}$ = 40.4 kJ/mol = 9.67 kcal/mol was obtained for the thermal helix inversion from **B-1** to **C-1**.

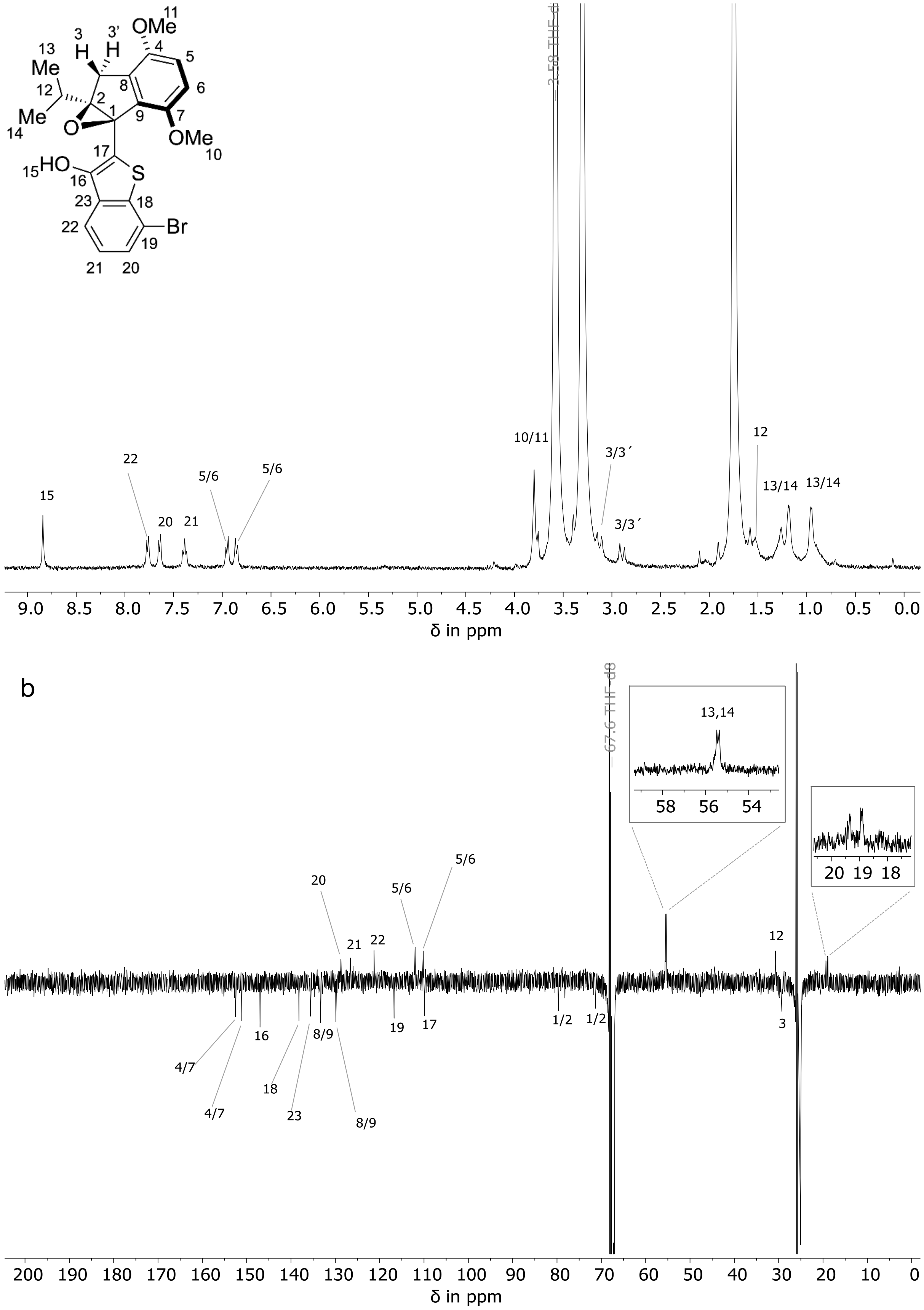

Supplementary Figure 53. 1D NMR spectra of metastable isomer **D** of motor **1**. a) ^{1}H NMR spectrum of pure **D-1** (THF-d_8, 400 MHz, −120 °C). b) ^{13}C DEPTq NMR spectrum of 80% enriched sample of **D-1** ((THF-d_8, 101 MHz, −120 °C). The OCH_3 signal corresponding to H-C10 or H-C11 overlaps with the THF-d_8 solvent peak and appears at 3.60 ppm as deduced from the ^{1}H-^{13}C NMR HMBC experiment.

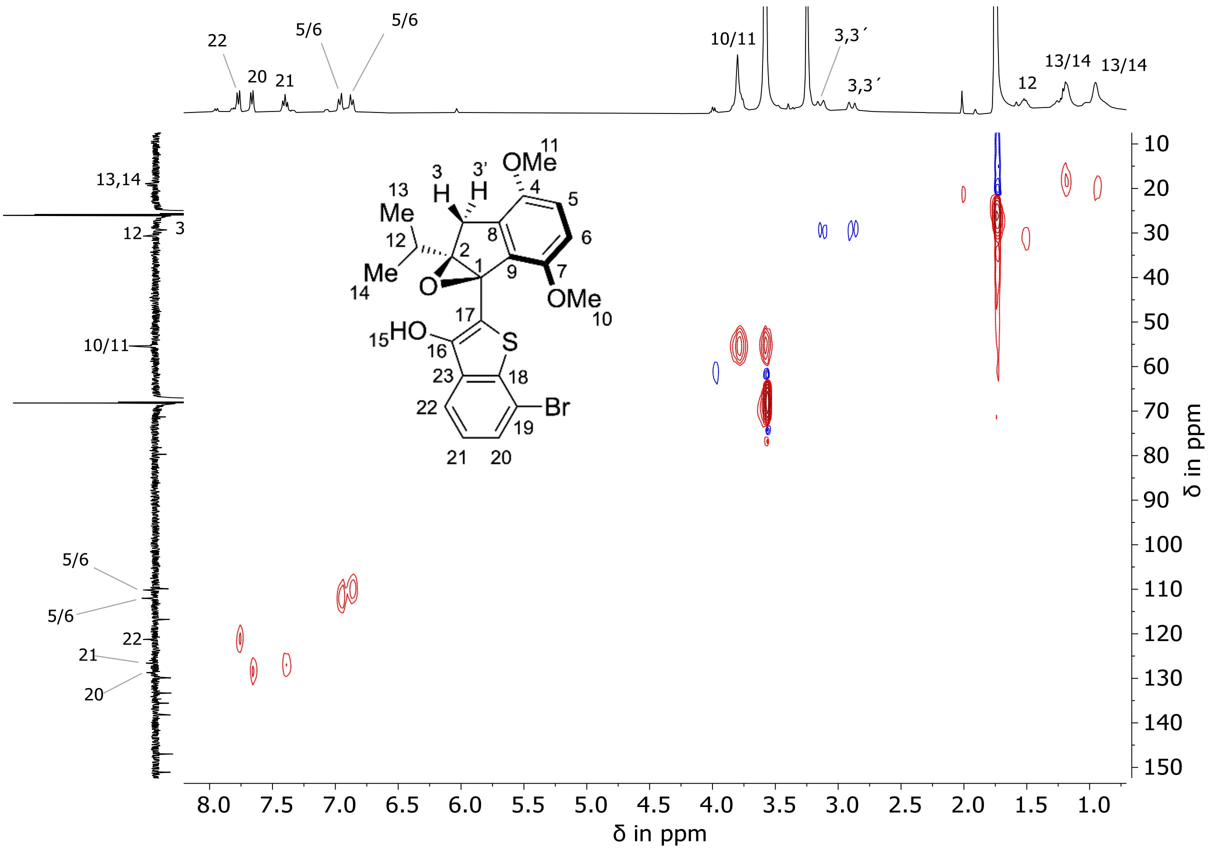

Supplementary Figure 54. ^{1}H-^{13}C NMR HSQC spectrum (THF-d_8, 400 MHz, −125 °C) of metastable isomer **D** of motor **1** and related peak assignments to the molecular structure.

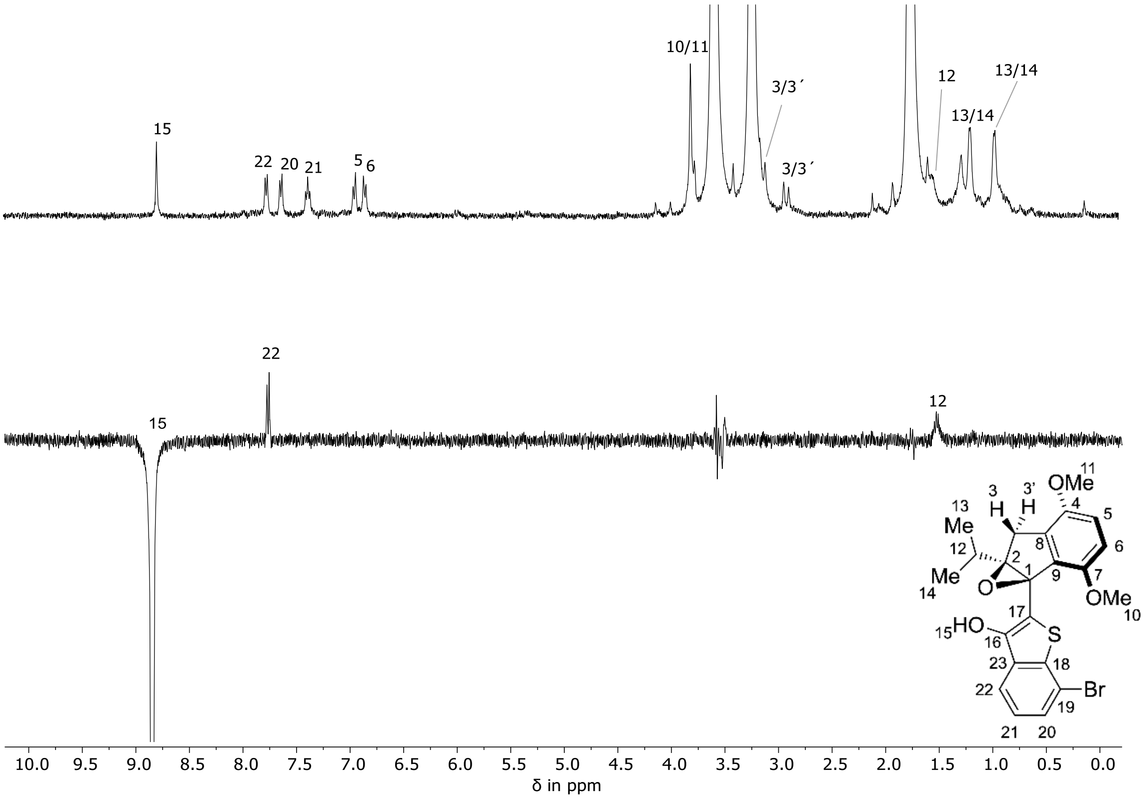

Supplementary Figure 55. 1D NOE experiment (THF-d_8, 400 MHz, −108 °C) of isomer **D** of motor **1** showing couplings of OH proton H-15 with H-C22 and H-C12.

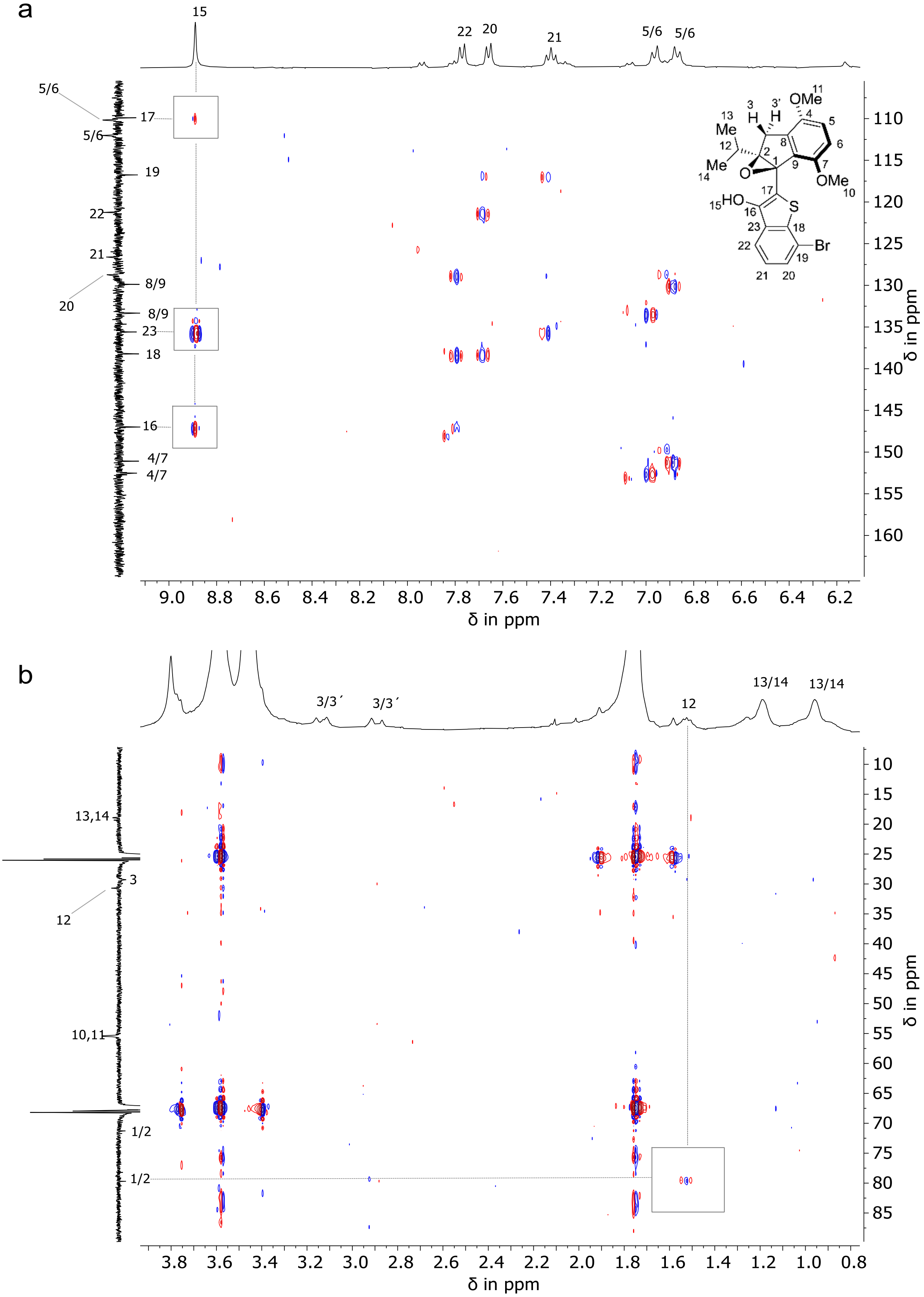

Supplementary Figure 56. ^{1}H-^{13}C NMR HMBC spectrum (THF-d_8, 400 MHz, −120 °C) of 61% enriched metastable **D** isomer of motor **1** and related peak assignments to the molecular structure. a) Enlarged aromatic-aromatic region of the spectrum. b) Enlarged aliphatic-aliphatic and aromatic region and solid boxes.

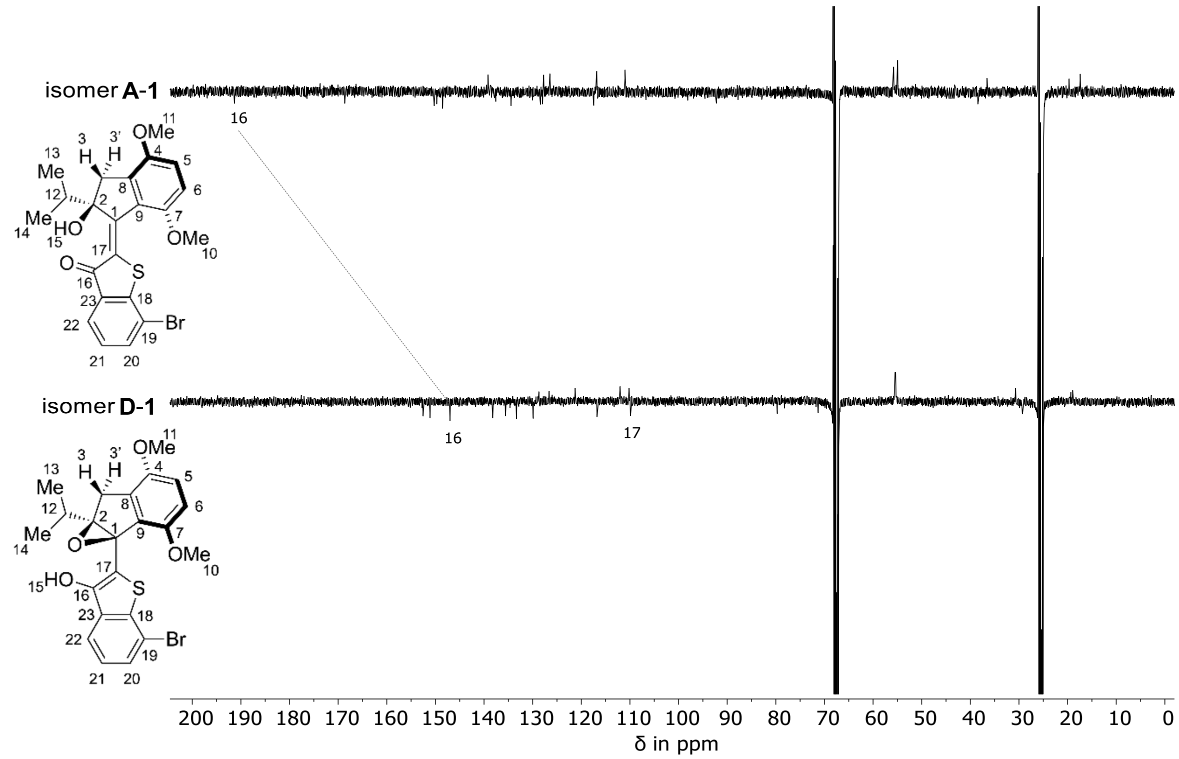

Supplementary Figure 57. Comparison of ^{13}C DEPTq NMR spectra (400 MHz, THF-d_8) of stable **A** and metastable **D** isomers of motor **1** confirming the absence of the C16 carbonyl signal signature in isomer **D**. While the spectrum of **D-1** isomer was recorded at −120 °C, the temperature was set to −108 °C to measure the spectrum of **A-1**.

10. Photophysical Properties of Motor 1

10.1 UV/vis Spectra

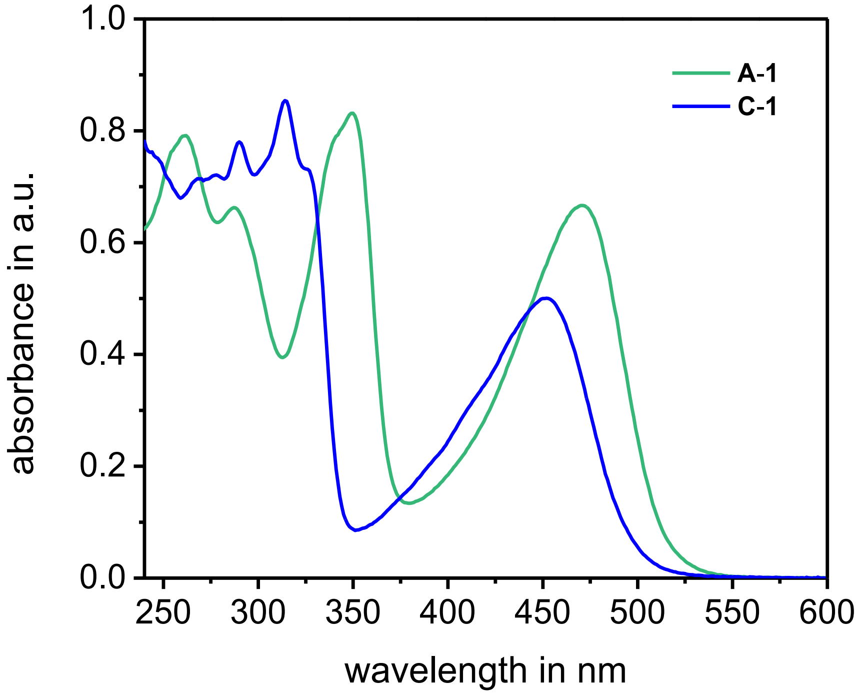

Supplementary Figure 58. UV/vis spectra of *E*-configured isomer **A-1** (green) and *Z*-configured isomer **C-1** (blue) measured in THF at 23 °C. The concentration of isomer **A-1** is 52.6 µM and the concentration of isomer **C-1** is 49.4 µM.

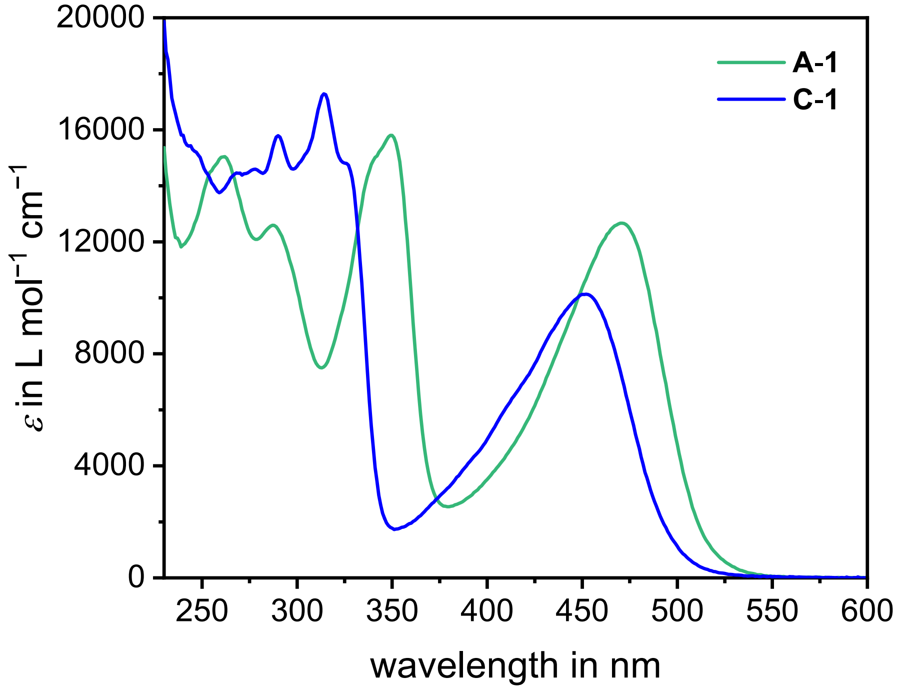

Supplementary Figure 59. Molar extinction coefficients ε of *E*-configured isomer **A-1** (green) and *Z*-configured isomer **C-1** (blue) measured in THF at 23 °C.

10.2 ECD and UV/Vis Measurements

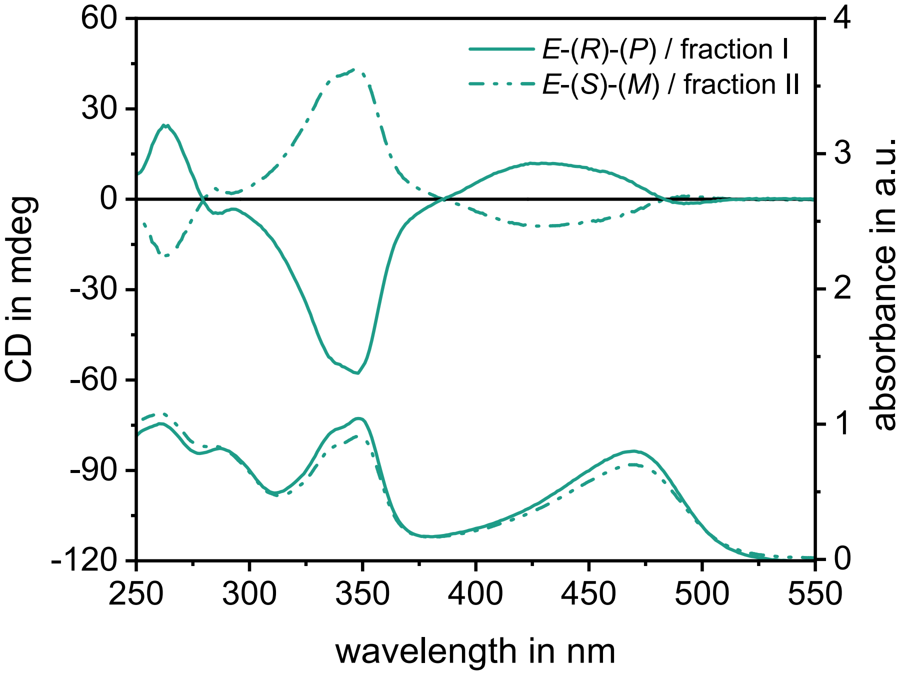

Supplementary Figure 60. ECD and UV/Vis spectra of *E*-(*R*)-(*P*) (green solid line) and *E*-(*S*)-(*M*) (green dashed and dotted line) of motor **1** measured in EPA mixture (Et_2O:*i*-pentane:EtOH, 5:5:2 v/v) at 23 °C. The spectra were measured at different concentrations for each fraction obtained after enantiomer separation of racemic **A-1** through the CSP-HPLC method. According to the theoretical simulations the absolute configuration of fraction I is *E*-(*R*)-(*P*) and of fraction II it is *E*-(*S*)-(*M*).

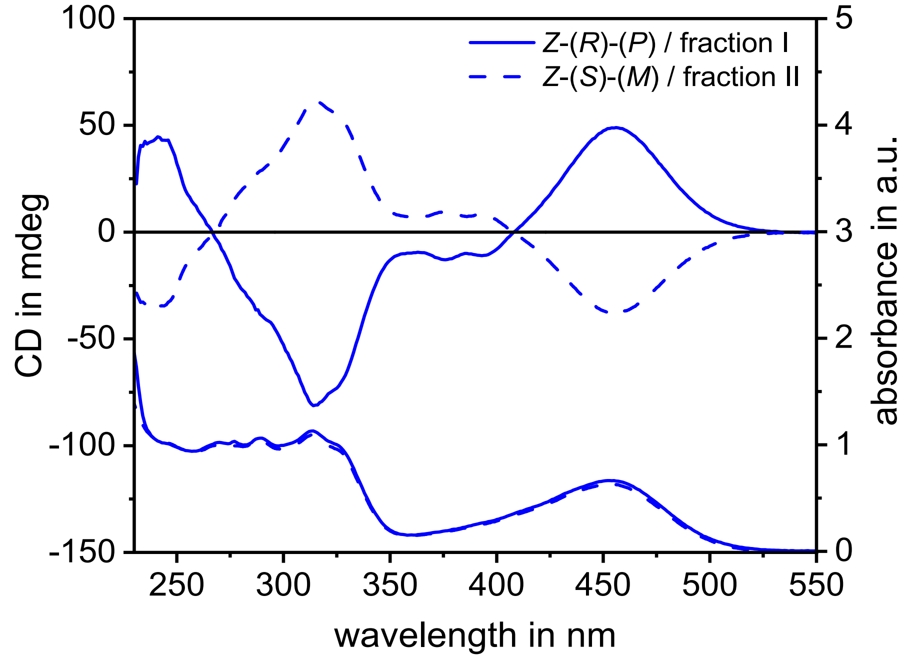

Supplementary Figure 61. ECD and UV/Vis spectra of *Z*-(*R*)-(*P*) (blue solid line) and *Z*-(*S*)-(*P*) (blue dashed and dotted line) of motor **1** measured in EPA mixture (Et_2O:*i*-pentane:EtOH, 5:5:2 v/v) at 23 °C. The spectra were measured at different concentrations for each fraction obtained after enantiomer separation of racemic **C-1** through the CSP-HPLC method. According to the theoretical simulations the absolute configuration of fraction I is *Z*-(*R*)-(*P*) and of fraction II it is *Z*-(*S*)-(*P*).

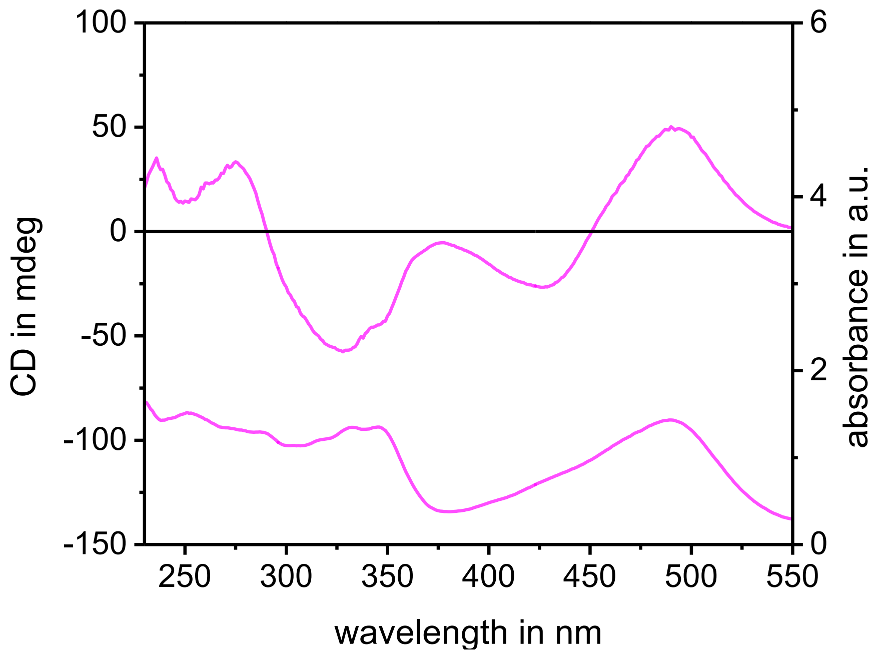

Supplementary Figure 62. ECD and UV/Vis spectra of isomer **B-1** recorded in EPA mixture (Et_2O:*i*-pentane:EtOH, 5:5:2 v/v) at −160 °C. The spectra were obtained after irradiation of the *E*-(*S*)-(*M*) enantiomer of **A-1** with 450 nm light for 71 sec leading to enrichment of **B-1**. Good agreement with the theoretical UV/vis and ECD spectra allowed to assign the absolute configuration of this stereoisomer of metastable **B-1** to Z-(*S*)-(*P*). For clarity and consistency, the recorded ECD spectrum of the (*S*)-configured enantiomer of **B-1** is mirrored in Figure 5 of the manuscript.

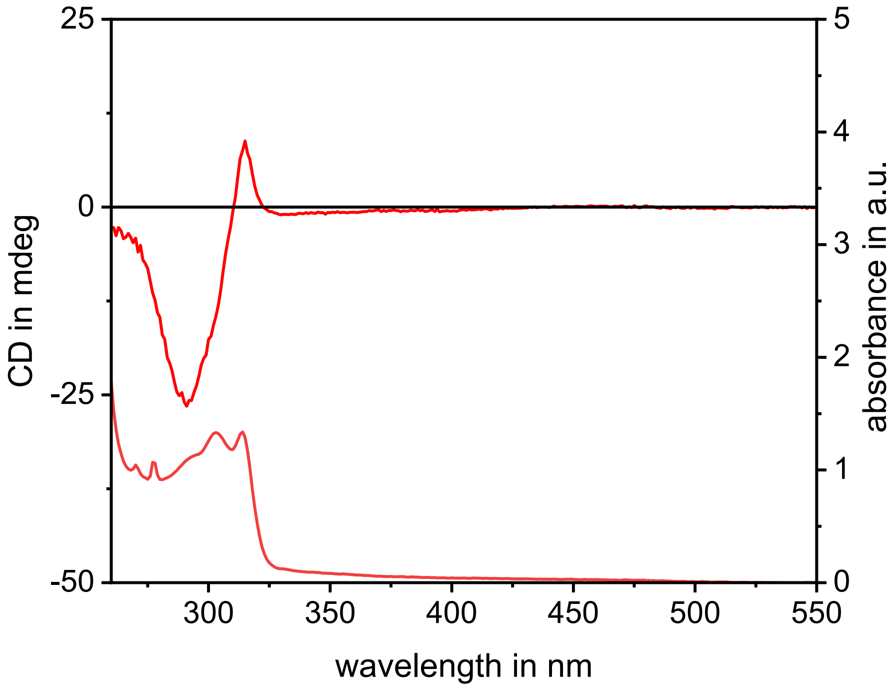

Supplementary Figure 63. ECD and UV/Vis spectra of isomer **D-1** in EPA mixture (Et_2O:*i*-pentane:EtOH, 5:5:2 v/v) at −120 °C. The spectra were acquired after irradiation of *Z*-(*R*)-(*P*) enantiomer of **C-1** with 450 nm light for 69 min. Good agreement with the theoretical UV/vis and ECD simulations allowed to assign the absolute configuration of this stereoisomer of metastable **D-1** to (*R*)-(*R*)-epoxide.

11. Low-Temperature ECD Irradiations and Thermal Annealing

In order to evidence the metastable **B-1** isomer ECD and UV/Vis irradiation experiments were performed in EPA (mixture of Et_2O:*i*-pentane:EtOH, 5:5:2 v/v) at −160 °C. Starting from pure *E*-(*S*)-(*M*) enantiomer of **A-1**, the sample was irradiated externally with 450 nm high-power LED in second-regime time intervals to precisely follow the **A-1** to **B-1** photoreaction. Supplementary Figure 64 shows that metastable isomer **B-1** can be enriched strongly within 71 sec as judged by the inversion of the Cotton effects. Its absolute configuration corresponds to the *Z*-(*S*)-(*P*) stereoisomer according to comparison with theoretical ECD calculations. Also, further 30 sec of irradiation does not lead to any changes in spectral fingerprints, meaning that within 71 sec it was possible to reach the PSS associated with **A-1** to **B-1** isomerization.

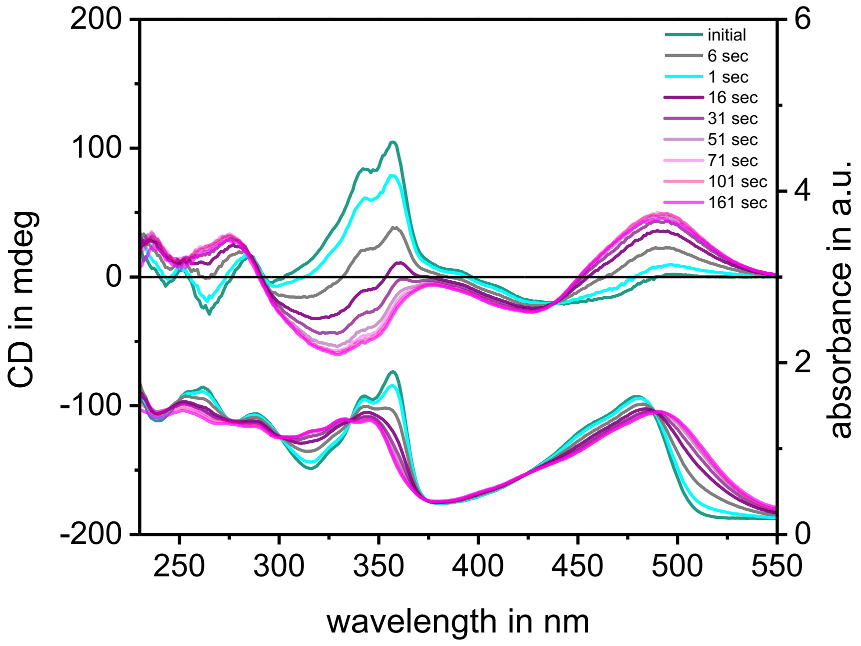

Supplementary Figure 64. Overview of ECD and UV/Vis spectra recorded during irradiation of the *E*-(*S*)-(*M*) stereoisomer of **A-1** with 450 nm light at −160 ° C in EPA (mixture of Et_2O:*i*-pentane:EtOH, 5:5:2 v/v). Stacked spectra were measured in defined time intervals during irradiation. For clarity and consistency, the recorded ECD spectra of the (*S*)-configured **A-1** and **B-1** isomers are depicted as mirrored in Figure 5 of the manuscript.

Next, thermal annealing experiments with **B-1** were carried out by slowly warming up from −160 °C to −80 °C in the dark. The corresponding spectra are presented in Supplementary Figure 65. With these experiments the half-life of **B-1** could be approximated to 36 min at −118

°C (note that this value is derived from experiments with changing temperature), which corresponds to a Gibbs energy of activation of 11.2 kcal/mol. This value is in good agreement with the value of 9.7 kcal/mol as determined by NMR spectroscopy (see above Supplementary Fig. 52). At −108 °C the metastable state **B-1** completely vanishes and quantitatively converts to the *Z*-(*S*)-(*M*) stereoisomer of **C-1**. Overall, these results further evidence and quantify the **A-1** to **B-1** to **C-1** isomer interconversion sequence for the motor cycle completing a 180° unidirectional rotation.

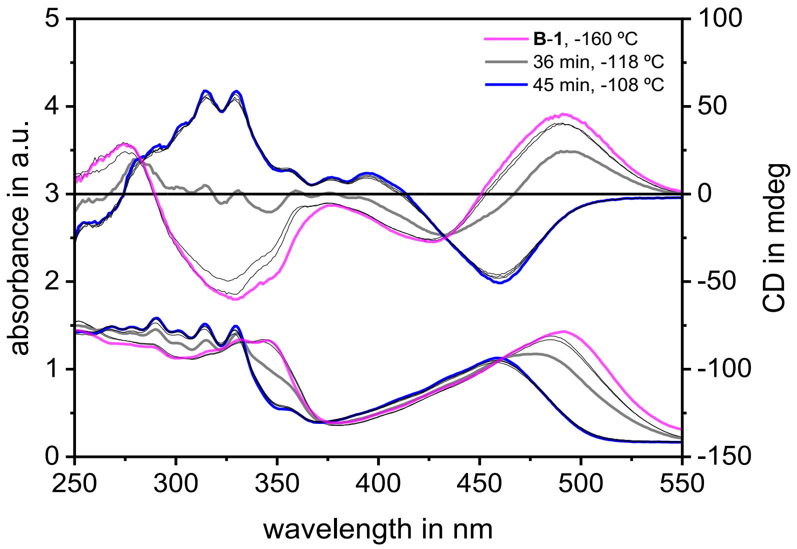

Supplementary Figure 65. Overview of ECD and UV/Vis spectra recorded during thermal annealing experiments of **B-1**. Stacked spectra were measured in defined time intervals at different temperatures in the dark, starting from the strongly enriched *Z*-(*S*)-(*P*) stereoisomer of **B-1** in EPA (mixture of Et_2O:*i*-pentane:EtOH, 5:5:2 v/v) at −160 °C. Only the initial spectrum (pink, *Z*-(*S*)-(*P*) stereoisomer of **B-1**) and the spectra recorded after 36min (grey, corresponding approximately to thermal half-life) and 45 min (blue, *Z*-(*S*)-(*M*) stereoisomer of **C-1**) are highlighted for clarity. Grey lines depict the spectra recorded in the interim. After 36 min at –118 °C about half of the **B-1** population is converted as judged from the ECD spectrum. For clarity and consistency, the recorded ECD spectra of the (*S*)-configured **B-1** and **C-1** isomers are mirrored in Figure 5 of the manuscript.

To prove the existence of epoxide intermediate **D-1** within the rotational cycle of motor **1**, the enantiomerically pure *Z*-(*S*)-(*P*) stereoisomer of **C-1** was irradiated externally with a 450 nm LED in EPA (mixture of Et_2O:*i*-pentane:EtOH, 5:5:2 v/v) at −120 °C (Supplementary Figure 66). According to the acquired ECD and UV/Vis spectra, isomer **D-1** can be enriched quantitatively after 69 min of irradiation. No significant changes were observed when the sample was irradiated for additional 10 min, meaning that the PSS was reached within 69 min. In agreement with the theoretical calculations, the absorption bands within the range of 320 nm and 550 nm are completely vanishing upon transformation to the metastable epoxide intermediate **D-1**. The resulting two carbon stereocenters both adopt (*R*)-configuration.

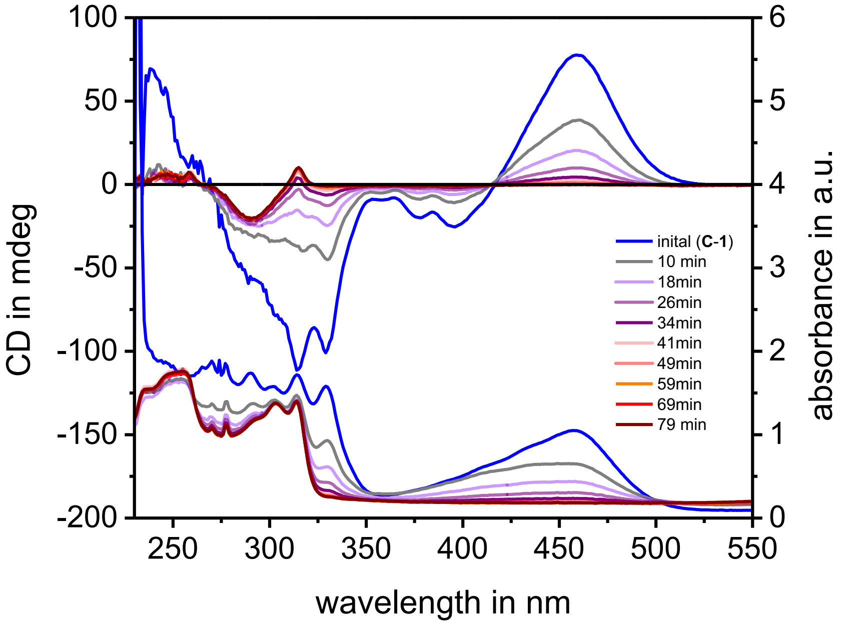

Supplementary Figure 66. Overview of ECD and UV/Vis spectra obtained during irradiation of the *Z*-(*R*)-(*P*) stereoisomer of **C-1** with 450 nm light at −120 °C in EPA (mixture of Et_2O:*i*-pentane:EtOH, 5:5:2 v/v). Stacked spectra were measured in defined time intervals during irradiation. As there are no further changes in both the ECD and UV/Vis spectra, the PSS for **C-1** to **D-1** isomerization is reached within 69 min.

Thermal annealing experiments were then conducted by warming the EPA solution of **D-1** from −120 °C to −80 °C in the dark. The corresponding spectra are depicted in Supplementary Figure 67. Stable **A-1** starts appearing at −100 °C after a total of 15 min and metastable epoxide **D-1** completely vanishes at −80 °C and quantitatively converts to the *E*-(*R*)-(*P*) stereoisomer of **A-1**. Overall, these results further evidence the **C-1** to **D-1** to **A-1** isomer interconversion sequence for the motor cycle completing the second 180° unidirectional rotation part. Metastable **E-1** could not be observed experimentally due to the low barrier associated with thermal helix inversion from **E-1** to **A-1**.

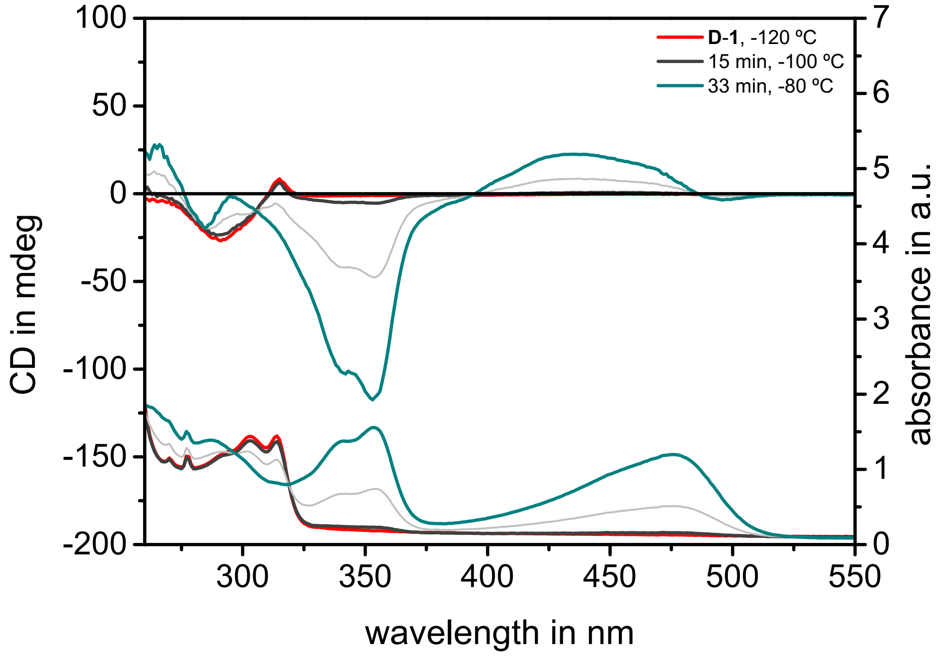

Supplementary Figure 67. Overview of ECD and UV/Vis spectra obtained during thermal annealing experiments of **D-1**. Stacked spectra were measured in defined time intervals at different temperatures in the dark, starting from enriched (*R*)-(*R*) stereoisomer of **D-1** in EPA (mixture of Et_2O:*i*-pentane:EtOH, 5:5:2 v/v) at −120 °C. Only initial (red, (*R*)-(*R*) stereoisomer of **D-1**), after 15 min (dark grey) and 33 min (cyan, *E*-(*R*)-(*P*) stereoisomer of **A-1**) spectra are highlighted for reasons of clarity. Grey lines depict the spectra recorded in the interim.

12. Experimental Data on Sunlight-Driven Molecular Solar Thermal Energy Storage by Supercharged D-1

A-1 was dissolved in 2 mL EPA (mixture of Et_2O:*i*-pentane:EtOH, 5:5:2, v:v) and irradiated with 450 nm light for 3 min to populate the **C-1** state. An absorption spectrum was recorded to confirm enrichment of **C-1** (Supplementary Figure 68). Next, 1 mL of the solution was transferred to a second vial for sunlight irradiation while the remaining solution was used as control. A photograph was taken of both vials, before they were cooled using a liquid N_2/*n*-pentane cooling bath to keep the temperature at around -110 °C. At low temperatures, the sample vial was exposed to sunlight for 15 min while the control reference was kept in the dark. After sunlight irradiation, both vials were photographed to evidence the formation of intermediate **D-1** by visible discoloration of the irradiated sample (Supplementary Figure 69). Upon allowing the sample to warm up to 22 °C, a quick color change back to yellow was observed and the absorption spectrum confirmed virtually quantitative enrichment of the **A-1** isomer (Supplementary Figure 70). In conclusion, sunlight exposure was found to be capable of promoting **C-1** to **D-1** isomer interconversion at low temperatures, while a substantial amount of input energy is stored in the metastable epoxide intermediate **D-1**. This storing state can be identified straight forwardly by the naked eye via decoloration of the solution. The stored energy can then be released by allowing **D-1** to warm up and convert into the stable isomer **A-1**.

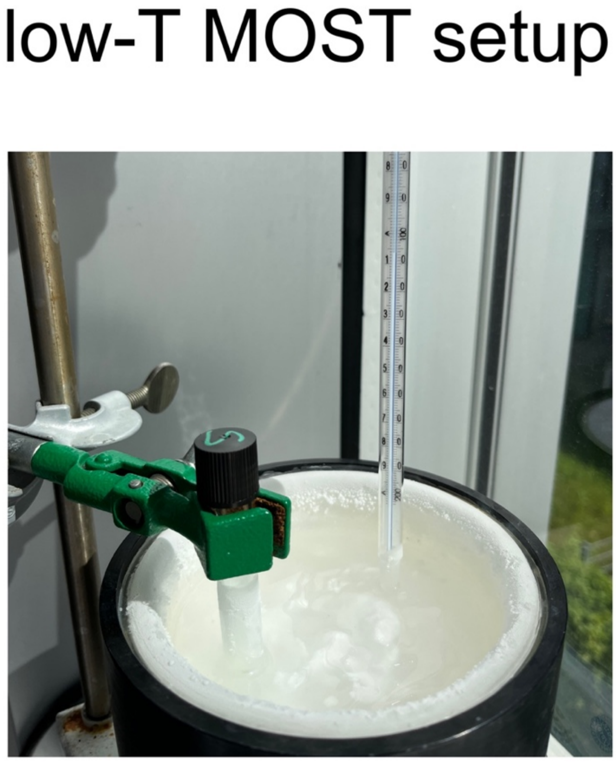

Supplementary Figure 68. Experimental setup for harnessing the energy of sunlight in isomer **D-1** at low temperature. An EPA (mixture of Et_2O:*i*-pentane:EtOH, 5:5:2 v/v) solution containing **C-1** was cooled to –120 °C within a transparent bath of *i*-pentane. The low temperature was maintained by repeated addition of liquid nitrogen during the course of the experiment. Sunlight was allowed to reach into the cooled solution by placing it at the laboratory window. After 15-20 min sunlight irradiation the solution was completely decolored and thus highly enriched in **D-1**.

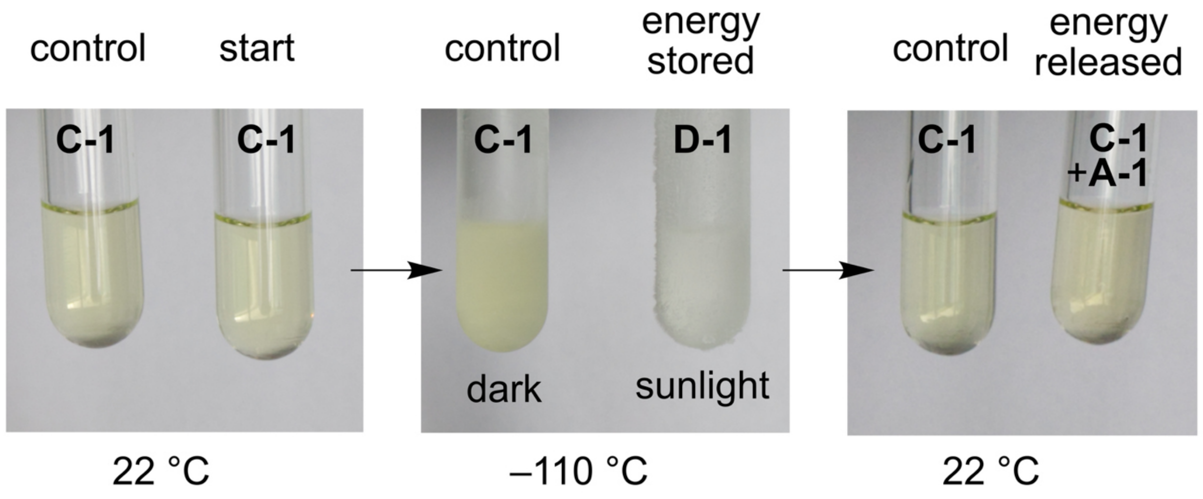

Supplementary Figure 69. Photographs of the EPA (mixture of Et_2O:*i*-pentane:EtOH, 5:5:2 v/v) solution containing **C-1** before (left image) and after sunlight irradiation at –120 °C for 15-20 min (middle image). After sunlight irradiation the solution was completely decolored and thus highly enriched in **D-1**. After subsequent warming up to 22 °C, the same solution turned yellow again, indicative of thermal energy release and conversion of **D-1** to **A-1** (right image). Another sample of the same initial **C-1** solution was kept in the dark during the experiment as a control showing the retained yellow color of the **C-1** isomer in the absence of solar irradiation (left sample vials). Note that for direct

comparison purpose, the control sample was also cooled to –120 °C when comparing it with the irradiated sample (middle image).

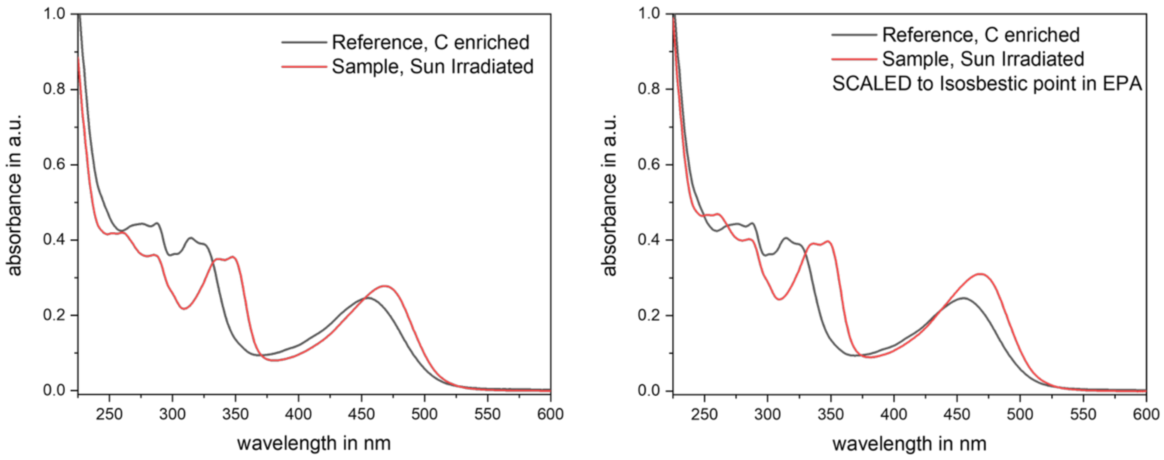

Supplementary Figure 70. UV/vis spectra of an EPA (mixture of Et_2O:*i*-pentane:EtOH, 5:5:2 v/v) solution containing **C-1** recorded at 22 °C before (black) and after (red) low temperature sunlight irradiation and subsequent warming to 22 °C. The spectra on the left were not further processed, while the spectra on the right were adjusted to the same isosbestic point. Full conversion from **C-1** to **A-1** is observed in this experiment.

13. Crystal Structure Data

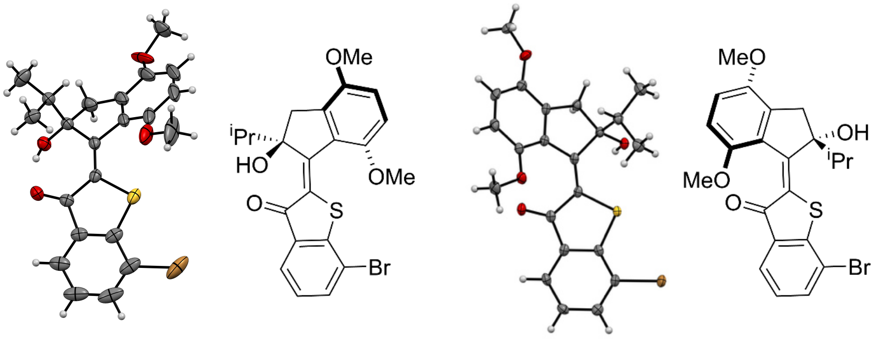

Supplementary Table 8. Crystal Data for **A-1** and **C-1** isomer

Compound	A-1 (CCDC 2307157)	C-1 (CCDC 23071578)
**Formula**	$C_{22}H_{21}BrO_4S$	$C_{23.75}H_{25}BrO_4S$
$D_{calc.}$ / g·cm^{-3}	1.520	1.500
μ / mm^{-1}	3.964	3.739
**Formula Weight**	461.36	486.41
**Color**	clear light red	clear light orange
**Shape**	block-shaped	needle-shaped
**Size/ mm^3**	0.26×0.09×0.08	0.09×0.02×0.01
T/ K	153(1)	100.00(10)
**Crystal System**	triclinic	triclinic
**Space Group**	P-1	P-1
a / Å	6.5649(4)	9.7933(2)
b / Å	9.4961(4)	10.9120(2)
c / Å	16.7764(7)	20.7705(5)
α/°	86.705(4)	99.152(2)
β/°	88.092(4)	99.978(2)
γ/°	74.891(5)	91.695(2)
**V / Å^3**	1007.85(9)	2154.50(8)
Z	2	4
Z'	1	2
**Wavelength/Å**	1.54184	1.54184
**Radiation type**	Cu K	Cu K
Θ_{min} / °	2.639	2.190
Θ_{max} / °	71.571	67.066
**Measured Refl's.**	16048	32586
**Indep't Refl's**	3848	7683
**Refl's I≥2 (I)**	3269	6792
R_{int}	0.0363	0.0370
**Parameters**	272	580
**Restraints**	2	105
**Largest Peak**	0.680	0.428
**Deepest Hole**	-1.206	-0.346
**GooF**	1.039	1.007
wR_2 **(all data)**	0.1109	0.0632
wR_2	0.1041	0.0610
R_1 **(all data)**	0.0519	0.0323
R_1	0.0434	0.0265

14. References

1. Gaussian 16, Revision C.01, Frisch, M. J.; Trucks, G. W.; Schlegel, H. B.; Scuseria, G. E.; Robb, M. A.; Cheeseman, J. R.; Scalmani, G.; Barone, V.; Petersson, G. A.; Nakatsuji, H.; Li, X.; Caricato, M.; Marenich, A. V.; Bloino, J.; Janesko, B. G.; Gomperts, R.; Mennucci, B.; Hratchian, H. P.; Ortiz, J. V.; Izmaylov, A. F.; Sonnenberg, J. L.; Williams-Young, D.; Ding, F.; Lipparini, F.; Egidi, F.; Goings, J.; Peng, B.; Petrone, A.; Henderson, T.; Ranasinghe, D.; Zakrzewski, V. G.; Gao, J.; Rega, N.; Zheng, G.; Liang, W.; Hada, M.; Ehara, M.; Toyota, K.; Fukuda, R.; Hasegawa, J.; Ishida, M.; Nakajima, T.; Honda, Y.; Kitao, O.; Nakai, H.; Vreven, T.; Throssell, K.; Montgomery, J. A., Jr.; Peralta, J. E.; Ogliaro, F.; Bearpark, M. J.; Heyd, J. J.; Brothers, E. N.; Kudin, K. N.; Staroverov, V. N.; Keith, T. A.; Kobayashi, R.; Normand, J.; Raghavachari, K.; Rendell, A. P.; Burant, J. C.; Iyengar, S. S.; Tomasi, J.; Cossi, M.; Millam, J. M.; Klene, M.; Adamo, C.; Cammi, R.; Ochterski, J. W.; Martin, R. L.; Morokuma, K.; Farkas, O.; Foresman, J. B.; Fox, D. J Gaussian Inc., Wallingford CT, 2016.

2. Gaussian 09, Revision A.02, Frisch, M. J.; Trucks, G. W.; Schlegel, H. B.; Scuseria, G. E.; Robb, M. A.; Cheeseman, J. R.; Scalmani, G.; Barone, V.; Petersson, G. A.; Nakatsuji, H.; Li, X.; Caricato, M.; Marenich, A. V.; Bloino, J.; Janesko, B. G.; Gomperts, R.; Mennucci, B.; Hratchian, H. P., Ortiz, J. V., Izmaylov, A. F., Sonnenberg, J. L., Williams-Young, D., Ding, F., Lipparini, F., Egidi, F., Goings, J., Peng, B., Petrone, A., Henderson, T., Ranasinghe, D., Zakrzewski, V. G., Gao, J., Rega, N., Zheng, G., Liang, W., Hada, M., Ehara, M., Toyota, K. , Fukuda, R., Hasegawa, J., Ishida, M., Nakajima, T., Honda, Y., Kitao, O., Nakai, H., Vreven, T., Throssell, K., Montgomery, J. A., Peralta, Jr., J. E., Ogliaro, F., Bearpark, M., Heyd, J. J., Brothers, E., Kudin, K. N., Staroverov, V. N., Keith, T., Kobayashi, R., Normand, J., Raghavachari, K., Rendell, A., Burant, J. C., Iyengar, S. S., Tomasi, J., Cossi, M., Millam, J. M., Klene, M., Adamo, C., Cammi, R., Ochterski, J. W., Martin, R. L., Morokuma, K., Farkas, O., Foresman, J. B., Fox, D.J., Gaussian Inc., Wallingford CT, 2016.

3. F. Neese, The ORCA program system. *Wiley Interdiscip. Rev. Comput. Mol. Sci.* **2021**, *2*, 73–78, doi: 10.1002/wcms.81

4. F. Neese, Software update: The ORCA program system—Version 5.0. *Wiley Interdiscip. Rev. Comput. Mol. Sci.* **2022**, *12*, e1606, doi: 10.1002/wcms.1606

5. G. M. J. Barca, C. Bertoni, L. Carrington, D. Datta, N. De Silva, J. E. Deustua, D. G. Fedorov, J. R. Gour, A. O. Gunina, E. Guidez, T. Harville, S. Irle, J. Ivanic, K. Kowalski, S. S. Leang, H. Li, W. Li, J. J. Lutz, I. Magoulas, J. Mato, V. Mironov, H. Nakata, B. Q. Pham, P. Piecuch, D. Poole, S. R. Pruitt, A. P. Rendell, L. B. Roskop, K. Ruedenberg, T. Sattasathuchana, M. W. Schmidt, J. Shen, L. Slipchenko, M. Sosonkina, V. Sundriyal, A. Tiwari, J. L. Galvez Vallejo, B. Westheimer, M. Włoch, P. Xu, F. Zahariev, M. S. Gordon, Recent developments in the general atomic and molecular electronic structure system. *J. Chem. Phys.* **2020,** *152*, 154102, doi: 10.1063/5.0005188

6. V. Mironov, K. Komarov, J. Li, I. Gerasimov, H. Nakata, M. Mazaherifar, K. Ishimura, W. Park, A. Lashkaripour, M. Oh, M. Huix-Rotllant, S. Lee, C. Ho Choi, *OpenQP: A Quantum Chemical Platform Featuring MRSF-TDDFT with an Emphasis on Open-Source Ecosystem. J. Chem. Theory Comput.* **2024** *20*, 9464-9477, doi: 10.1021/acs.jctc.4c01117

7. W. Park, K. Komarov, S. Lee, C. Ho Choi, Mixed-Reference Spin-Flip Time-Dependent Density Functional Theory: Multireference Advantages with the Practicality of Linear Response Theory. *J. Phys. Chem. Lett.* **2023** *14*, 8896-8908, doi: 10.1021/acs.jpclett.3c02296

8. S. Lee, M. Filatov, S. Lee, C. Ho Choi, Eliminating spin-contamination of spin-flip time dependent density functional theory within linear response formalism by the use of zeroth-order mixed-reference (MR) reduced density matrix. *J. Chem. Phys.* **2018** *149* (10), 104101, doi: 10.1063/1.5044202

9. S. Lee, E. E. Kim, H. Nakata, S. Lee, C. Ho Choi, Efficient implementations of analytic energy gradient for mixed-reference spin-flip time-dependent density functional theory (MRSF-TDDFT). *J. Chem. Phys.* **2019** *150*, 184111, doi: 10.1063/1.5086895

10. T. Lu, F. Chen, Multiwfn: A multifunctional wavefunction analyzer. *J. Comput. Chem.* **2012**, *33*, 580–592, doi: 10.1002/jcc.22885

11. W.Humphrey, A. Dalke, K. Schulten, VMD: Visual molecular dynamics. *J. Mol. Graph.* **1996**, *14*, 33–38, doi: 10.1016/0263-7855(96)00018-5

12. A. D. Becke, Density-functional thermochemistry. III. The role of exact exchange. *J. Chem. Phys.*, **1993** *98,* 5648-5652, doi: 10.1063/1.464913

13. T. Yanai, D. Tew, and N. Handy, A new hybrid exchange-correlation functional using the Coulomb-attenuating method (CAM-B3LYP). *Chem. Phys. Lett.*, **2004** *393,* 51-57, doi: 10.1016/j.cplett.2004.06.011

14. J.-D. Chai and M. Head-Gordon, Long-range corrected hybrid density functionals with damped atom-atom dispersion corrections. *Phys. Chem. Chem. Phys.*, **2008** *10,* 6615-20. doi: 10.1039/B810189B

15. M. Müller, A. Hansen, S. Grimme; ωB97X-3c: A composite range-separated hybrid DFT method with a molecule-optimized polarized valence double-ζ basis set. *J. Chem. Phys.* **2023** *158*, 014103, doi: 10.1063/5.0133026

16. A. D. McLean and G. S. Chandler, Contracted Gaussian-basis sets for molecular calculations. 1. 2nd row atoms, Z=11-18. *J. Chem. Phys.*, **1980** *72,* 5639-5648, doi: 10.1063/1.438980

17. K. Raghavachari, J. S. Binkley, R. Seeger, and J. A. Pople, Self-Consistent Molecular Orbital Methods. 20. Basis set for correlated wave-functions. *J. Chem. Phys.*, **1980** *72,* 650-654, doi: 10.1063/1.438955

18. S. Miertuš, E. Scrocco, and J. Tomasi, Electrostatic Interaction of a Solute with a Continuum. A Direct Utilization of ab initio Molecular Potentials for the Prevision of Solvent Effects. *Chem. Phys.*, **1981** *55,* 117-129, doi: 10.1016/0301-0104(81)85090-2

19. S. Miertuš and J. Tomasi, Approximate Evaluations of the Electrostatic Free Energy and Internal Energy Changes in Solution Processes. *Chem. Phys.*, **1982** *65,* 239-245, doi: 10.1016/0301-0104(82)85072-6

20. J. L. Pascual-Ahuir, E. Silla, and I. Tuñón, GEPOL: An improved description of molecular-surfaces. 3. A new algorithm for the computation of a solvent-excluding surface. *J. Comp. Chem.*, **1994** *15,* 1127-1138, doi: 10.1002/jcc.540151009

21. C. H. Suresh, G. S. Remya, P. K. Anjalikrishna, Molecular electrostatic potential analysis: A powerful tool to interpret and predict chemical reactivity. *WIREs Comput Mol Sci.* **2022**, *12*, e1601, doi: 10.1002/wcms.1601

22. E. R. Johnson, S. Keinan, P. Mori-Sánchez, J. Contreras-García, A. J. Cohen, Y. Weitao, Revealing noncovalent interactions. *J. Am. Chem. Soc.* **2010**, *132*, 6498–6506, doi: 10.1021/ja100936w

23. S. Emamian, T. Lu, H. Kruse, H. Emamian, Exploring Nature and Predicting Strength of Hydrogen Bonds: A Correlation Analysis Between Atoms-in-Molecules Descriptors, Binding Energies, and Energy Components of Symmetry-Adapted Perturbation Theory. *J. Comput. Chem.* **2019**, *40*, 2868–2881, doi: 10.1002/jcc.26068

24. R. F. W. Bader, Atoms in Molecules. *Acc. Chem. Res.* **1985**, *18*, 9–15, doi: 10.1021/ar00109a003

25. T. H. Dunning Jr., Gaussian basis sets for use in correlated molecular calculations. I. The atoms boron through neon and hydrogen. *J. Chem. Phys.*, **1989** *90*, 1007-1023, doi: 10.1063/1.456153

26. A. D. Becke, A new mixing of Hartree–Fock and local density-functional theories. *J. Chem. Phys.* **1993** *98*, 1372–1377, doi: 10.1063/1.464304

27. F. Weigend and R. Ahlrichs, Balanced basis sets of split valence, triple zeta valence and quadruple zeta valence quality for H to Rn: Design and assessment of accuracy. *Phys. Chem. Chem. Phys.*, **2005** *7*, 3297-3305, doi: 10.1039/B508541A

28. F. Weigend, Accurate Coulomb-fitting basis sets for H to Rn. *Phys. Chem. Chem. Phys.*, **2006** *8,* 1057-1065, doi: 10.1039/B515623H

29. K. Komarov, W. Park, S. Lee, M. Huix-Rotllant, C. Ho Choi, Doubly Tuned Exchange–Correlation Functionals for Mixed-Reference Spin-Flip Time-Dependent Density Functional Theory. *J. Chem. Theory Comput.* **2023** *19*, 7671-7684, doi: 10.1021/acs.jctc.3c00884

30. A. Nenov, T. Cordes, T. T. Herzog, W. Zinth, R. de Vivie-Riedle, Molecular Driving Forces for Z/E Isomerization Mediated by Heteroatoms: The Example Hemithioindigo. *J. Phys. Chem. A,* **2010** *114*, 13016-13030, doi: 10.1021/jp107899g

31. As described in the GAMESS manual, this is implemented as 50% exact exchange, 50% B88 exchange, with LYP correlation.

32. R. Cammi, B. Mennucci, J. Tomasi, Fast Evaluation of Geometries and Properties of Excited Molecules in Solution: A Tamm-Dancoff Model with Application to 4-Dimethylaminobenzonitrile. *J. of Phys. Chem. A*, **2000** *104*, 5631–5637, doi:10.1021/jp000156l.

33. Y.Wang, R.Lu, J. Yao, H. Li, 1,5,7-Triazabicyclo[4.4.0]dec-5-ene Enhances Activity of Peroxide Intermediates in Phosphine-Free alpha-Hydroxylation of Ketones. *Angew. Chem. Int. Ed.* **2021**, *60*, 6631-6638, doi:10.1002/anie.202014478

34. S. Wiedbrauk, T. Bartelmann, S. Thumser, P. Mayer, H. Dube, Simultaneous complementary photoswitching of hemithioindigo tweezers for dynamic guest relocalization. *Nat. Commun.* **2018**, *9*, 1456, doi: 10.1038/s41467-018-03912-7

35. M. Guentner, M. Schildhauer, S. Thumser, P. Mayer, D. Stephenson, P. J. Mayer, H. Dube, Sunlight-powered kHz rotation of a hemithioindigo-based molecular motor. *Nat. Commun.* **2015**, *6*, 8406, doi: 10.1038/ncomms9406
